# Comparative analysis of the circadian rhythm genes *period* and *timeless* in *Culex pipiens* Linnaeus, 1758 (Diptera, Culicidae)

**DOI:** 10.3897/CompCytogen.v10i4.7582

**Published:** 2016-10-10

**Authors:** Elena V. Shaikevich, Ludmila S. Karan, Marina V. Fyodorova

**Affiliations:** 1 Vavilov Institute of General Genetics, Gubkin str., 3, 119991, Moscow, Russia; 2 Central Research Institute of Epidemiology, Novogireevskaya 3a, Moscow, 111123 Russia

**Keywords:** Culex pipiens, circadian rhythm genes, *period*, *timeless*, natural selection

## Abstract

Nucleotide sequences of the circadian rhythm genes, *period* and *timeless*, were studied for the first time in mosquitoes *Culex pipiens* Linnaeus, 1758. In this work we evaluated variations of the studied genome fragments for the two forms of *Culex pipiens* (forma “pipiens” – mosquitoes common for aboveground habitats, forma “molestus” – underground mosquitoes). We compared *Culex pipiens* from Russia with transatlantic *Culex pipiens* and subtropical *Culex quinquefasciatus* Say, 1823. Our results show that intraspecies variability is higher for the gene *period* than for the gene *timeless*. The revealed substitutions in nucleotide sequences and especially in amino acid sequences grouped the individuals of the two forms into distinct clusters with high significance. The detected fixed amino acid substitutions may appear essential for functioning of the circadian rhythm proteins in *Culex pipiens*, and may be correlated with adaptations of the taxa within the group *Culex pipiens*. Our results suggest that natural selection favors fixed mutations and the decrease in diversity of the genes *period* and *timeless* in mosquitoes of the Culex pipiens f. “molestus” compared with the Culex pipiens f. “pipiens”, is probably correlated with adaptive features of Culex pipiens f. “molestus”. The studied genome regions may be considered as promising molecular-genetic markers for identification, population and phylogenetic analysis of similar species and forms of the *Culex pipiens* complex.

## Introduction

The *Culex pipiens* Linnaeus, 1758 complex considered by some authors as a ‘polytypic species’ includes up to seven morphologically identical or very similar forms (Harbach et al. 1984, 1985, Vinogradova 2000). By the second half of the 20^th^ century, the taxonomic status of these forms changed several times from species to subspecies and back. At present only two species, *Culex pipiens* Linnaeus, 1758, and *Culex quinquefasciatus* Say, 1823 have been left within the *Culex pipiens* complex based on morphological similarity (Harbach 2012). Both species are known as bridge-vectors of West Nile and Saint Louis encephalitis flaviviruses, the etiological agents of dangerous human diseases (Vinogradova 2000). The medical significance of the *Culex pipiens* complex generates much interest in its studies, including taxonomy.

Only one species of the *Culex pipiens* complex, *Culex pipiens*, has been found in Russia. This species includes two forms, Culex pipiens f. “pipiens” and Culex pipiens f. “molestus”, originally described as distinct species (Harbach et al. 1984, 1985). *Culex pipiens* forms designation is provided in accordance with the rules of International Code of Zoological Nomenclature (http://www.iczn.org/iczn/index.jsp). The two forms are morphologically identical, but have notably distinct biological features. The mosquitoes Culex pipiens f. “pipiens” are anautogenous (females require a blood meal to mature each egg raft), mate in swarms, oviposit in a wide variety of natural and manmade habitats, feed preferentially on avian hosts and enter diapause to overwinter (Vinogradova 2000). In contrast Culex pipiens f. “molestus” are autogenous (females oviposit the first egg raft without bloodmeal), develop without winter diapause in urban flooded basements and tunnels, feed preferentially on mammal hosts and are able to mate in a confined space. The specific features of reproduction and development of the two forms has resulted in their spatial isolation in moderate climate areas, suggesting genetic isolation. This suggestion is confirmed by the isoenzyme analysis of autogenous and anautogenous populations of *Culex pipiens* from England (Byrne and Nichols 1999), Russia (Lopatin 2000) and Germany (Weitzel et al. 2009) as well as by study of populations from Europe with CQ11 assay (Bahnck and Fonseca 2006). The results of these investigations showed that in these regions the forms are genetically distinct, with no or poor gene flow between populations of different forms. However, in the Mediterranean area, in N Africa and the Middle East, both autogenous and anautogenous specimens develop in the same pools. These populations display highly variable autogeny rates, from 10-90% in Egypt (Gad et al. 1995) to 4–55% in Israel (Nudelman et al. 1988), and both autogenous and anautogenous females were encountered in the progenies of autogenous or anautogenous female parents (Gad et al. 1995). Consequently, the question of divergence of the two forms in moderate climates remains still unclear.

Among the specific behavioral/physiological traits which remained up to now the important criteria for defining populations of Culex pipiens f. “pipiens” and Culex pipiens f. “molestus”, differences in mating behavior are under the special interest. Mating activity of Culex pipiens f. “pipiens” is restricted within the crepuscular period when males aggregate in swarms where they copulate with virgin females attracted to a swarm (Ivanov 1984, Fyodorova and Serbenyuk 1999, Vinogradova 2000). In contrast, males of Culex pipiens f. “molestus” never swarm and have irregular locomotor and mating activity (Shinkawa et al. 1994). Such temporal differences in mating activity may represent the temporal isolation between two forms.

In insects the rhythms of mating activity are controlled by endogenous circadian clocks, which are under genetic control (Konopka and Benzer 1971, Sakai and Ishida 2001, Tauber et al. 2003). The differences in the daily timing of mating activity are documented in many sympatric sibling insect species, e.g in tephritid fruit flies (An et al. 2002, 2004), in *Drosophila* Fallen, 1923 species (Sakai and Ishida 2001, Tauber et al. 2003), sand fly species (Rivas et al. 2008), in *Nasonia* Ashmead, 1904 wasps (Bertossa et al. 2013), in cricket species (Fergus and Shaw 2013). Intra-specific differences in the rhythms of mating activity were revealed also between populations or strains, e.g. in fly *Bactrocera cucurbitae* Coquilletl, 1849 (Fuchikawa et al. 2010) and mosquitoes of *Anopheles cruzii* Dyar and Knab, 1908 complex (Rona et al. 2010).

Clock genes, especially *period* and *timeless*, play an essential role in regulation of mating rhythms in insects. In *Drosophila*, null mutants of the clock gene *period* (*per*) lost the circadian rhythm in mating activity (Sakai and Ishida 2001). Similar effects have been described for gene *timeless* (*tim*) in *Drosophila* and for gene *per* in *Grillus bimaculatus* De Geer, 1773 (Sehgal et al. 1994, Moriyama et al. 2008). The analysis of mating activity in transformant lines carrying *per* transcription units derived from *Drosophila melanogaster* Meigen, 1930 or *Drosophila pseudoobscura* Frolova & Astaurov, 1929, showed that *per* controls species-specific mating rhythms, at least in flies (Tauber et al. 2003).

It may be suggested that differences in the rhythm of mating activity in two forms of *Culex pipiens* resulted from the variations in circadian clocks genes. To test this hypothesis, we selected the genes *per* and *tim*. The aim of our work was to study variable nucleotide sequences in these genes, and to estimate the possible evolutionary significance of the detected variations.

## Methods

The larvae of mosquitoes of both intraspecific forms were collected mostly in August 2006 in Volgograd City and nearby areas. The sampling sites, methods of larvae collection and rearing in lab, and methods of evaluating autogenity have been described earlier (Fedorova and Shaikevich 2013). The DNA of mosquitoes collected in the underground sampling sites in Nizhny Novgorod, Moscow and St Petersburg, as well as in aboveground sampling site Iksha, Moscow region, was used to analyze the diversity of the first exon of the gene *tim*. The methods of mosquito sampling at these sites have been described earlier (Vinogradova and Shaikevich 2007).

### DNA isolation and analysis

The DNA was isolated using the kit DIAtom™ DNA Prep (Isogen Russia). Each of the amplification reactions used 0.1 μg of the total DNA. The polymerase chain reaction (PCR) was run on the thermocycler GeneAmpR PCR System 2700 (Applied Biosystems USA), with amplification Encyclo PCR kit (Evrogen Russia), following the manufacturer’s instructions. For PCR and sequencing of amplification products, specific primers were constructed which were complementary to the conserved sequences of exons in the published sequences of the genes *period* and *timeless* from the total genome of a similar species *Culex quinquefasciatus* (Vector Base Gene ID CPIJ007193 and CPIJ007082, respectively) (Arensburger et al. 2010). When the first sequences were obtained, the primers were constructed basing on DNA sequences of *Culex pipiens*. The PCR conditions were adjusted using the program Oligo6 (http://www.oligo.net/): primary denaturing 95°C - 5 min; 35 cycles at 95°C - 30 s, Tm (for each primer pair) - 1 min, 72°C - 1,5 min; final synthesis at 72°C for 7 min. Primer sequences and annealing temperatures for the PCR are shown in Table 1. Higher temperature was used if two primers in the pair had different annealing temperatures. Negative control was run for all amplification reactions. The DNA of introns was analysed by direct sequencing of amplicons without cloning. Amplified fragments of the genes *per* and *tim* were purified from the gel using QIAquick Gel Extraction kit (Qiagen USA). The fragments were cloned using the kit pGEM-T Easy Vector Systems (Promega USA); the DNA of the three clones for each individual mosquito was sequenced using the equipment ABI PRISM 310 and the BigDye Termination kit (Applied Biosystems USA), according to the manufacturer’s instructions and deposited to GenBank under accession numbers: KU133680-KU133745. The sequences of separate exons of each clone were combined into a single sequence. Nine combined sequences from individual Culex pipiens f. “pipiens” and nine combined sequences from individual Culex pipiens f. “molestus” were investigated for each of the two genes studied, *per* and *tim*. Extended study of the coding sequences of exon 1 of the gene *tim* in two forms of *Culex pipiens* was performed using the DNA from the 26 individual mosquitoes Culex pipiens f. “molestus” and 17 individual mosquitoes Culex pipiens f. “pipiens”. 21 new different haplotypes are submitted to GenBank (KU997646 - KU997666).

**Table 1.** Primers constructed to study the genes *per* and *tim*.

primer	sequence	Tm (°C)	region
PerF2	5’-AGTTCCAAATCGCGCCACAG-3’	54	*per* exon 2
PerR2	5’-TTGGGTTTGCTCGCTTCGTTC-3’	54	*per* exon 2
PerF3	5’-ACAATGCATAGCCAACCGCAAG-3’	55	*per* exon 3
PerR3	5’-GTTCGTCCCTTGACCATGATC-3’	54	*per* exon 3
PerF4	5’-AACGGCTGTTATCTCGTACTG-3’	52	*per* exon 4
PerR4	5’-GCATCGCGTGGTACATCATCG-3’	56	*per* exon 4
TimF1	5’-AATGGTTGCTAGCGAATCCG-3’	52	*tim* exon1
TimR1	5’-AGTAGAGTTCTCGACACCCG-3’	54	*tim* exon1
TimF5	5’-GATTGGTCGGATTTGATTGAG-3’	50	*tim* exon5
TimR5	5’-GTATGTCATCAACCGCCTTG-3’	52	*tim* exon5
TimF5-1	5’-GGAAACCAGCAAAAGACTCG-3’	52	*tim* intron5-6, exon6, intron6-7
TimR7	5’-TACGAGAGCACGTTGAACTG-3’	52	*tim* intron5-6, exon6, intron6-7
TimF7	5’-ACATACTGTACAACATTGCCCTG-3’	53	*tim* intron7-8
TimR8	5’-TCAGGTCGAACTTGATGATG-3’	50	*tim* intron7-8
TimF9	5’-GCTGCGGCCGAAAGCGCCAG-3’	60	*tim* intron9-10
TimR10	5’-ATTTCCATCGCTCGTGTGCTG-3’	54	*tim* intron9-10

### Data analysis

The DNA sequences were translated into amino acids sequences using ExPASy software (Swiss Institute of Bioinformatics), and compared with amino acids sequences of *Culex quinquefasciatus* (Arensburger et al. 2010) and *Culex pipiens* from the USA (Meuti et al. 2015) using programs MAFFT (http://mafft.cbrc.jp/alignment/server/) and MEGA6 (Tamura et al. 2013). Evolutionary analysis was run using MEGA6. Maximum Composite Likelihood model (Tamura et al. 2004) and Kimura 2-parameter model (Kimura 1980) were used to describe the nucleotide substitution pattern. Tables below show the data obtained using the Maximum Composite Likelihood model. The Kimura 2-parameter model produced somewhat higher estimates. The optimal model describing evolutionary patterns was found using the option ‘Find best DNA/Protein substitution model’ in MEGA6. For our data, the Jones-Taylor-Thornton (JTT) model (Jones et al. 1992) showed the lowest BIC (Bayesian Information Criterion) scores for amino acid sequences and was selected to describe the amino acids substitution pattern. For estimating polymorphism within each group and evolutionary divergence between each group the number of base substitutions per site from averaging over all sequence pairs was calculated, all positions containing gaps and missing data were eliminated. Codon positions included were 1st+2nd+3rd+Noncoding. For the estimation of Maximum Likelihood Estimate of Transition/Transversion Bias (R) substitution pattern and rates were estimated under the Kimura 2-parameter model.

Phylogeny analysis was run in MEGA6. The evolutionary history was inferred using the Neighbor-Joining method. The percentage of replicate trees in which the associated taxa clustered together in the bootstrap test (1000 replicates) is shown next to the branches. All ambiguous positions were removed for each sequence pair.

Natural selection and the probability of rejecting the null hypothesis of strict-neutrality (*d*N = *d*S) was evaluated using MEGA6. For these purposes was used a codon-based Z-test (MEGA6). For a pair of sequences, this is done by first estimating the number of synonymous substitutions per synonymous site (*d*S) and the number of nonsynonymous substitutions per nonsynonymous site (*d*N), and their variances: Var(*d*S) and Var(*d*N), respectively. With this information, we tested the null hypothesis that there is no impact of selection (*d*N = *d*S) and the probability (*P*) of rejecting the null hypothesis of strict-neutrality. Also was tested an alternative hypothesis of purifying selection (*d*N < *d*S) and the probability of rejecting the null hypothesis of strict-neutrality in favor of the alternative hypothesis using a codon-based Z-test (MEGA6). Values of *P* determine statistical significance in a hypothesis test. A low *P* value suggests that sample provides enough evidence for the rejecting of the null hypothesis for the entire population. Values of *P* less than 0.05 are considered significant at the 5% level. The variance of the difference was computed using the analytical method (Kimura 1980). All ambiguous positions were removed for each sequence pair.

## Results

### The gene *period* (*per*) in two forms of *Culex pipiens*

The structure of the gene *per* was studied in three individual Culex pipiens f. “molestus” and in three individual Culex pipiens f. “pipiens”. Coding sequences of the three exons of the gene *per* were analysed: exon 2, 333 bp, exon 3, 738 bp, and exon 4, 1229 bp. In total, the 18 compared sequences spanned each 2300 bp (Suppl. material 1).

**Figure 1.**
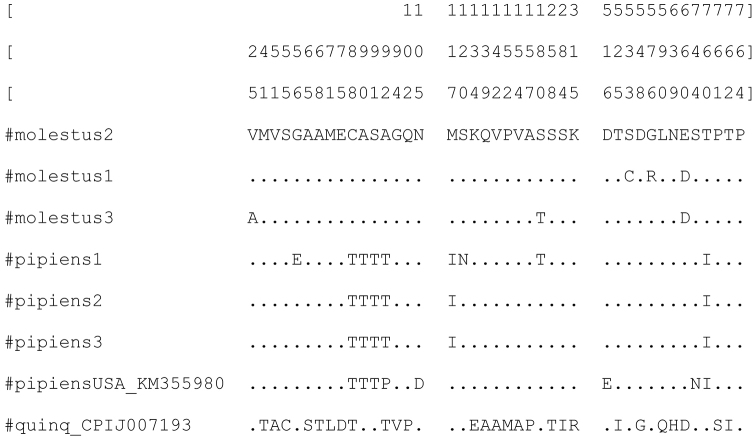
Variable amino acid sites of the gene *period* in *Culex pipiens*. *Culex pipiens* from the USA (KM355980) and *Culex quinquefasciatus* (CPIJ007193) are taken for the comparison. Exons 2 (sites 1-111), 3 (113-358), and 4 (360-768) are separated with blank columns. Positions of the variable sites relative to combined sequences as presented in Suppl. material 1 shown on the top.

In the exon 2 of the gene *per* (333 bp) 11 variable sites were found; six of these substitutions resulted in amino acid substitutions in both forms of *Culex pipiens* (Fig. 1). The exon 3 (738 bp) had 12 variable nucleotide sites, resulting in three amino acid substitutions (Fig. 1). The exon 4 (1229 bp) had 27 variable nucleotide sites, resulting in four amino acid substitutions (Fig. 1). In total, the nucleotide sequence of the three exons of the gene *per* for the both intraspecific forms had 50 (2.2%) variable nucleotide sites and 13 (1.7%) polymorphic amino acid sites, 48 nucleotide sites being parsimony-informative. The estimated Transition/Transversion bias (R) is 2.83. The DNA polymorphism of the gene *per* among individuals of the Culex pipiens f. “pipiens” (0.003) and of the Culex pipiens f. “molestus” (0.002) were both low, variability of the amino acid sequences also was low (Table 2). The genetic distances between two forms of *Culex pipiens* from Volgograd were 0.010 based on nucleotide sequences and 0.011 based on amino acid sequences of the gene *per* (Table 2).

**Table 2.** Estimates of Evolutionary Divergence over *per* and *tim* sequence pairs between *Culex pipiens* complex members.

	AA\NA	gene *period*				gene *timeless*			
		1	2	3	4	1	2	3	4
1	molestus		**0.010**	**0.010**	**0.027**		**0.012**	**0.025**	**0.028**
2	pipiens	0.011		**0.008**	**0.029**	0,008		**0.031**	**0.030**
3	pipiensUSA	0.013	0.008		**0.027**	0.018	0.018		**0.009**
4	quin	0.036	0.036	0.041		0.018	0.020	0.004	

In upper right section in bold: the number of nucleotide base substitutions (NA) per site from averaging over all sequence pairs between groups is shown. All results are based on the pairwise analysis of 20 sequences. There were a total of 2300 positions of *per* gene and 1560 positions of *tim* gene in the final dataset. In lower left section: the number of amino acid substitutions (AA) per site from averaging over all sequence pairs between groups are shown. The analysis involved 20 amino acid sequences. A total of 766 positions of the gene *per* and 520 positions of the gene *tim* were analysed as the final dataset.

### Comparison of the gene *per* for transatlantic *Culex pipiens*

The obtained sequences of the gene *per* of *Culex pipiens* from Volgograd and Culex pipiens f. “pipiens” from the USA (GenBank acc. number KM355980) using BLAST software were compared. The identity of nucleotide sequences of Culex pipiens f. “pipiens” mosquitoes from different continents is 98-99%; 4-16 amino acid substitutions were detected. Pairwise comparison showed that Culex pipiens f. “pipiens” from the USA is slightly different from the Volgograd Culex pipiens f. “pipiens” (0.008) and from Culex pipiens f. “molestus” (0.013); these values are comparable with the differences between the studied Culex pipiens f. “pipiens” and Culex pipiens f. “molestus” (Table 2).

### Comparison of the gene *per* in *Culex pipiens* and *Culex quinquefasciatus*

The identity of the nucleotide sequences of the gene *per* for the two species was 97%. Comparison of the DNA from both forms of *Culex pipiens* and *Culex quinquefasciatus* (CPIJ007193) revealed 113-116 (4.9–5.5%) variable nucleotide sites (Suppl. material 1): 37 nucleotide substitutions were non-synonymous, resulting in amino acid substitutions (Fig. 1). 64 nucleotide substitutions and 24 amino acid substitutions are specific for *Culex quinquefasciatus*, with nine substitutions in each of the exons 2 and 3, and six in exon 4 (Fig. 1). The mean genetic divergence between *Culex pipiens* and *Culex quinquefasciatus* is 0.03 by both DNA and amino acid sequences. The difference between *Culex pipiens* and *Culex quinquefasciatus* is three times higher than the difference between the two forms of *Culex pipiens* (Table 2).

### Gene *timeless* (*tim*) in the two forms of *Culex pipiens*

Using the DNA from the same three individual mosquitoes Culex pipiens f. “molestus” and three individual mosquitoes Culex pipiens f. “pipiens”, the three longest coding sequences of the gene *tim* were studied: exon 1 (1037 bp), exon 5 (376-379 bp), and exon 6 (145 bp). In total, the 18 compared sequences each spanned 1557-1560 bp. (Suppl. material 2).

**Figure 2.**
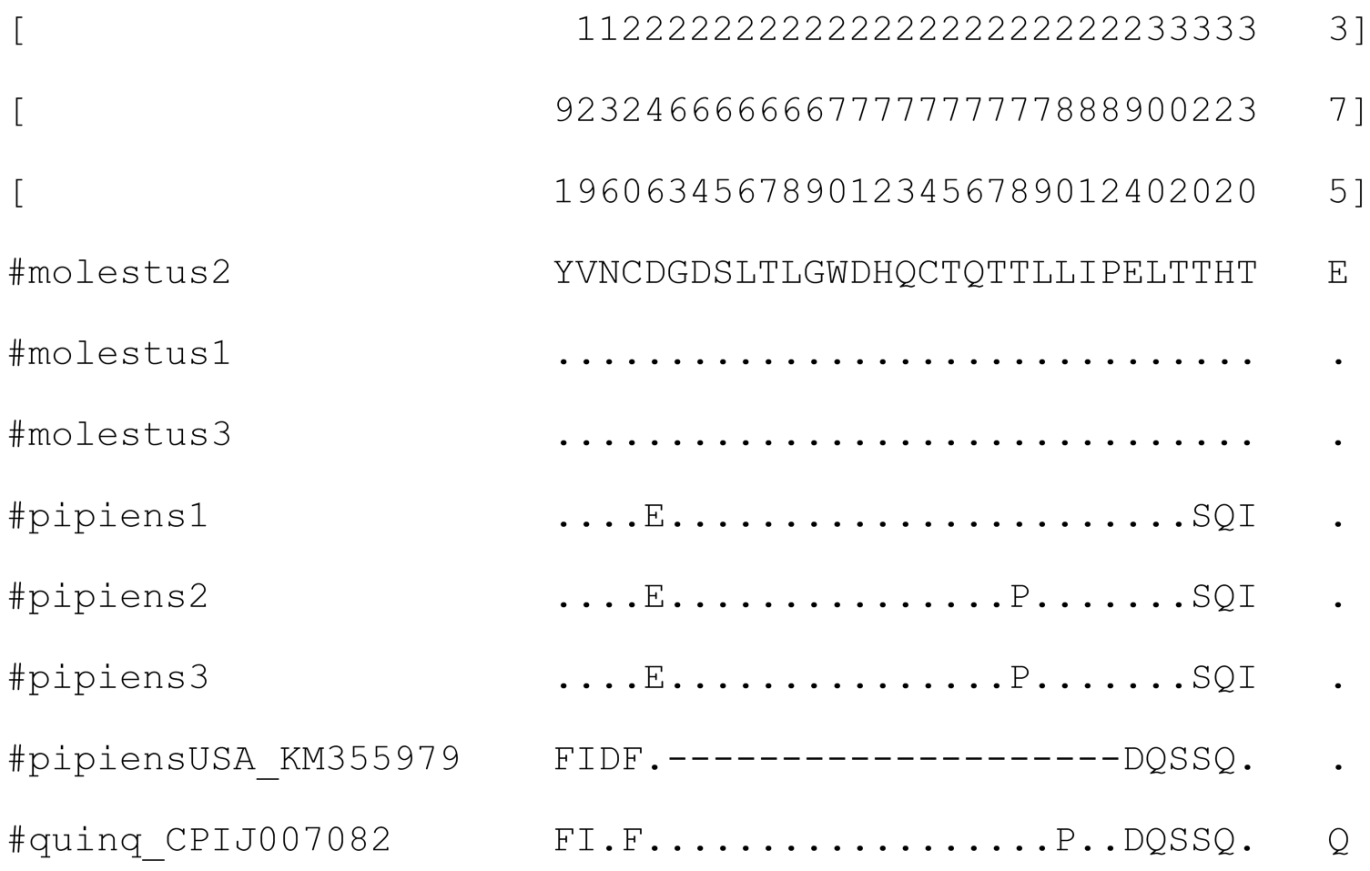
Variations of amino acid sites in the gene *tim* from *Culex pipiens*. *Culex pipiens* from the USA (KM355979), and *Culex quinquefasciatus* (CPIJ007082) are taken for the comparison. Dashes show deletion in exon 1 in *Culex pipiens* from the USA (KM355979). Exon 1 (sites 1-345) and exon 5 (sites 347-472) are separated by blank columns. Positions of the variable sites in combined *tim* sequences shown on the top.

In exon 1 of the gene *tim* (1037 bp) 15 variable nucleotide sites and five variable amino acid sites were found, four of them showing variations only for the Culex pipiens f. “pipiens” and were not found in Culex pipiens f. “molestus” (Fig. 2). In exon 5 (379 bp) five DNA substitutions were found, and in exon 6 (145 bp) there were three variable nucleotide sites; all substitutions in the exons 5 and 6 were synonymous, resulting in similar amino acid sequences for Culex pipiens f. “pipiens” and Culex pipiens f. “molestus” (Suppl. material 2, Fig. 2). The estimated Transition/Transversion bias (R) is 2.79.

Comparing the nucleotide sequences of the gene *tim* for the specimens of Culex pipiens f. “molestus” one variable DNA site was found, the detected nucleotide substitution does not result in amino acid substitution. The aligned DNA sequences of the Culex pipiens f. “pipiens” had 11 variable sites, one mutation resulting in amino acid substitution (Fig. 2). The DNA polymorphism of the gene *tim* among specimens of the Culex pipiens f. “pipiens” (0.0038) was higher than for Culex pipiens f. “molestus” (0.0004), and variability of the amino acid sequences was 0.001 and 0.000, respectively. Comparing the total sequence of the three exons of the gene *tim*, between Culex pipiens f. “pipiens” and Culex pipiens f. “molestus” 23 (1.5%) variable nucleotide sites were found (all 23 sites were parsimony-informative) and six (0.4%) polymorphic amino acid sites. Genetic distance between the two forms was 0.012 for DNA sequences and 0.008 for amino acid sequences (Table 2).

### Comparison of the gene *tim* for the transatlantic *Culex pipiens*

The obtained sequences of the gene *tim* for *Culex pipiens* from Volgograd and Culex pipiens f. “pipiens” from the USA (KM355979) were compared using BLAST software. Identity of nucleotide sequences for mosquitoes of Culex pipiens f. “pipiens” from different continents is 96-97%. We found 7-12 amino acid substitutions. Unexpectedly, we found a 60-bp deletion within the coding sequence of exon 1 in Culex pipiens f. “pipiens” from the USA (KM355979), positions 263-282 in Fig. 2. No similar deletion was found in either of the studied *Culex pipiens* forms from Volgograd and no similar deletions were found in *Culex quinquefasciatus* (CPIJ007082). The genetic distance between Culex pipiens f. “pipiens” from Volgograd and Culex pipiens f. “pipiens” from the USA (KM355979) is 0.025, two times higher than the distance between both forms of *Culex pipiens* in Volgograd 0.012 (Table 2).

### Comparison of the gene *tim* from *Culex pipiens* and *Culex quinquefasciatus*

Comparison of DNA sequences of exons 1, 5 and 6 of the gene *tim* between representatives of the two species, *Culex pipiens* and *Culex quinquefasciatus*, revealed 50 variable sites (see Suppl. material 2), which result in 8-14 amino acid substitutions, eight of which are found only in *Culex quinquefasciatus* (Fig. 2). The genetic distance between the species is 0.029 in the DNA sequences and 0.019 in amino acid sequences (Table 2). A striking similarity should be noted for the gene *tim* from *Culex quinquefasciatus* and Culex pipiens f. “pipiens” from the USA (KM355979). Their amino acid sequences have only three variable sites: 136, 280, and 375 (Fig. 2), their DNA sequences differ in 13 single-nucleotide substitutions and one deletion. The genetic distance for the gene *tim* between *Culex quinquefasciatus* and Culex pipiens f. “pipiens” from the USA (KM355979) is 0.009 based on DNA sequences and 0.004 based on amino acid sequences, lower that the distance between the two forms of *Culex pipiens* in Volgograd (Table 2).

### Extended study of exon 1 of the gene *tim* in two forms of *Culex pipiens*

Our results showed that exon 1 of the gene *tim* in Culex pipiens f. “molestus” differ from that of in Culex pipiens f. “pipiens” (Fig. 2). Contrary to the gene *per*, no shared polymorphisms were found in amino acid sequences of gene *tim* between two forms (Figs 1, 2). To confirm these findings we studied the structure of exon 1 (1037 bp) of the gene *tim* in 23 specimens of Culex pipiens f. “molestus” and 14 specimens of Culex pipiens f. “pipiens” in addition to 6 samples of gene *tim* described above. In total, 43 samples were examined.

The obtained nucleotide sequences showed overlapping peaks in one or more sites for 6 individuals. Four of them were identified as Culex pipiens f. “molestus” and two as Culex pipiens f. “pipiens”. Exon 1 of the gene *tim* of these six samples was studied by cloning and the DNA of the five clones for each specimen was sequenced. In total 79 sequences were obtained for comparative analysis (Suppl. material 3). In five specimens one allele was identical to Culex pipiens f. “pipiens” and other one was identical to Culex pipiens f. “molestus”, i. e. these mosquitoes represented hybrids. In one Culex pipiens f. “molestus” (NN23) the alleles differed by two nucleotide substitutions in 3’end. All hybrids were collected in Volgograd, where both forms develop in the same pools in summer.

**Figure 3.**
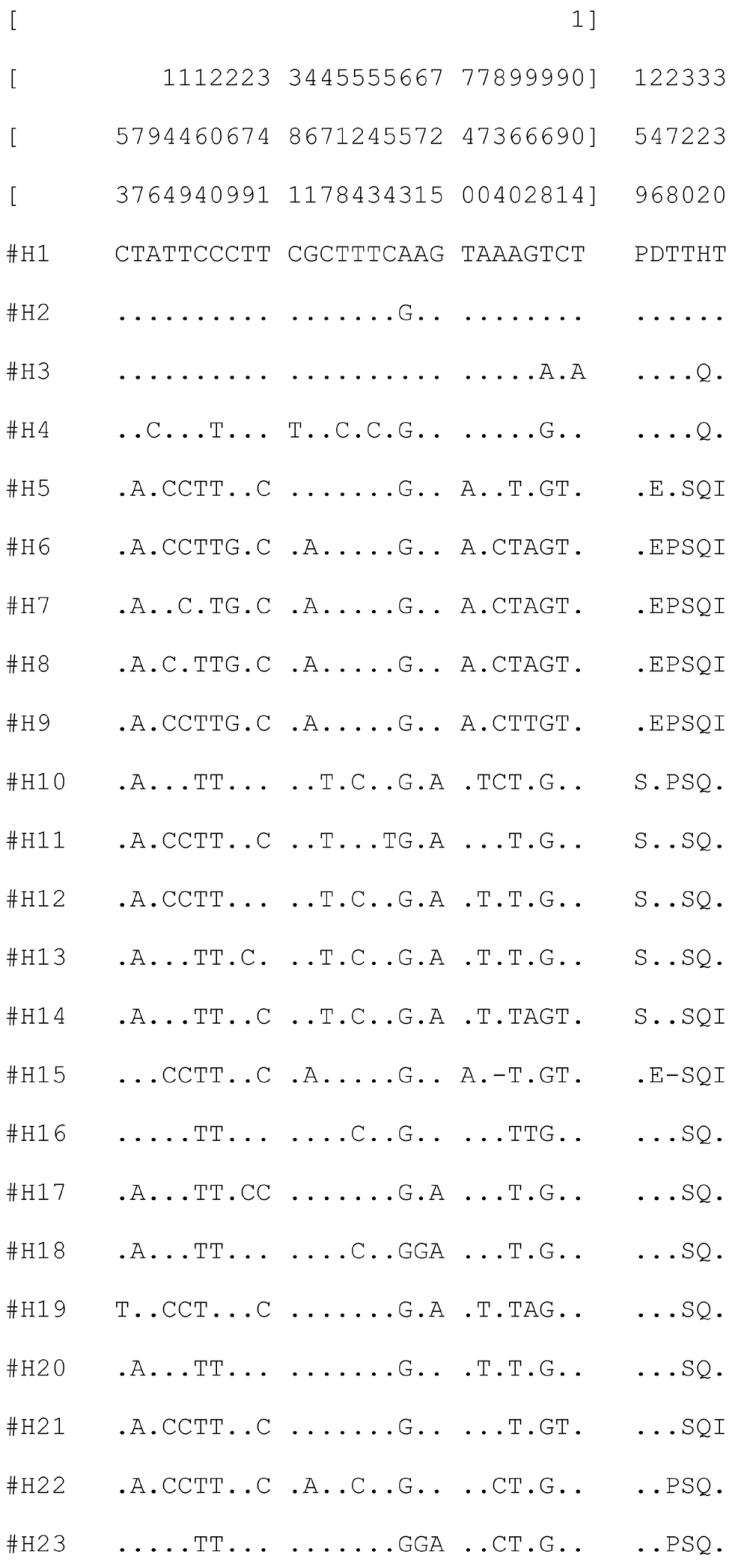
DNA haplotypes and variable amino acid positions in the exon 1 of the gene *tim* from Culex pipiens f. “pipiens” and Culex pipiens f. “molestus”. Haplotypes numbers and variable nucleotide sites are shown on the left. Variable amino acid sites are shown on the right. Only variable haplotypes are shown, all 79 sequences are presented in Suppl. material 3. Positions of the variable sites shown on the top. Dash show deletion of 12 nucleotides (sites 831-842) in exon 1 in Culex pipiens f. “pipiens” from Moscow region.

49 variable nucleotide sites and 23 distinct haplotypes were found in exon 1 of the gene *tim* (Fig. 3). Culex pipiens f. “pipiens” showed 19 haplotypes. Four haplotypes were obtained in Culex pipiens f. “molestus” (H1-H4). Haplotypes H1 and H2 detected in Culex pipiens f. “molestus” from geographically remote locations (Volgograd, Nizhny Novgorod, Moscow and S.-Petersburg) differed by only one synonymous nucleotide substitution A-G at position 653 in Exon 1 of the gene *tim* (Fig. 3). H3 and H4 were detected only in two individuals: H3 combined with H1 (Culex pipiens f. “molestus”) in NN23 and H4 in combination with H11 (Culex pipiens f. “pipiens”) in V219 (Suppl. material 3). Amino acid sequences of Culex pipiens f. “molestus” with H1 and H2 haplotypes differed from Culex pipiens f. “pipiens” by two substitutions Serine (Ser)-Threonine (Thr) and *Glutamine* (Gln)-Histidine (His). Additional substitutions were detected in two specimens with H3 and H4 haplotypes namely the T968A and T968G substitutions in DNA sequences which resulted in Gln in amino acid sequence (Fig. 3, Suppl. material 3). In total, two variations of amino acid sequences were found in Culex pipiens f. “molestus” and 8 in Culex pipiens f. “pipiens” (Fig. 3).

The DNA polymorphism of the exon 1 of gene *tim* among specimens of the Culex pipiens f. “pipiens” (0.007) was ten times higher than for the Culex pipiens f. “molestus” (0.0006), and variability of the amino acid sequences was 0.0053 and 0.0001, respectively. Genetic distance between the two forms was 0.011 for DNA sequences and 0.009 for amino acid sequences. Genetic distance between *Culex pipiens* of both forms and *Culex quinquefasciatus* was 0.029 for DNA and 0.02 for amino acid sequences (Suppl. material 3). The DNA polymorphism, as well as genetic distances between the two forms in extended study of the exon 1 are very close to the results obtained for the three exons of the gene *tim* (see above) (Table 2).

### Variation in non-coding regions of the gene *tim*

The sequences of some non-coding regions were analysed, expecting to find differences not only in coding DNA structure but also in intron size between Culex pipiens f. “pipiens” and Culex pipiens f. “molestus”. The primers were constructed for the conserved sites of the exons using the obtained sequences, and by homology with the gene *tim* from *Culex quinquefasciatus* (CPIJ007082). The sequences of the introns 1-2 (7158 bp in length) and 10-11 (5189 bp), being too long for efficient PCR and sequencing and containing numerous repeats were not analysed. As for the other introns, sequencing of the PCR products showed no variability between two intraspecific forms in intron between exons 5 and 6 (59 bp). In intron 6-7 (61 bp) three variable sites and in intron 7-8 (160 bp) six variable sites were found. Studied introns showed no mutations common with either of the two *Culex pipiens* forms. In the intron 9-10 (167 bp) seven variable sites were found, six of which differed between the two forms (Suppl. material 4). The length of all amplified intron sequences was identical for Culex pipiens f. “pipiens” and Culex pipiens f. “molestus”.

### Phylogenetic analysis

**Figure 4.**
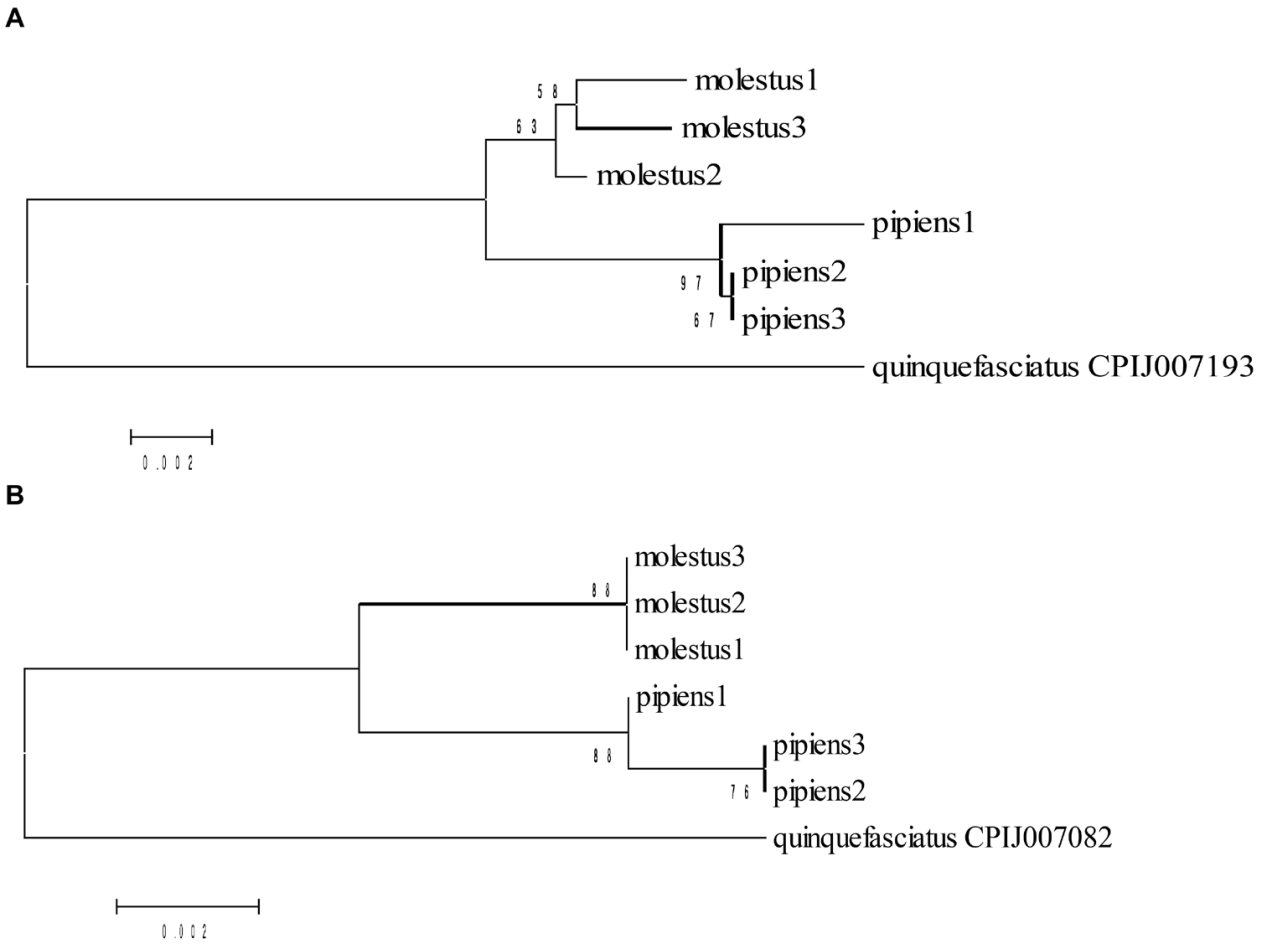
Evolutionary relationships of the studied taxa. Neighbor-joining trees of *Culex pipiens* based on **A** *period* and **B** *timeless* inferred amino acid sequences with the *Culex quinquefasciatus* (CPIJ007193) as the outgroup. Percent bootstrap support based on 1000 replicates. Seven amino acid sequences were analysed with a total of 766 positions of PERIOD (A) and 519 positions of TIMELESS (B) in the final datasets.

Phylogenetic dendrograms were constructed applying the Neighbor-Joining method to amino acid sequences of the three coding regions of genes *per* and *tim*, *Culex quinquefasciatus* was used as an out-group. *Culex quinquefasciatus* and *Culex pipiens* form two well differentiated clusters. Basing on similarity of the gene *per* the individuals of the Culex pipiens f. “pipiens” group together and form a joint cluster with a bootstrap coefficient of 97. The studied specimens of the Culex pipiens f. “molestus” had more polymorphic amino acid sequences, but also are grouped into one cluster with bootstrap coefficient of 63 (Fig. 4). Based on the similarity of the gene *tim*, Culex pipiens f. “pipiens” and Culex pipiens f. “molestus” group into separate clusters with a bootstrap coefficient of 88 (Fig. 4B).

On the dendrogram for the exon 1 of the gene *tim*, constructed using the results of our extended study, most specimens of the Culex pipiens f. “molestus” form separate clusters with a bootstrap coefficient of 96. A separate subcluster is formed by sequences of the hybrid V219 clones with haplotype H4. The studied specimens of the Culex pipiens f. “pipiens” have polymorphic DNA sequences (Suppl. material 3). The dendrogram basing on amino acid sequences shows similar configuration.

### Evolutionary analysis

One way to test whether natural selection is operating on a gene is to compare the relative abundance of synonymous and nonsynonymous substitutions within the gene sequences (Tamura et al. 2013). Analysing evolution of the nucleotide sequences, the Codon-based Test of Neutrality rejected the null hypothesis of strict-neutrality with strong statistical support in both genes (Table 4). Though comparison of some haplotypes within the Culex pipiens f. “pipiens” also shows deviation from neutrality, difference between the forms is considerably higher (Suppl. materials 5, 6). Analysis of *d*N-*d*S between the *per* nucleotide sequences of both intraspecific forms indicates that the probability of rejecting the null hypothesis of strict-neutrality ranges from 0 to 0.015 across the sequences with an overall average of 0.003. Between *tim* nucleotide sequences of the three exons of Culex pipiens f. “pipiens” and Culex pipiens f. “molestus”, the probability of rejecting the null hypothesis of strict-neutrality ranges from 0.003 to 0.017 across the specimens with an overall average of 0.006. Table 4 shows average mean *d*N-*d*S and of the probability of rejecting the null hypothesis of strict-neutrality for each individual.

Analysis of *d*N-*d*S between the 79 nucleotide sequences of exon 1 of the gene *tim* indicates that the probability of rejecting the null hypothesis of strict-neutrality between intraspecific forms ranges from 0.002 to 0.15 across the sequences with an overall average of 0.05. The number of synonymous substitutions per site (*d*S) was higher that the number of non-synonymous substitutions per site (*d*N), indicating Purifying Selection. The probability of rejecting the null hypothesis of strict-neutrality (*d*N = *d*S) in favor of the alternative hypothesis of Purifying Selection (*d*N < *d*S) ranges for the *tim* nucleotide sequences of Culex pipiens f. “pipiens” and Culex pipiens f. “molestus” from 0.001 to 0.10 with an overall average of 0.025 (Suppl. material 7).

## Discussion

For the first time the genetic structure of the circadian rhythm genes (*per* and *tim*) were analysed for mosquitoes Culex pipiens f. “molestus”. Our results have shown that DNA variation in individuals of Culex pipiens f. “molestus” is smaller than in individuals of Culex pipiens f. “pipiens”. Extended study of exon 1 of the gene *tim* revealed 4 DNA haplotypes in Culex pipiens f. “molestus” and 19 haplotypes in Culex pipiens f. “pipiens”. Decrease in DNA variability for the underground mosquitoes of Culex pipiens f. “molestus” was also reported earlier in our study of mitochondrial DNA (Shaikevich and Zakharov 2010).

In coding sequences of both genes *per* and *tim*, variations between physiologically different forms of *Culex pipiens* were found (Table 3). In the gene *per* we found nine polymorphisms shared between the two forms and four fixed differences between the two forms, taking into account Culex pipiens f. “pipiens” from N America (Fig. 1). The gene *tim* had one shared amino acid polymorphisms and one fixed difference between the forms (Fig. 3). Higher variation of the gene *per* is also revealed by comparison of *Culex pipiens* and *Culex quinquefasciatus*: basing on the amino acid sequences, the genetic distances between the species are higher for the gene *per* (0.036) that for the gene of *tim* (0.02).

**Table 3.** Comparison of exons and introns variability between Culex pipiens f. “pipiens” and f. “molestus” from Russia.

Gene	Locus	size (bp)	Variable DNA sites	Differentiating DNA sites	Variable AA sites	Differentiating AA sites
*Per*	exon2	333	11	6	6	4
	exon3	738	12	2	3	1
	exon4	1229	27	9	4	1
*Tim*	exon1	1037	49	2	10	1
	exon5	376–379	5	1	0	0
	exon6	145	3	0	0	0
	intron5-6	59	0	0	-	-
	intron6-7	61	3	0	-	-
	intron7-8	160	6	0	-	-
	intron9-10	167	7	6	-	-

AA - amino acid

**Table 4.** Codon-based Test of Neutrality between Culex pipiens f. “pipiens” and Culex pipiens f. “molestus”.

specimen		gene *period*						gene *timeless*					
		1	2	3	4	5	6	1	2	3	4	5	6
1	pipiens1		-1.950	-2.829	-3.130	-3.682	-3.309		-2.307	-2.009	-2.830	-2.779	-2.679
2	pipiens2	0.108		-2.904	-3.326	-3.865	-3.349	0.034		-1.856	-2.906	-2.859	-2.766
3	pipiens3	0.010	0.008		-3.775	-4.284	-3.788	0.089	0.0663		-2.945	-2.899	-2.805
4	molestus1	0.001	0.003	0.000		-1.534	-0.586	0.0047	0.0023	0.005		-0.333	-0.998
5	molestus2	0.000	0.000	0.000	0.154		-1.427	0.0056	0.0027	0.0058	0.774		-0.665
6	molestus3	0.002	0.003	0.000	0.502	0.15		0.0073	0.0034	0.0075	0.320	0.547	

The test statistic (*d*N - *d*S) is shown above the diagonal. The probability of rejecting the null hypothesis of strict-neutrality (*d*N = *d*S) is shown. Values of *P* less than 0.01 are considered significant at the 1% level. There was a total of 766 positions of gene *per* and of 519 positions of gene *tim* in the final dataset. Evolutionary analyses were conducted in MEGA6.

Culex pipiens f. “pipiens” from N America clusters with Culex pipiens f. “pipiens” from Volgograd basing on comparison of the gene *per* and with *Culex quinquefasciatus* based on comparison of the gene *tim*. It remains unknown whether this is common for all American Culex pipiens f. “pipiens”, shown using microsatellite analysis to differ from the European Culex pipiens f. “pipiens” (Fonseca et al. 2004), or if it is a specific feature of the laboratory line, used to study the genes on circadian rhythm (Meuti et al. 2015).

Genetic structure of the studied genes is polymorphic. However, the revealed substitutions in nucleotide sequences and especially in protein sequences grouped the individuals of the two forms into distinct clusters with high significance, a longer genetic distance separating the cluster of *Culex pipiens* from *Culex quinquefasciatus*. Although the two studied genes differed in variability, the results of analysis of the gene *per*, as well as the gene *tim*, show that the difference between *Culex pipiens* and *Culex quinquefasciatus* are 2.5–3 times higher than the difference between the forms of *Culex pipiens*. The genetic distances again confirm the order of evolutionary events in the *Culex pipiens* complex: the divergence of the form Culex pipiens f. “molestus” from *Culex pipiens* occurred considerably later than the divergence of *Culex pipiens* and *Culex quinquefasciatus* (Barr 1967, Fonseca et al. 2004, Shaikevich and Zakharov 2014).

The non-coding genome sequences are considered to be highly variable. These sequences are often used to search for the markers to differentiate closely related organisms by size of the PCR products. For example, variation in spacers of the ribosomal genes cluster is a base for identification of some mosquito species of the genus *Anopheles* (Nicolescu et al. 2004, Gordeev et al. 2004). In sequences of three *tim* introns no significant difference was found between the forms. For *Aedes albopictus* Skuse, 1894, also no significant difference in the introns of the gene *tim* was reported (Summa et al. 2012).

The Test of Neutrality rejects the null hypothesis of strict-neutrality at *P* < 5% level and imply that both *per* and *tim* loci evolve under strong selective constraint during the divergence of intraspecific forms. Our results suggest that natural selection favored the fixed mutations and the decreased diversity of the genes *per* and *tim* in mosquitoes Culex pipiens f. “molestus” compared with the Culex pipiens f. “pipiens”, probably preserving adaptive features of the form “molestus”. Well-documented data have been reported showing that new native mutations sometimes are rapidly spreading in a population and that polymorphism in one locus may provide adaptive variations in behavioral and morphological phenotypes of the insects in nature (Tauber et al. 2007). The genes involved in circadian rhythms are proved to coordinate seasonal responses, e.g. they initiate the reproductive diapause; malfunctioning of the genes *per* and *tim* was shown to interrupt diapausing of the *Culex pipiens* females (Meuti et al. 2015). We can assume that mutations found in *per* and especially in *tim* genes are related with functioning of the circadian rhythm proteins and contributed to divergence of the forms of *Culex pipiens*. The studied genes are promising candidates to evaluate the genetic basis of different behaviors of the two ecological forms within one subspecies. Further studies of the circadian rhythm genes in mosquitoes of the *Culex pipiens* complex would help to test this assumption.

## Conclusions

Nucleotide sequences of the circadian rhythm genes were studied for the first time in mosquitoes Culex pipiens f. “molestus” and compared with those for Culex pipiens f. “pipiens” and *Culex quinquefasciatus*. These results show that intraspecies variability is higher for the gene *per* than for the gene *tim*. Revealed substitutions in nucleotide sequences and especially in protein sequences grouped the individuals of the two ecological forms of *Culex pipiens* into distinct clusters with high significance. The results suggest that natural selection favored the fixed mutations and the decreased diversity of the genes *per* and *tim* in mosquitoes of the Culex pipiens f. “molestus” compared with the Culex pipiens f. “pipiens”. The detected fixed amino acid substitutions may appear essential for functioning of the circadian rhythm proteins in *Culex pipiens*, and may be related with adaptations of the taxa within the group *Culex pipiens*. Moreover, under natural selection mutations in the key genes of circadian pattern may provide some advantage to the underground Culex pipiens f. “molestus”. The studied genome regions may be considered as promising molecular-genetic markers for identification, population and phylogenetic analysis of similar species and forms of the *Culex pipiens* complex.

## Supplementary materials

Supplementary material 1 Aligned nucleotide sequences of *per* gene. Data type: primary data Explanation note: DNA sequences of three clones of each individual *Culex pipiens* are presented and compared with sequences of *Culex quinquefasciatus* (CPIJ007193) and *Culex pipiens* from the USA (KM355980). This dataset is made available under the Open Database License (http://opendatacommons.org/licenses/odbl/1.0/). The Open Database License (ODbL) is a license agreement intended to allow users to freely share, modify, and use this Dataset while maintaining this same freedom for others, provided that the original source and author(s) are credited.Elena V. Shaikevich, Ludmila S. Karan, Marina V. Fyodorova

Supplementary material 2 Aligned nucleotide sequences of *tim* gene. Data type: primary data Explanation note: DNA sequences of three clones of each individual *Culex pipiens* are presented and compared with sequences of *Culex quinquefasciatus* (CPIJ007193) and *Culex pipiens* from the USA (KM355980). This dataset is made available under the Open Database License (http://opendatacommons.org/licenses/odbl/1.0/). The Open Database License (ODbL) is a license agreement intended to allow users to freely share, modify, and use this Dataset while maintaining this same freedom for others, provided that the original source and author(s) are credited.Elena V. Shaikevich, Ludmila S. Karan, Marina V. Fyodorova

Supplementary material 3 Analysis of the divergent between two forms of *Culex pipiens* based on comparison of the exon 1 of the gene *tim* sequences. Data type: primary data Explanation note: Nucleotide and amino acid sequences of the *tim* gene exon1 in compare with sequence of *Culex quinquefasciatus*. This dataset is made available under the Open Database License (http://opendatacommons.org/licenses/odbl/1.0/). The Open Database License (ODbL) is a license agreement intended to allow users to freely share, modify, and use this Dataset while maintaining this same freedom for others, provided that the original source and author(s) are credited.Elena V. Shaikevich, Ludmila S. Karan, Marina V. Fyodorova

Supplementary material 4 Aligned *tim* nucleotide non-coding sequences. Data type: primary data Explanation note: Intron’s DNA sequences of individual Culex pipiens f. “pipiens” and Culex pipiens f. “molestus” are presented. This dataset is made available under the Open Database License (http://opendatacommons.org/licenses/odbl/1.0/). The Open Database License (ODbL) is a license agreement intended to allow users to freely share, modify, and use this Dataset while maintaining this same freedom for others, provided that the original source and author(s) are credited.Elena V. Shaikevich, Ludmila S. Karan, Marina V. Fyodorova

Supplementary material 5 Codon-based Test of Neutrality for analysis between *per* gene sequences of *Culex pipiens* both forms. Data type: measurement of Z-test value Explanation note: The test statistic (*d*N - *d*S) and the probability of rejecting the null hypothesis of strict-neutrality (*d*N = *d*S) are shown base on the differences between *per* gene sequences of *Culex pipiens* both forms. This dataset is made available under the Open Database License (http://opendatacommons.org/licenses/odbl/1.0/). The Open Database License (ODbL) is a license agreement intended to allow users to freely share, modify, and use this Dataset while maintaining this same freedom for others, provided that the original source and author(s) are credited.Elena V. Shaikevich, Ludmila S. Karan, Marina V. Fyodorova

Supplementary material 6 Codon-based Test of Neutrality for analysis between *tim* gene sequences of *Culex pipiens* both forms. Data type: measurement of Z-test value Explanation note: The test statistic (*d*N - *d*S) and the probability of rejecting the null hypothesis of strict-neutrality (*d*N = *d*S) are shown base on the differences between *tim* gene sequences of *Culex pipiens* both forms. This dataset is made available under the Open Database License (http://opendatacommons.org/licenses/odbl/1.0/). The Open Database License (ODbL) is a license agreement intended to allow users to freely share, modify, and use this Dataset while maintaining this same freedom for others, provided that the original source and author(s) are credited.Elena V. Shaikevich, Ludmila S. Karan, Marina V. Fyodorova

Supplementary material 7 Codon-based Test of of Purifying Selection for analysis between the exon1 of the gene *tim* sequences of *Culex pipiens* both forms. Data type: measurement of Z-test value Explanation note: The test statistic (*d*N - *d*S) and the probability of rejecting the null hypothesis of strict-neutrality (*d*N = *d*S) are shown base on the differences between the exon1 of the gene *tim* sequences of *Culex pipiens* both forms. This dataset is made available under the Open Database License (http://opendatacommons.org/licenses/odbl/1.0/). The Open Database License (ODbL) is a license agreement intended to allow users to freely share, modify, and use this Dataset while maintaining this same freedom for others, provided that the original source and author(s) are credited.Elena V. Shaikevich, Ludmila S. Karan, Marina V. Fyodorova
