# Supplementary material for: Comparative analysis of the circadian rhythm genes period and timeless in Culex pipiens Linnaeus, 1758 (Diptera, Culicidae)
Source: Comp Cytogenet. 2016 Oct 10;10(4):483–504. doi: 10.3897/CompCytogen.v10i4.7582 (PMC5240504; doi:10.3897/CompCytogen.v10i4.7582)
Supplement: Supplementary material 1 — Aligned nucleotide sequences of per gene. [file CompCytogen-010-483-s001.pdf]

Supplemented file 1.

Aligned nucleotide sequences of *per* gene. DNA sequences of three clones of each individual *C. pipiens* are presented and compared with sequences of *C. quinquefasciatus* (CPIJ007193) and *C. pipiens* from the USA (KM355980).

```
[                                                                                             111111 1111111111 ]
[                             11111 1112222222 2222233333 3344444444 4445556777 7777788888 9999000011 1111122222 ]
[                        1567924566 6890012666 7778800145 5900011125 5663692003 4556713559 0144235724 4556703449 ]
[                        5494921224 7032415238 0141559381 9905815654 5199441598 4398234181 3224356820 9584769289 ]
#molestus2-1             CCTTTTGTTC GCGGAAGTGG TTGGCAAAGG GGATTGCATC CTGGGTGCCC GCGGTTTGTG TACAACGCCA CTAGTGTAGT
#molestus2-2             .......... .......... .......... .......... .......... .......... .......... ..........
#molestus2-3             .......... .......... .......... .......... .......... .......... .......... ..........
#molestus1-1             .......... .......... .......... .......... .......... .......... .......... ..G.......
#molestus1-2             .......... .......... .......... .......... .......... .....C.... ..T....... ..G.......
#molestus1-3             .......... .......... .......... .......... .......... .....C.... ..T....... ..G.......
#molestus3-1             ...C...... .......... .......... .......... ...C...... .....C.... .......... ..........
#molestus3-2             ...C...... .......... .......... .......... ...C...... .....C.... .......... ..G.......
#molestus3-3             ...C...... .......... .......... .......... ...C...... .....C.... .......... ..........
#pipiens1-1              .......... AT.....ACA CAA.....AT A......... ...C...... A..A.C.... .......... TCG.C.GG..
#pipiens1-2              ....C..... A......ACA CAA.....AT A......... ...C...... A..A.C.... .......... T.G...G...
#pipiens1-3              .......... AT.....ACA CAA.....AT A......... ...C...... A..A.C.... .......... TCG.C.GG..
#pipiens2-1              .......... .T.....ACA CAA.....AT ....A..... .......... .....CC... .......... TCG.C.GG..
#pipiens2-2              .......... .......ACA CAA.....AT ....A..... .......... .....CC... .......... ..G...G...
#pipiens2-3              .......... .T.....ACA CAA.....AT ....A..... .......... .....CC... .......... TCG.C.GG..
#pipiens3-1              .......... .T.....ACA CAA.....AT .A........ ....A..... .....CC... .......... TCG.C.GG..
```

```
#pipiens3-2             .......... .T.....ACA CAA.....AT .A........ ....A..... .....CC... .......... TCG.C.GG..
#pipiens3-3             ......A... .T.....ACA CAA.....AT .A........ ....A..... .....CC... .......... TCG.C.GG..
#pipiensUSA_KM355980    T......... .......ACA CAC....G.. ...G...... .......... .....CC... .......... TCG.CAG...
#quinq_CPIJ007193       .TC..CACCG .TTAGTCAC. C.ATTCG... ..GG.AGCCA TCC..ATTTT .TA.A.CTAT GG.GGTTTGG T.GACAG.CC


[                       1111111111 1111111111 1111111222 2222222222 222]
[                       3333345555 6666677788 8999999000 0011222222 223]
[                       0335853479 1456726713 3134589578 9967367788 990]
[                       5281953847 4338169621 3527308235 4762187915 284]
#molestus2-1            GTCCAAACCA CAAATGTCTC AACATATGGG TGCTGGGCCC TCC
#molestus2-2            .......... .......... .......... .......... ...
#molestus2-3            .......... .......... .......... ......A... ...
#molestus1-1            .........T .....C..C. ....CC.... .......... ...
#molestus1-2            .........T .....C..C. ....CC.... ......A... ...
#molestus1-3            .........T .....C..C. ....CC.... ......A... ...
#molestus3-1            .......... ........C. ....CC.... ......A... .A.
#molestus3-2            .......... ........C. ....CC.... .......... ...
#molestus3-3            .......... ........C. ....CC.... ......A... .A.
#pipiens1-1             .C.T.G.... .......TC. ..TCC....T ..TC.A.T.. A..
#pipiens1-2             .C.T...... .......TC. ..TCC..... ..TC.A.T.. A..
#pipiens1-3             .C.T.G.... .......TC. ..TCC....T ..TC.A.T.. A..
#pipiens2-1             .C.T.G.... .......TC. ..TCC....T ..TC.A.T.. A..
#pipiens2-2             .C.T...... .......TC. ..TCC..... ..TC.A.T.. A..
#pipiens2-3             .C.T.G.... .......TC. ..TCC....T ..TC.A.T.. A..
```

```
#pipiens3-1             .C.T.G.... ..G.....C. G.TCC....T ...C.A.T.. A..

#pipiens3-2             .C.T.G.... ..G.....C. G.TCC....T ...C.A.T.. A..

#pipiens3-3             .C.T.G.... ..G.....C. G.TCC....T ...C.A.T.. A..

#pipiensUSA_KM355980    AC..GG.A.. ...G....C. G..CC..... ...CAAAT.. .AA

#quinq_CPIJ007193       .CT.GGG.T. TG..C.A.CA .C..CCATAA AC......TT .A.
```

Aligned sequence data, only variable sites are shown. Nucleotide sequences of *per* gene: exon 2 (positions 1-333), exon 3 (positions 337-1074), exon 4 (positions 1078-2306). Positions of the variable sites in combined sequences shown on the top.

```
#MEGA

!Format

   DataType=Nucleotide CodeTable=Standard

   NSeqs=20 NSites=2306

   Identical=. Missing=? Indel=-;

!Domain=Data property=Coding CodonStart=1;

[                                    111 111 111 122 222 222 223 333 333 333 444 444 444 455 555 555 556 666 666 666 777 777 777 ]

[                       123 456 789 012 345 678 901 234 567 890 123 456 789 012 345 678 901 234 567 890 123 456 789 012 345 678 ]

#molestus2-1            GGG GGC AGC AGC GGC TAT GGC GGC AAA AAC AAC ACC CCG GGA GCG ATT CCC GGC CCC ATC ACG CAG CAT CCG GTC ATC

#molestus2-2            ... ... ... ... ... ... ... ... ... ... ... ... ... ... ... ... ... ... ... ... ... ... ... ... ... ...

#molestus2-3            ... ... ... ... ... ... ... ... ... ... ... ... ... ... ... ... ... ... ... ... ... ... ... ... ... ...

#molestus1-1            ... ... ... ... ... ... ... ... ... ... ... ... ... ... ... ... ... ... ... ... ... ... ... ... ... ...

#molestus1-2            ... ... ... ... ... ... ... ... ... ... ... ... ... ... ... ... ... ... ... ... ... ... ... ... ... ...

#molestus1-3            ... ... ... ... ... ... ... ... ... ... ... ... ... ... ... ... ... ... ... ... ... ... ... ... ... ...

#molestus3-1            ... ... ... ... ... ... ... ... ... ... ... ... ... ... ... ... ... ... ... ... ... ... ... ... .C. ...

#molestus3-2            ... ... ... ... ... ... ... ... ... ... ... ... ... ... ... ... ... ... ... ... ... ... ... ... .C. ...
```

```
#molestus3-3             ... ... ... ... ... ... ... ... ... ... ... ... ... ... ... ... ... ... ... ... ... ... ... ... .C. ...
#pipiens1-1              ... ... ... ... ... ... ... ... ... ... ... ... ... ... ... ... ... ... ... ... ... ... ... ... ... ...
#pipiens1-2              ... ... ... ... ... ... ... ... ... ... ... ... ... ... ... ... ... ... ... ... ... ... ... ... ... ...
#pipiens1-3              ... ... ... ... ... ... ... ... ... ... ... ... ... ... ... ... ... ... ... ... ... ... ... ... ... ...
#pipiens2-1              ... ... ... ... ... ... ... ... ... ... ... ... ... ... ... ... ... ... ... ... ... ... ... ... ... ...
#pipiens2-2              ... ... ... ... ... ... ... ... ... ... ... ... ... ... ... ... ... ... ... ... ... ... ... ... ... ...
#pipiens2-3              ... ... ... ... ... ... ... ... ... ... ... ... ... ... ... ... ... ... ... ... ... ... ... ... ... ...
#pipiens3-1              ... ... ... ... ... ... ... ... ... ... ... ... ... ... ... ... ... ... ... ... ... ... ... ... ... ...
#pipiens3-2              ... ... ... ... ... ... ... ... ... ... ... ... ... ... ... ... ... ... ... ... ... ... ... ... ... ...
#pipiens3-3              ... ... ... ... ... ... ... ... ... ... ... ... ... ... ... ... ... ... ... ... ... ... ... ... ... ...
#pipiensUSA_KM355980     ... ... ... ... ..T ... ... ... ... ... ... ... ... ... ... ... ... ... ... ... ... ... ... ... ... ...
#quinq_CPIJ007193        ... ... ... ... ... ... ... ... ... ... ... ... ... ... ... ... ... ..T ... ... ... ... ..C ... ... ...

[                                                    111 111 111 111 111 111 111 111 111 111 111 111 111 111 111 111 111 111 111 ]
[                        788 888 888 889 999 999 999 000 000 000 011 111 111 112 222 222 222 333 333 333 344 444 444 445 555 555 ]
[                        901 234 567 890 123 456 789 012 345 678 901 234 567 890 123 456 789 012 345 678 901 234 567 890 123 456 ]
#molestus2-1             AAG CGC ACA AAG GAC AAG GAT CGC AAG AAG AAA CGC ATC CGG ATG TCG ATC GAG GCC AGC GGG CCG GGA ACG GTG ACG
#molestus2-2             ... ... ... ... ... ... ... ... ... ... ... ... ... ... ... ... ... ... ... ... ... ... ... ... ... ...
#molestus2-3             ... ... ... ... ... ... ... ... ... ... ... ... ... ... ... ... ... ... ... ... ... ... ... ... ... ...
#molestus1-1             ... ... ... ... ... ... ... ... ... ... ... ... ... ... ... ... ... ... ... ... ... ... ... ... ... ...
#molestus1-2             ... ... ... ... ... ... ... ... ... ... ... ... ... ... ... ... ... ... ... ... ... ... ... ... ... ...
#molestus1-3             ... ... ... ... ... ... ... ... ... ... ... ... ... ... ... ... ... ... ... ... ... ... ... ... ... ...
#molestus3-1             ... ... ... ... ... ... ... ... ... ... ... ... ... ... ... ... ... ... ... ... ... ... ... ... ... ...
#molestus3-2             ... ... ... ... ... ... ... ... ... ... ... ... ... ... ... ... ... ... ... ... ... ... ... ... ... ...
```

```
#molestus3-3            ... ... ... ... ... ... ... ... ... ... ... ... ... ... ... ... ... ... ... ... ... ... ... ... ... ...

#pipiens1-1             ... ... ... ... ... ... ... ... ... ... ... ... ... ... ... ... ... ... ... ... ... ... ... ... ... ...

#pipiens1-2             ... ... ... ... ... ... ..C ... ... ... ... ... ... ... ... ... ... ... ... ... ... ... ... ... ... ...

#pipiens1-3             ... ... ... ... ... ... ... ... ... ... ... ... ... ... ... ... ... ... ... ... ... ... ... ... ... ...

#pipiens2-1             ... ... ... ... ... ... ... ... ... ... ... ... ... ... ... ... ... ... ... ... ... ... ... ... ... ...

#pipiens2-2             ... ... ... ... ... ... ... ... ... ... ... ... ... ... ... ... ... ... ... ... ... ... ... ... ... ...

#pipiens2-3             ... ... ... ... ... ... ... ... ... ... ... ... ... ... ... ... ... ... ... ... ... ... ... ... ... ...

#pipiens3-1             ... ... ... ... ... ... ... ... ... ... ... ... ... ... ... ... ... ... ... ... ... ... ... ... ... ...

#pipiens3-2             ... ... ... ... ... ... ... ... ... ... ... ... ... ... ... ... ... ... ... ... ... ... ... ... ... ...

#pipiens3-3             ... ... ... ... ... ... ... ... ... ... ... ... ... ... ... ... ... ... ... ... ..A ... ... ... ... ...

#pipiensUSA_KM355980    ... ... ... ... ... ... ... ... ... ... ... ... ... ... ... ... ... ... ... ... ... ... ... ... ... ...

#quinq_CPIJ007193       ... ... ... ... ... ... ... ... ... ... ... ... ... ... .C. ... ... ... ... ... ..A ... ... ... .C. ...


[                       111 111 111 111 111 111 111 111 111 111 111 111 111 111 122 222 222 222 222 222 222 222 222 222 222 222 ]

[                       555 666 666 666 677 777 777 778 888 888 888 999 999 999 900 000 000 001 111 111 111 222 222 222 233 333 ]

[                       789 012 345 678 901 234 567 890 123 456 789 012 345 678 901 234 567 890 123 456 789 012 345 678 901 234 ]

#molestus2-1            GCG GCT TCC GGA AAT GGC GCC AAC GGC GGA ACC GGG GCC ACG GAG GCA GAC CAG ATG ATG GCT TGT GAG GGG GCG GGA

#molestus2-2            ... ... ... ... ... ... ... ... ... ... ... ... ... ... ... ... ... ... ... ... ... ... ... ... ... ...

#molestus2-3            ... ... ... ... ... ... ... ... ... ... ... ... ... ... ... ... ... ... ... ... ... ... ... ... ... ...

#molestus1-1            ... ... ... ... ... ... ... ... ... ... ... ... ... ... ... ... ... ... ... ... ... ... ... ... ... ...

#molestus1-2            ... ... ... ... ... ... ... ... ... ... ... ... ... ... ... ... ... ... ... ... ... ... ... ... ... ...

#molestus1-3            ... ... ... ... ... ... ... ... ... ... ... ... ... ... ... ... ... ... ... ... ... ... ... ... ... ...

#molestus3-1            ... ... ... ... ... ... ... ... ... ... ... ... ... ... ... ... ... ... ... ... ... ... ... ... ... ...

#molestus3-2            ... ... ... ... ... ... ... ... ... ... ... ... ... ... ... ... ... ... ... ... ... ... ... ... ... ...
```

```
#molestus3-3            ... ... ... ... ... ... ... ... ... ... ... ... ... ... ... ... ... ... ... ... ... ... ... ... ... ...
#pipiens1-1             ... ... ... .A. ... ... ... ..T ... ... ... ... ... ... ... ... ... ... ... ... ... ... ... ... ... ...
#pipiens1-2             ... ... ... .A. ... ... ... ... ... ... ... ... ... ... ... ... ... ... ... ... ... ... ... ... ... ...
#pipiens1-3             ... ... ... .A. ... ... ... ..T ... ... ... ... ... ... ... ... ... ... ... ... ... ... ... ... ... ...
#pipiens2-1             ... ... ... ... ... ... ... ..T ... ... ... ... ... ... ... ... ... ... ... ... ... ... ... ... ... ...
#pipiens2-2             ... ... ... ... ... ... ... ... ... ... ... ... ... ... ... ... ... ... ... ... ... ... ... ... ... ...
#pipiens2-3             ... ... ... ... ... ... ... ..T ... ... ... ... ... ... ... ... ... ... ... ... ... ... ... ... ... ...
#pipiens3-1             ... ... ... ... ... ... ... ..T ... ... ... ... ... ... ... ... ... ... ... ... ... ... ... ... ... ...
#pipiens3-2             ... ... ... ... ... ... ... ..T ... ... ... ... ... ... ... ... ... ... ... ... ... ... ... ... ... ...
#pipiens3-3             ... ... ... ... ... ... ... ..T ... ... ... ... ... ... ... ... ... ... ... ... ... ... ... ... ... ...
#pipiensUSA_KM355980    ... ... ... ... ... ... ... ... ... ... ... ... ... ... ... ... ... ... ... ... ... ... ... ... ... ...
#quinq_CPIJ007193       ... ..C .G. ... ... ... ... ..T ... ... ... ... T.. ... ... A.G ... ... T.. ... ... ... ..C ... ... ...


[                       222 222 222 222 222 222 222 222 222 222 222 222 222 222 222 222 222 222 222 222 222 223 333 333 333 333 ]
[                       333 334 444 444 444 555 555 555 566 666 666 667 777 777 777 888 888 888 899 999 999 990 000 000 000 111 ]
[                       567 890 123 456 789 012 345 678 901 234 567 890 123 456 789 012 345 678 901 234 567 890 123 456 789 012 ]
#molestus2-1            CCC GGA AGT GCG TCG GGT CAG GGC GAA TGT GGG GCT TCG GCG GGT GGT CAC CAA GAA ATG AAC GCG CAG CAA GAA ATG
#molestus2-2            ... ... ... ... ... ... ... ... ... ... ... ... ... ... ... ... ... ... ... ... ... ... ... ... ... ...
#molestus2-3            ... ... ... ... ... ... ... ... ... ... ... ... ... ... ... ... ... ... ... ... ... ... ... ... ... ...
#molestus1-1            ... ... ... ... ... ... ... ... ... ... ... ... ... ... ... ... ... ... ... ... ... ... ... ... ... ...
#molestus1-2            ... ... ... ... ... ... ... ... ... ... ... ... ... ... ... ... ... ... ... ... ... ... ... ... ... ...
#molestus1-3            ... ... ... ... ... ... ... ... ... ... ... ... ... ... ... ... ... ... ... ... ... ... ... ... ... ...
#molestus3-1            ... ... ... ... ... ... ... ... ... ... ... ... ... ... ... ... ... ... ... ... ... ... ... ... ... ...
#molestus3-2            ... ... ... ... ... ... ... ... ... ... ... ... ... ... ... ... ... ... ... ... ... ... ... ... ... ...
```

```
#molestus3-3             ... ... ... ... ... ... ... ... ... ... ... ... ... ... ... ... ... ... ... ... ... ... ... ... ... ...

#pipiens1-1              ... ... ... ... ... ... ... ... ... AC. ... A.C A.. A.. ... ... ... ... ... ... ... ... ... ... ... ...

#pipiens1-2              ... ... ... ... ... ... ... ... ... AC. ... A.C A.. A.. ... ... ... ... ... ... ... ... ... ... ... ...

#pipiens1-3              ... ... ... ... ... ... ... ... ... AC. ... A.C A.. A.. ... ... ... ... ... ... ... ... ... ... ... ...

#pipiens2-1              ... ... ... ... ... ... ... ... ... AC. ... A.C A.. A.. ... ... ... ... ... ... ... ... ... ... ... ...

#pipiens2-2              ... ... ... ... ... ... ... ... ... AC. ... A.C A.. A.. ... ... ... ... ... ... ... ... ... ... ... ...

#pipiens2-3              ... ... ... ... ... ... ... ... ... AC. ... A.C A.. A.. ... ... ... ... ... ... ... ... ... ... ... ...

#pipiens3-1              ... ... ... ... ... ... ... ... ... AC. ... A.C A.. A.. ... ... ... ... ... ... ... ... ... ... ... ...

#pipiens3-2              ... ... ... ... ... ... ... ... ... AC. ... A.C A.. A.. ... ... ... ... ... ... ... ... ... ... ... ...

#pipiens3-3              ... ... ... ... ... ... ... ... ... AC. ... A.C A.. A.. ... ... ... ... ... ... ... ... ... ... ... ...

#pipiensUSA_KM355980     ... ... ... ... ... ... ... ... ... AC. ... A.C A.. C.. ... ... ... ... ... ... ... ... ... ... ... ...

#quinq_CPIJ007193        ... ... ... ... ... ... ... ... ... AC. ... ..C ... A.. ... .T. ..T ... ... ... ... ... ... .C. ..G ...


[                        333 333 333 333 333 333 333 333 333 333 333 333 333 333 333 333 333 333 333 333 333 333 333 333 333 333 ]

[                        111 111 122 222 222 223 333 333 333 444 444 444 455 555 555 556 666 666 666 777 777 777 788 888 888 889 ]

[                        345 678 901 234 567 890 123 456 789 012 345 678 901 234 567 890 123 456 789 012 345 678 901 234 567 890 ]

#molestus2-1             AAC GAA GCG AGC AAA CCC AAA --- GTT GAT GCG GCG ATG CCG TCG AGC TCC GGA GTG GTT CCG ATG GAG GAA GGT GGT

#molestus2-2             ... ... ... ... ... ... ... --- ... ... ... ... ... ... ... ... ... ... ... ... ... ... ... ... ... ...

#molestus2-3             ... ... ... ... ... ... ... --- ... ... ... ... ... ... ... ... ... ... ... ... ... ... ... ... ... ...

#molestus1-1             ... ... ... ... ... ... ... --- ... ... ... ... ... ... ... ... ... ... ... ... ... ... ... ... ... ...

#molestus1-2             ... ... ... ... ... ... ... --- ... ... ... ... ... ... ... ... ... ... ... ... ... ... ... ... ... ...

#molestus1-3             ... ... ... ... ... ... ... --- ... ... ... ... ... ... ... ... ... ... ... ... ... ... ... ... ... ...

#molestus3-1             ... ... ... ... ... ... ... --- ... ... ... ... ... ... ... ... ... ... ... ... ... ... ... ... ... ...

#molestus3-2             ... ... ... ... ... ... ... --- ... ... ... ... ... ... ... ... ... ... ... ... ... ... ... ... ... ...
```

```
#molestus3-3            ... ... ... ... ... ... ... --- ... ... ... ... ... ... ... ... ... ... ... ... ... ... ... ... ... ...

#pipiens1-1             ... ... ... ... ... ... ... --- ... ... ... ..A ..T ... ... .A. ... ... ... ... ... ... ... ... ... ...

#pipiens1-2             ... ... ... ... ... ... ... --- ... ... ... ..A ..T ... ... .A. ... ... ... ... ... ... ... ... ... ...

#pipiens1-3             ... ... ... ... ... ... ... --- ... ... ... ..A ..T ... ... .A. ... ... ... ... ... ... ... ... ... ...

#pipiens2-1             ... ... ... ... ... ... ... --- ... ... ... ..A ..T ... ... ... ... ... ... ... ... ... ... ... ... ...

#pipiens2-2             ... ... ... ... ... ... ... --- ... ... ... ..A ..T ... ... ... ... ... ... ... ... ... ... ... ... ...

#pipiens2-3             ... ... ... ... ... ... ... --- ... ... ... ..A ..T ... ... ... ... ... ... ... ... ... ... ... ... ...

#pipiens3-1             ... ... ... ... ... ... ... --- ... ... ... ..A ..T ... ... ... ... ... ... ... ... ... ... ... ... ...

#pipiens3-2             ... ... ... ... ... ... ... --- ... ... ... ..A ..T ... ... ... ... ... ... ... ... ... ... ... ... ...

#pipiens3-3             ... ... ... ... ... ... ... --- ... ... ... ..A ..T ... ... ... ... ... ... ... ... ... ... ... ... ...

#pipiensUSA_KM355980    G.. ... ... ... ... ... ..- --- ... ... ... ... ... ... ... ... ... ... ... ... ... ... ... ... ... ...

#quinq_CPIJ007193       ... ... ... ... ... ... ..- --- ... ... ... ... ... ... ... ... ... ... ... ... ... ... ... ... ... ...


[                       333 333 333 444 444 444 444 444 444 444 444 444 444 444 444 444 444 444 444 444 444 444 444 444 444 444 ]

[                       999 999 999 000 000 000 011 111 111 112 222 222 222 333 333 333 344 444 444 445 555 555 555 666 666 666 ]

[                       123 456 789 012 345 678 901 234 567 890 123 456 789 012 345 678 901 234 567 890 123 456 789 012 345 678 ]

#molestus2-1            GTA GAG GGG AAA GGT GTT GAG GGG CAG GAA GGT GTC CCG AAG ACG CCC GCC GAT TGT AAC CCG CCG AAA GTC GCC AAA

#molestus2-2            ... ... ... ... ... ... ... ... ... ... ... ... ... ... ... ... ... ... ... ... ... ... ... ... ... ...

#molestus2-3            ... ... ... ... ... ... ... ... ... ... ... ... ... ... ... ... ... ... ... ... ... ... ... ... ... ...

#molestus1-1            ... ... ... ... ... ... ... ... ... ... ... ... ... ... ... ... ... ... ... ... ... ... ... ... ... ...

#molestus1-2            ... ... ... ... ... ... ... ... ... ... ... ... ... ... ... ... ... ... ... ... ... ... ... ... ... ...

#molestus1-3            ... ... ... ... ... ... ... ... ... ... ... ... ... ... ... ... ... ... ... ... ... ... ... ... ... ...

#molestus3-1            ... ... ... ... ... ... ... ... ... ... ... ... ... ... ... ... ... ... ... ... ... ... ... ... ... ...

#molestus3-2            ... ... ... ... ... ... ... ... ... ... ... ... ... ... ... ... ... ... ... ... ... ... ... ... ... ...
```

```
#molestus3-3             ... ... ... ... ... ... ... ... ... ... ... ... ... ... ... ... ... ... ... ... ... ... ... ... ... ...

#pipiens1-1              ... ... ... ... ... ... ... ... ... ... ... ... ... ... ... ... ... ... ... ... ... ... ... ... ... ...

#pipiens1-2              ... ... ... ... ... ... ... ... ... ... ... ... ... ... ... ... ... ... ... ... ... ... ... ... ... ...

#pipiens1-3              ... ... ... ... ... ... ... ... ... ... ... ... ... ... ... ... ... ... ... ... ... ... ... ... ... ...

#pipiens2-1              ... ... ... ... ... ..A ... ... ... ... ... ... ... ... ... ... ... ... ... ... ... ... ... ... ... ...

#pipiens2-2              ... ... ... ... ... ..A ... ... ... ... ... ... ... ... ... ... ... ... ... ... ... ... ... ... ... ...

#pipiens2-3              ... ... ... ... ... ..A ... ... ... ... ... ... ... ... ... ... ... ... ... ... ... ... ... ... ... ...

#pipiens3-1              ... ... ..A ... ... ... ... ... ... ... ... ... ... ... ... ... ... ... ... ... ... ... ... ... ... ...

#pipiens3-2              ... ... ..A ... ... ... ... ... ... ... ... ... ... ... ... ... ... ... ... ... ... ... ... ... ... ...

#pipiens3-3              ... ... ..A ... ... ... ... ... ... ... ... ... ... ... ... ... ... ... ... ... ... ... ... ... ... ...

#pipiensUSA_KM355980     ... ... ... ... ..G ... ... ... ... ... ... ... ... ... ... ... ... ... ... ... ... ... ... ... ... ...

#quinq_CPIJ007193        ... ... ... G.. ..G ... ..A ... GC. ... ... .C. ... ... ... ... ... ... ... ... ... AT. ... .C. ... ...


[                        444 444 444 444 444 444 444 444 444 444 455 555 555 555 555 555 555 555 555 555 555 555 555 555 555 555 ]

[                        677 777 777 778 888 888 888 999 999 999 900 000 000 001 111 111 111 222 222 222 233 333 333 334 444 444 ]

[                        901 234 567 890 123 456 789 012 345 678 901 234 567 890 123 456 789 012 345 678 901 234 567 890 123 456 ]

#molestus2-1             GCG GAG GTT GAG GAC GGA TTT TGC TGC GTA ATT TCG ATG CTG GAC GGG GTT GTT CTG TTT ACG ACG CCC AGT ATT ACG

#molestus2-2             ... ... ... ... ... ... ... ... ... ... ... ... ... ... ... ... ... ... ... ... ... ... ... ... ... ...

#molestus2-3             ... ... ... ... ... ... ... ... ... ... ... ... ... ... ... ... ... ... ... ... ... ... ... ... ... ...

#molestus1-1             ... ... ... ... ... ... ... ... ... ... ... ... ... ... ... ... ... ... ... ... ... ... ... ... ... ...

#molestus1-2             ... ... ... ... ... ... ... ... ... ... ... ... ... ... ... ... ... ... ... ... ... ... ... ... ... ...

#molestus1-3             ... ... ... ... ... ... ... ... ... ... ... ... ... ... ... ... ... ... ... ... ... ... ... ... ... ...

#molestus3-1             ... ... ... ... ... ... ... ... ... ... ... ... ... ... ... ... ... ... ... ... ... ... ... .C. ... ...

#molestus3-2             ... ... ... ... ... ... ... ... ... ... ... ... ... ... ... ... ... ... ... ... ... ... ... .C. ... ...
```

#molestus3-3 ... ... .C. ... ...

#pipiens1-1 ... ... .C. ... ...

#pipiens1-2 ... ... .C. ... ...

#pipiens1-3 ... ... .C. ... ...

#pipiens2-1 ... ...

#pipiens2-2 ... ...

#pipiens2-3 ... ...

#pipiens3-1 ... ...

#pipiens3-2 ... ...

#pipiens3-3 ... ...

#pipiensUSA_KM355980 ... ...

#quinq_CPIJ007193 C.. ... ...

[555 555 555 555 555 555 555 555 555 555 555 555 555 555 555 555 555 556 666 666 666 666 666 666 666 666]

[444 555 555 555 566 666 666 667 777 777 777 888 888 888 899 999 999 990 000 000 000 111 111 111 122 222]

[789 012 345 678 901 234 567 890 123 456 789 012 345 678 901 234 567 890 123 456 789 012 345 678 901 234]

#molestus2-1 CGT AGC TTG GGC TTT CCG AAG GAT ATG TGG CTT GGG AGG TCG TTC ATT GAC TTT GTC CAT CCG AAG GAT CGG GCG ACG

#molestus2-2 ... ...

#molestus2-3 ... ...

#molestus1-1 ... ...

#molestus1-2 ... ...

#molestus1-3 ... ...

#molestus3-1 ... ...

#molestus3-2 ... ...

```
#molestus3-3            ... ... ... ... ... ... ... ... ... ... ... ... ... ... ... ... ... ... ... ... ... ... ... ... ... ...
#pipiens1-1             ... ... ... ... ... ... ... ... ... ... ... ... ... ... ... ... ... ... ... ... ... ... ... ... ... ...
#pipiens1-2             ... ... ... ... ... ... ... ... ... ... ... ... ... ... ... ... ... ... ... ... ... ... ... ... ... ...
#pipiens1-3             ... ... ... ... ... ... ... ... ... ... ... ... ... ... ... ... ... ... ... ... ... ... ... ... ... ...
#pipiens2-1             ... ... ... ... ... ... ... ... ... ... ... ... ... ... ... ... ... ... ... ... ... ... ... ... ... ...
#pipiens2-2             ... ... ... ... ... ... ... ... ... ... ... ... ... ... ... ... ... ... ... ... ... ... ... ... ... ...
#pipiens2-3             ... ... ... ... ... ... ... ... ... ... ... ... ... ... ... ... ... ... ... ... ... ... ... ... ... ...
#pipiens3-1             ... ... ... ... ... ..A ... ... ... ... ... ... ... ... ... ... ... ... ... ... ... ... ... ... ... ...
#pipiens3-2             ... ... ... ... ... ..A ... ... ... ... ... ... ... ... ... ... ... ... ... ... ... ... ... ... ... ...
#pipiens3-3             ... ... ... ... ... ..A ... ... ... ... ... ... ... ... ... ... ... ... ... ... ... ... ... ... ... ...
#pipiensUSA_KM355980    ... ... ... ... ... ... ... ... ... ... ... ... ... ... ... ... ... ... ... ... ... ... ... ... ... ...
#quinq_CPIJ007193       ... ... ... ... ... ... ... ... ... ... ... ... ... ... ... ..A ... ... ... ... ... ... ... ... ..T ...

[                       666 666 666 666 666 666 666 666 666 666 666 666 666 666 666 666 666 666 666 666 666 666 666 666 666 777 ]
[                       222 223 333 333 333 444 444 444 455 555 555 556 666 666 666 777 777 777 788 888 888 889 999 999 999 000 ]
[                       567 890 123 456 789 012 345 678 901 234 567 890 123 456 789 012 345 678 901 234 567 890 123 456 789 012 ]
#molestus2-1            TTC GCG AGT CAG ATC ACC TCG AAG GTG GTG CTG CCG TTG GGA GAG TCC AAG AAT GGT TCG AAT AAC AAG GAC CAG AAG
#molestus2-2            ... ... ... ... ... ... ... ... ... ... ... ... ... ... ... ... ... ... ... ... ... ... ... ... ... ...
#molestus2-3            ... ... ... ... ... ... ... ... ... ... ... ... ... ... ... ... ... ... ... ... ... ... ... ... ... ...
#molestus1-1            ... ... ... ... ... ... ... ... ... ... ... ... ... ... ... ... ... ... ... ... ... ... ... ... ... ...
#molestus1-2            ... ... ... ... ... ... ... ... ... ... ... ... ... ... ... ... ... ... ... ... ... ... ... ... ... ...
#molestus1-3            ... ... ... ... ... ... ... ... ... ... ... ... ... ... ... ... ... ... ... ... ... ... ... ... ... ...
#molestus3-1            ... ... ... ... ... ... ... ... ... ... ... ... ... ... ... ... ... ... ... ... ... ... ... ... ... ...
#molestus3-2            ... ... ... ... ... ... ... ... ... ... ... ... ... ... ... ... ... ... ... ... ... ... ... ... ... ...
```

```
#molestus3-3            ... ... ... ... ... ... ... ... ... ... ... ... ... ... ... ... ... ... ... ... ... ... ... ... ... ...

#pipiens1-1             ... ... ... ... ... ... ... ... ... ... ... ... ... ... ... ... ... ... ... ... ... ... ... ... ... ...

#pipiens1-2             ... ... ... ... ... ... ... ... ... ... ... ... ... ... ... ... ... ... ... ... ... ... ... ... ... ...

#pipiens1-3             ... ... ... ... ... ... ... ... ... ... ... ... ... ... ... ... ... ... ... ... ... ... ... ... ... ...

#pipiens2-1             ... ... ... ... ... ... ... ... ... ... ... ... ... ... ... ... ... ... ... ... ... ... ... ... ... ...

#pipiens2-2             ... ... ... ... ... ... ... ... ... ... ... ... ... ... ... ... ... ... ... ... ... ... ... ... ... ...

#pipiens2-3             ... ... ... ... ... ... ... ... ... ... ... ... ... ... ... ... ... ... ... ... ... ... ... ... ... ...

#pipiens3-1             ... ... ... ... ... ... ... ... ... ... ... ... ... ... ... ... ... ... ... ... ... ... ... ... ... ...

#pipiens3-2             ... ... ... ... ... ... ... ... ... ... ... ... ... ... ... ... ... ... ... ... ... ... ... ... ... ...

#pipiens3-3             ... ... ... ... ... ... ... ... ... ... ... ... ... ... ... ... ... ... ... ... ... ... ... ... ... ...

#pipiensUSA_KM355980    ... ... ... ... ... ... ... ... ... ... ... ... ... ... ... ... ... ... ... ... ... ... ... ... ... ...

#quinq_CPIJ007193       ... ... ... ... ... ... ... ... ... ... ... ... ... ... ... ... ... ... ... ... ... ... ... ... ... ...


[                       777 777 777 777 777 777 777 777 777 777 777 777 777 777 777 777 777 777 777 777 777 777 777 777 777 777 ]

[                       000 000 011 111 111 112 222 222 222 333 333 333 344 444 444 445 555 555 555 666 666 666 677 777 777 778 ]

[                       345 678 901 234 567 890 123 456 789 012 345 678 901 234 567 890 123 456 789 012 345 678 901 234 567 890 ]

#molestus2-1            AAC TGT CTG TAC GTG ATG CTG CGC AAG TAT CGA GGC CTG AAG AGT GCC GGC TTT GGG GTG ACG AAG ACT TCG GTC AAC

#molestus2-2            ... ... ... ... ... ... ... ... ... ... ... ... ... ... ... ... ... ... ... ... ... ... ... ... ... ...

#molestus2-3            ... ... ... ... ... ... ... ... ... ... ... ... ... ... ... ... ... ... ... ... ... ... ... ... ... ...

#molestus1-1            ... ... ... ... ... ... ... ... ... ... ... ... ... ... ... ... ... ... ... ... ... ... ... ... ... ...

#molestus1-2            ... ... ... ... ... ... ... ... ... ... ... ... ... ... ... ... ... ... ... ... ... ... ... ... ... ...

#molestus1-3            ... ... ... ... ... ... ... ... ... ... ... ... ... ... ... ... ... ... ... ... ... ... ... ... ... ...

#molestus3-1            ... ... ... ... ... ... ... ... ... ... ... ... ... ... ... ... ... ... ... ... ... ... ... ... ... ...

#molestus3-2            ... ... ... ... ... ... ... ... ... ... ... ... ... ... ... ... ... ... ... ... ... ... ... ... ... ...
```

```
#molestus3-3            ... ... ... ... ... ... ... ... ... ... ... ... ... ... ... ... ... ... ... ... ... ... ... ... ... ...
#pipiens1-1             ... ... ... ... ... ... ... ... ... ... ... ... ... ..A ... ... ... ... ... ... ... ..A ... ... ... ...
#pipiens1-2             ... ... ... ... ... ... ... ... ... ... ... ... ... ..A ... ... ... ... ... ... ... ..A ... ... ... ...
#pipiens1-3             ... ... ... ... ... ... ... ... ... ... ... ... ... ..A ... ... ... ... ... ... ... ..A ... ... ... ...
#pipiens2-1             ... ... ... ... ... ... ... ... ... ... ... ... ... ... ... ... ... ... ... ... ... ... ... ... ... ...
#pipiens2-2             ... ... ... ... ... ... ... ... ... ... ... ... ... ... ... ... ... ... ... ... ... ... ... ... ... ...
#pipiens2-3             ... ... ... ... ... ... ... ... ... ... ... ... ... ... ... ... ... ... ... ... ... ... ... ... ... ...
#pipiens3-1             ... ... ... ... ... ... ... ... ... ... ... ... ... ... ... ... ... ... ... ... ... ... ... ... ... ...
#pipiens3-2             ... ... ... ... ... ... ... ... ... ... ... ... ... ... ... ... ... ... ... ... ... ... ... ... ... ...
#pipiens3-3             ... ... ... ... ... ... ... ... ... ... ... ... ... ... ... ... ... ... ... ... ... ... ... ... ... ...
#pipiensUSA_KM355980    ... ... ... ... ... ... ... ... ... ... ... ... ... ... ... ... ... ... ... ... ... ... ... ... ... ...
#quinq_CPIJ007193       ..T ... T.. ... ... ... ... ... ... ... ... ..T ... ... ... ... ..T ... ..A ... ... ... ... A.. ... ...

[                       777 777 777 777 777 777 788 888 888 888 888 888 888 888 888 888 888 888 888 888 888 888 888 888 888 888 ]
[                       888 888 888 999 999 999 900 000 000 001 111 111 111 222 222 222 233 333 333 334 444 444 444 555 555 555 ]
[                       123 456 789 012 345 678 901 234 567 890 123 456 789 012 345 678 901 234 567 890 123 456 789 012 345 678 ]
#molestus2-1            TAC GAG CCG TAC TGC TTG GTG TTG ACC TTC CGT GAG GCG CCG AAT GAC AAC AGT GGT GAG GTG TCG CCC AGC ACG GGT
#molestus2-2            ... ... ... ... ... ... ... ... ... ... ... ... ... ... ... ... ... ... ... ... ... ... ... ... ... ...
#molestus2-3            ... ... ... ... ... ... ... ... ... ... ... ... ... ... ... ... ... ... ... ... ... ... ... ... ... ...
#molestus1-1            ... ... ... ... ... ... ... ... ... ... ... ... ... ... ... ... ... ... ... ... ... ... ... ... ... ...
#molestus1-2            ... ... ... ... ... ... ... ... ... ... ..C ... ... ... ... ... ... ... ... ... ... ... ... ... ... ...
#molestus1-3            ... ... ... ... ... ... ... ... ... ... ..C ... ... ... ... ... ... ... ... ... ... ... ... ... ... ...
#molestus3-1            ... ... ... ... ... ... ... ... ... ... ..C ... ... ... ... ... ... ... ... ... ... ... ... ... ... ...
#molestus3-2            ... ... ... ... ... ... ... ... ... ... ..C ... ... ... ... ... ... ... ... ... ... ... ... ... ... ...
```

```
#molestus3-3              ... ... ... ... ... ... ... ... ... ... ..C ... ... ... ... ... ... ... ... ... ... ... ... ... ... ...
#pipiens1-1               ... ... ... ... ... ... ... ... ... ... ..C ... ... ... ... ... ... ... ... ... ... ... ... ... ... ...
#pipiens1-2               ... ... ... ... ... ... ... ... ... ... ..C ... ... ... ... ... ... ... ... ... ... ... ... ... ... ...
#pipiens1-3               ... ... ... ... ... ... ... ... ... ... ..C ... ... ... ... ... ... ... ... ... ... ... ... ... ... ...
#pipiens2-1               ... ... ... ... ... ... ... ... ... ... ..C ... ... ... ... ... ... ..C ... ... ... ... ... ... ... ...
#pipiens2-2               ... ... ... ... ... ... ... ... ... ... ..C ... ... ... ... ... ... ..C ... ... ... ... ... ... ... ...
#pipiens2-3               ... ... ... ... ... ... ... ... ... ... ..C ... ... ... ... ... ... ..C ... ... ... ... ... ... ... ...
#pipiens3-1               ... ... ... ... ... ... ... ... ... ... ..C ... ... ... ... ... ... ..C ... ... ... ... ... ... ... ...
#pipiens3-2               ... ... ... ... ... ... ... ... ... ... ..C ... ... ... ... ... ... ..C ... ... ... ... ... ... ... ...
#pipiens3-3               ... ... ... ... ... ... ... ... ... ... ..C ... ... ... ... ... ... ..C ... ... ... ... ... ... ... ...
#pipiensUSA_KM355980      ... ... ... ... ... ... ... ... ... ... ..C ... ... ... ... ... ... ..C ... ... ... ... ... ... ... ...
#quinq_CPIJ007193         ... ... ... ... ... ... ... ... ... ... ... ... ... ... ... ... ... ..C ... ... ... ... ... .T. ... ..A

[                         888 888 888 888 888 888 888 888 888 888 888 888 888 889 999 999 999 999 999 999 999 999 999 999 999 999 ]
[                         566 666 666 667 777 777 777 888 888 888 899 999 999 990 000 000 000 111 111 111 122 222 222 223 333 333 ]
[                         901 234 567 890 123 456 789 012 345 678 901 234 567 890 123 456 789 012 345 678 901 234 567 890 123 456 ]
#molestus2-1              CGT AAC ATT TTG CTG ATC GTT TCG GCG ACT CCG GTC AAA AGT GTT TAC ACT CAA CCT AAC GAA CGT ATG AAC GAG CGG
#molestus2-2              ... ... ... ... ... ... ... ... ... ... ... ... ... ... ... ... ... ... ... ... ... ... ... ... ... ...
#molestus2-3              ... ... ... ... ... ... ... ... ... ... ... ... ... ... ... ... ... ... ... ... ... ... ... ... ... ...
#molestus1-1              ... ... ... ... ... ... ... ... ... ... ... ... ... ... ... ... ... ... ... ... ... ... ... ... ... ...
#molestus1-2              ... ... ... ... ... ... ... ... ... ... ... ... ... ... ... ... ... ... ... ... ... ... ... ... ... ...
#molestus1-3              ... ... ... ... ... ... ... ... ... ... ... ... ... ... ... ... ... ... ... ... ... ... ... ... ... ...
#molestus3-1              ... ... ... ... ... ... ... ... ... ... ... ... ... ... ... ... ... ... ... ... ... ... ... ... ... ...
#molestus3-2              ... ... ... ... ... ... ... ... ... ... ... ... ... ... ... ... ... ... ... ... ... ... ... ... ... ...
```

```
#molestus3-3              ... ... ... ... ... ... ... ... ... ... ... ... ... ... ... ... ... ... ... ... ... ... ... ... ...
#pipiens1-1               ... ... ... ... ... ... ... ... ... ... ... ... ... ... ... ... ... ... ... ... ... ... ... ... ...
#pipiens1-2               ... ... ... ... ... ... ... ... ... ... ... ... ... ... ... ... ... ... ... ... ... ... ... ... ...
#pipiens1-3               ... ... ... ... ... ... ... ... ... ... ... ... ... ... ... ... ... ... ... ... ... ... ... ... ...
#pipiens2-1               ... ... ... ... ... ... ... ... ... ... ... ... ... ... ... ... ... ... ... ... ... ... ... ... ...
#pipiens2-2               ... ... ... ... ... ... ... ... ... ... ... ... ... ... ... ... ... ... ... ... ... ... ... ... ...
#pipiens2-3               ... ... ... ... ... ... ... ... ... ... ... ... ... ... ... ... ... ... ... ... ... ... ... ... ...
#pipiens3-1               ... ... ... ... ... ... ... ... ... ... ... ... ... ... ... ... ... ... ... ... ... ... ... ... ...
#pipiens3-2               ... ... ... ... ... ... ... ... ... ... ... ... ... ... ... ... ... ... ... ... ... ... ... ... ...
#pipiens3-3               ... ... ... ... ... ... ... ... ... ... ... ... ... ... ... ... ... ... ... ... ... ... ... ... ...
#pipiensUSA_KM355980      ... ... ... ... ... ... ... ... ... ... ... ... ... ... ... ... ... ... ... ... ... ... ... ... ...
#quinq_CPIJ007193         ... ... ... ... ... ... ... ... ... ... ..T ... ... ... ..G ... ... ..G ... ... ... ... ... ... ...

[                                                                                         111 111 111 111 111 ]
[                         999 999 999 999 999 999 999 999 999 999 999 999 999 999 999 999 999 999 999 999 999 000 000 000 000 000 ]
[                         333 444 444 444 455 555 555 556 666 666 666 777 777 777 788 888 888 889 999 999 999 000 000 000 011 111 ]
[                         789 012 345 678 901 234 567 890 123 456 789 012 345 678 901 234 567 890 123 456 789 012 345 678 901 234 ]
#molestus2-1              AAG CTC AAG TTC AGC ACT CGA CAC ACT ACG AAC GGA GTG CTG AAT TAC GTC GAT GGA AAC TCG GTC GAG TCC ATT GGA
#molestus2-2              ... ... ... ... ... ... ... ... ... ... ... ... ... ... ... ... ... ... ... ... ... ... ... ... ... ...
#molestus2-3              ... ... ... ... ... ... ... ... ... ... ... ... ... ... ... ... ... ... ... ... ... ... ... ... ... ...
#molestus1-1              ... ... ... ... ... ... ... ... ... ... ... ... ... ... ... ... ... ... ... ... ... ... ... ... ... ...
#molestus1-2              ... ..T ... ... ... ... ... ... ... ... ... ... ... ... ... ... ... ... ... ... ... ... ... ... ... ...
#molestus1-3              ... ..T ... ... ... ... ... ... ... ... ... ... ... ... ... ... ... ... ... ... ... ... ... ... ... ...
#molestus3-1              ... ... ... ... ... ... ... ... ... ... ... ... ... ... ... ... ... ... ... ... ... ... ... ... ... ...
```

#molestus3-2	... ...
#molestus3-3	... ...
#pipiens1-1	... ...
#pipiens1-2	... ...
#pipiens1-3	... ...
#pipiens2-1	... ...
#pipiens2-2	... ...
#pipiens2-3	... ...
#pipiens3-1	... ...
#pipiens3-2	... ...
#pipiens3-3	... ...
#pipiensUSA_KM355980	... ...
#quinq_CPIJ007193	... ... .G. ... ...

[	111 111 111 111 111 111 111 111 111 111 111 111 111 111 111 111 111 111 111 111 111 111 111 111 111 111]
[	000 000 000 000 000 000 000 000 000 000 000 000 000 000 000 000 000 000 000 000 000 000 000 000 000 000]
[	111 112 222 222 222 333 333 333 344 444 444 445 555 555 555 666 666 666 677 777 777 778 888 888 888 999]
[	567 890 123 456 789 012 345 678 901 234 567 890 123 456 789 012 345 678 901 234 567 890 123 456 789 012]
#molestus2-1	TAT CTG CCA CAG GAC ATT CTC GGT CAG TCC ATT ATG GAA CTG TAC CAT CCG GAG GAC ATG --- CTG GAG TTT GTC ATT
#molestus2-2	... ... --- ... ...
#molestus2-3	... ... --- ... ...
#molestus1-1	... ... --- ... ...
#molestus1-2	... ... --- ... ...
#molestus1-3	... ... --- ... ...

#molestus3-1	... ... --- ... ...
#molestus3-2	... ... --- ... ...
#molestus3-3	... ... --- ... ...
#pipiens1-1	... ... --- ... ...
#pipiens1-2	... ... --- ... ...
#pipiens1-3	... ... --- ... ...
#pipiens2-1	... ... --- ... ...
#pipiens2-2	... ... --- ... ...
#pipiens2-3	... ... --- ... ...
#pipiens3-1	... ... --- ... ...
#pipiens3-2	... ... --- ... ...
#pipiens3-3	... ... --- ... ...
#pipiensUSA_KM355980	... ... --- ... ...
#quinq_CPIJ007193	... .G ... ...T ... ...T ... ... --- T.. ... ...

[	111 111 111 111 111 111 111 111 111 111 111 111 111 111 111 111 111 111 111 111 111 111 111 111 111 111]
[	000 000 011 111 111 111 111 111 111 111 111 111 111 111 111 111 111 111 111 111 111 111 111 111 111 111]
[	999 999 900 000 000 001 111 111 111 222 222 222 233 333 333 334 444 444 444 555 555 555 566 666 666 667]
[	345 678 901 234 567 890 123 456 789 012 345 678 901 234 567 890 123 456 789 012 345 678 901 234 567 890]
#molestus2-1	GGT AAT CAT CAC ATT ATG GAA GGT CCG TCC GAT CCG GAC GTG TTT GCA TTG CAC TTC AAC CAT CAA CAC CAG TTT GCG
#molestus2-2	... ...
#molestus2-3	... ...
#molestus1-1	... ...G ... ...
#molestus1-2	... ...G ... ...

```
#molestus1-3            ... ... ... ... ... ... ... ... ... ... ... ... ... ... ... ... ... ... ... ... ... ..G ... ... ... ...

#molestus3-1            ... ... ... ... ... ... ... ... ... ... ... ... ... ... ... ... ... ... ... ... ... ... ... ... ... ...

#molestus3-2            ... ... ... ... ... ... ... ... ... ... ... ... ... ... ... ... ... ... ... ... ... ..G ... ... ... ...

#molestus3-3            ... ... ... ... ... ... ... ... ... ... ... ... ... ... ... ... ... ... ... ... ... ... ... ... ... ...

#pipiens1-1             ... ... ... ... ... ... ... ... ... ... ... ... ... ... ... ... ... ... ..T ... ..C ..G ... ... ... ...

#pipiens1-2             ... ... ... ... ... ... ... ... ... ... ... ... ... ... ... ... ... ... ..T ... ... ..G ... ... ... ...

#pipiens1-3             ... ... ... ... ... ... ... ... ... ... ... ... ... ... ... ... ... ... ..T ... ..C ..G ... ... ... ...

#pipiens2-1             ... ... ... ... ... ... ... ... ... ... ... ... ... ... ... ... ... ... ..T ... ..C ..G ... ... ... ...

#pipiens2-2             ... ... ... ... ... ... ... ... ... ... ... ... ... ... ... ... ... ... ... ... ... ..G ... ... ... ...

#pipiens2-3             ... ... ... ... ... ... ... ... ... ... ... ... ... ... ... ... ... ... ..T ... ..C ..G ... ... ... ...

#pipiens3-1             ... ... ... ... ... ... ... ... ... ... ... ... ... ... ... ... ... ... ..T ... ..C ..G ... ... ... ...

#pipiens3-2             ... ... ... ... ... ... ... ... ... ... ... ... ... ... ... ... ... ... ..T ... ..C ..G ... ... ... ...

#pipiens3-3             ... ... ... ... ... ... ... ... ... ... ... ... ... ... ... ... ... ... ..T ... ..C ..G ... ... ... ...

#pipiensUSA_KM355980    ... ... ... ... ... ... ... ... ... ... ... ... ... ... ... ... ... ... ..T ... ..C ..G ... ... ... ...

#quinq_CPIJ007193       ... ... ... ... ... ... ... ... ... ..G ... ... ... ... ... ..G ... ... ..T ... ... ..G ... ..A ... ...


[                       111 111 111 111 111 111 111 111 111 111 111 111 111 111 111 111 111 111 111 111 111 111 111 111 111 111 ]

[                       111 111 111 111 111 111 111 111 111 112 222 222 222 222 222 222 222 222 222 222 222 222 222 222 222 222 ]

[                       777 777 777 888 888 888 899 999 999 990 000 000 000 111 111 111 122 222 222 222 223 333 333 333 444 444 444 ]

[                       123 456 789 012 345 678 901 234 567 890 123 456 789 012 345 678 901 234 567 890 123 456 789 012 345 678 ]

#molestus2-1            GAC GAG TTG CTG AAG GAG GCA AAA ACG ATC GAG GAG CAG ATT CTG AGG CTG CTG AAG GAG CCC GTA ACT AAA CCA TCG

#molestus2-2            ... ... ... ... ... ... ... ... ... ... ... ... ... ... ... ... ... ... ... ... ... ... ... ... ... ...

#molestus2-3            ... ... ... ... ... ... ... ... ... ... ... ... ... ... ... ... ... ... ... ... ... ... ... ... ... ...

#molestus1-1            ... ... ... ... ... ... ... ... ... ... ... ... ... ... ... ... ... ... ... ... ... ... ... ... ... ...
```

#molestus1-2 ... ...

#molestus1-3 ... ...

#molestus3-1 ... ...

#molestus3-2 ... ...

#molestus3-3 ... ...

#pipiens1-1 ... ... C.. ... ...G ..G ... ...

#pipiens1-2 ... ...G ... ...

#pipiens1-3 ... ... C.. ... ...G ..G ... ...

#pipiens2-1 ... ... C.. ... ...G ..G ... ...

#pipiens2-2 ... ...G ... ...

#pipiens2-3 ... ... C.. ... ...G ..G ... ...

#pipiens3-1 ... ... C.. ... ...G ..G ... ...

#pipiens3-2 ... ... C.. ... ...G ..G ... ...

#pipiens3-3 ... ... C.. ... ...G ..G ... ...

#pipiensUSA_KM355980 ... ... C.. ... ...A ... ...G ... ...

#quinq_CPIJ007193 ... ... C.. ... ...A ... ...G C

[111 111 111 111 111 111 111 111 111 111 111 111 111 111 111 111 111 111 111 111 111 111 111 111 111 111]

[222 222 222 222 222 222 222 222 222 222 222 222 222 222 222 222 222 333 333 333 333 333 333 333 333 333]

[455 555 555 556 666 666 666 777 777 777 788 888 888 889 999 999 999 000 000 000 011 111 111 112 222 222]

[901 234 567 890 123 456 789 012 345 678 901 234 567 890 123 456 789 012 345 678 901 234 567 890 123 456]

#molestus2-1 GAT ACG GTC AAG CAG GAA GTC TCC AAG CGT TGC AAA GCG CTG GCT TCG TTT ATG GAG GAG CTT ATG GAC GAG GTT ACG

#molestus2-2 ... ...

#molestus2-3 ... ...

```
#molestus1-1            ... ... ... ... ... ... ... ... ... ... ... ... ... ... ... ... ... ... ... ... ... ... ... ... ... ...

#molestus1-2            ... ... ... ... ... ... ... ... ... ... ... ... ... ... ... ... ... ... ... ... ... ... ... ... ... ...

#molestus1-3            ... ... ... ... ... ... ... ... ... ... ... ... ... ... ... ... ... ... ... ... ... ... ... ... ... ...

#molestus3-1            ... ... ... ... ... ... ... ... ... ... ... ... ... ... ... ... ... ... ... ... ... ... ... ... ... ...

#molestus3-2            ... ... ... ... ... ... ... ... ... ... ... ... ... ... ... ... ... ... ... ... ... ... ... ... ... ...

#molestus3-3            ... ... ... ... ... ... ... ... ... ... ... ... ... ... ... ... ... ... ... ... ... ... ... ... ... ...

#pipiens1-1             ... ... ... ... ... ... ... ... ... ... ... ... ... ... ... ... ... ... ... ... ... ... ... ... ... ...

#pipiens1-2             ... ... ... ... ... ... ... ... ... ... ... ... ... ... ... ... ... ... ... ... ... ... ... ... ... ...

#pipiens1-3             ... ... ... ... ... ... ... ... ... ... ... ... ... ... ... ... ... ... ... ... ... ... ... ... ... ...

#pipiens2-1             ... ... ... ... ... ... ... ... ... ... ... ... ... ... ... ... ... ... ... ... ... ... ... ... ... ...

#pipiens2-2             ... ... ... ... ... ... ... ... ... ... ... ... ... ... ... ... ... ... ... ... ... ... ... ... ... ...

#pipiens2-3             ... ... ... ... ... ... ... ... ... ... ... ... ... ... ... ... ... ... ... ... ... ... ... ... ... ...

#pipiens3-1             ... ... ... ... ... ... ... ... ... ... ... ... ... ... ... ... ... ... ... ... ... ... ... ... ... ...

#pipiens3-2             ... ... ... ... ... ... ... ... ... ... ... ... ... ... ... ... ... ... ... ... ... ... ... ... ... ...

#pipiens3-3             ... ... ... ... ... ... ... ... ... ... ... ... ... ... ... ... ... ... ... ... ... ... ... ... ... ...

#pipiensUSA_KM355980    ... ... ... ... ... ... ... ... ... ... ... ... ... ... ... ... ... ... ..A ... ... ... ... ... ... ...

#quinq_CPIJ007193       ... ... ... ... ... ... ... ... ... ... ... ... ... ... ... ... ..C ... ... ... ... ... ... ... ... ...


[                       111 111 111 111 111 111 111 111 111 111 111 111 111 111 111 111 111 111 111 111 111 111 111 111 111 111 ]

[                       333 333 333 333 333 333 333 333 333 333 333 333 333 333 333 333 333 333 333 333 333 333 333 333 344 444 ]

[                       222 333 333 333 344 444 444 445 555 555 555 666 666 666 677 777 777 778 888 888 888 999 999 999 900 000 ]

[                       789 012 345 678 901 234 567 890 123 456 789 012 345 678 901 234 567 890 123 456 789 012 345 678 901 234 ]

#molestus2-1            CAG CCT GAG CTC AAG CTG AAC CTG CTG AAC GAG TCG GAT TTT ACC TTC TCG GAG CGG GAT TCA GTC ATG CTT GGC GAG

#molestus2-2            ... ... ... ... ... ... ... ... ... ... ... ... ... ... ... ... ... ... ... ... ... ... ... ... ... ...
```

#molestus2-3	... ...
#molestus1-1	... ...
#molestus1-2	... ...
#molestus1-3	... ...
#molestus3-1	... ...
#molestus3-2	... ...
#molestus3-3	... ...
#pipiens1-1	... ..C ... ... T.. ... ...
#pipiens1-2	... ..C ... ... T.. ... ...
#pipiens1-3	... ..C ... ... T.. ... ...
#pipiens2-1	... ..C ... ... T.. ... ...
#pipiens2-2	... ..C ... ... T.. ... ...
#pipiens2-3	... ..C ... ... T.. ... ...
#pipiens3-1	... ..C ... ... T.. ... ...
#pipiens3-2	... ..C ... ... T.. ... ...
#pipiens3-3	... ..C ... ... T.. ... ...
#pipiensUSA_KM355980	... ..C ... ...G ... ...
#quinq_CPIJ007193	... ..C ... ..T ... ...G ... ...

[	111 111 111 111 111 111 111 111 111 111 111 111 111 111 111 111 111 111 111 111 111 111 111 111 111 111	]
[	444 444 444 444 444 444 444 444 444 444 444 444 444 444 444 444 444 444 444 444 444 444 444 444 444 444	]
[	000 001 111 111 111 222 222 222 233 333 333 334 444 444 444 555 555 555 566 666 666 667 777 777 777 888	]
[	567 890 123 456 789 012 345 678 901 234 567 890 123 456 789 012 345 678 901 234 567 890 123 456 789 012	]
#molestus2-1	ATA TCT CCG CAT CAC GAT TAT TTC AAT AGT AAG AGC TCC TCG GAG ACC CCA CCG AGT TAC AAT CAA CTC AAT TAC AAC	

```
#molestus2-2          ... ... ... ... ... ... ... ... ... ... ... ... ... ... ... ... ... ... ... ... ... ... ... ... ... ...
#molestus2-3          ... ... ... ... ... ... ... ... ... ... ... ... ... ... ... ... ... ... ... ... ... ... ... ... ... ...
#molestus1-1          ... ... ... ... ... ... ... ... ... ... ... ... ... ... ... ... ... ... ... ... ... ... ... ... ... ...
#molestus1-2          ... ... ... ... ... ... ... ... ... ... ... ... ... ... ... ... ... ... ... ... ... ... ... ... ... ...
#molestus1-3          ... ... ... ... ... ... ... ... ... ... ... ... ... ... ... ... ... ... ... ... ... ... ... ... ... ...
#molestus3-1          ... ... ... ... ... ... ... ... ... ... ... ... ... ... ... ... ... ... ... ... ... ... ... ... ... ...
#molestus3-2          ... ... ... ... ... ... ... ... ... ... ... ... ... ... ... ... ... ... ... ... ... ... ... ... ... ...
#molestus3-3          ... ... ... ... ... ... ... ... ... ... ... ... ... ... ... ... ... ... ... ... ... ... ... ... ... ...
#pipiens1-1           ... ... ... ... ... ... ... ... ... ... ... ... ... ... ... ... ..G ... ... ... ... ... ... ... ... ...
#pipiens1-2           ... ... ... ... ... ... ... ... ... ... ... ... ... ... ... ... ... ... ... ... ... ... ... ... ... ...
#pipiens1-3           ... ... ... ... ... ... ... ... ... ... ... ... ... ... ... ... ..G ... ... ... ... ... ... ... ... ...
#pipiens2-1           ... ... ... ... ... ... ... ... ... ... ... ... ... ... ... ... ..G ... ... ... ... ... ... ... ... ...
#pipiens2-2           ... ... ... ... ... ... ... ... ... ... ... ... ... ... ... ... ... ... ... ... ... ... ... ... ... ...
#pipiens2-3           ... ... ... ... ... ... ... ... ... ... ... ... ... ... ... ... ..G ... ... ... ... ... ... ... ... ...
#pipiens3-1           ... ... ... ... ... ... ... ... ... ... ... ... ... ... ... ... ..G ... ... ... ... ... ... ... ... ...
#pipiens3-2           ... ... ... ... ... ... ... ... ... ... ... ... ... ... ... ... ..G ... ... ... ... ... ... ... ... ...
#pipiens3-3           ... ... ... ... ... ... ... ... ... ... ... ... ... ... ... ... ..G ... ... ... ... ... ... ... ... ...
#pipiensUSA_KM355980  ... ... ... ... ... ... ... ... ... ... ... ... ... ... ... ... ..G ... ... ... ... ... ... ... ... ...
#quinq_CPIJ007193     ... ... ... ... ... ... ... ... ... ... ... ... ... ... ... ... ..G ... ... ... ... ... ... ... ... ...

[                     111 111 111 111 111 111 111 111 111 111 111 111 111 111 111 111 111 111 111 111 111 111 111 111 111 111 ]
[                     444 444 444 444 444 445 555 555 555 555 555 555 555 555 555 555 555 555 555 555 555 555 555 555 555 555 ]
[                     888 888 899 999 999 990 000 000 000 111 111 111 122 222 222 223 333 333 333 444 444 444 455 555 555 556 ]
[                     345 678 901 234 567 890 123 456 789 012 345 678 901 234 567 890 123 456 789 012 345 678 901 234 567 890 ]
```

```
#molestus2-1            GAC AAC TTG CAG CGG TTC TTC GAC TCT CGT CCG GTC ATG AAC ATT GAA GAA TCG CTG AAG AAT GAC TCG TCC GGT GGG

#molestus2-2            ... ... ... ... ... ... ... ... ... ... ... ... ... ... ... ... ... ... ... ... ... ... ... ... ... ...

#molestus2-3            ... ... ... ... ... ... ... ... ... ... ... ... ... ... ... ... ... ... ... ... ... ... ... ... ... ...

#molestus1-1            ... ... ... ... ... ... ... ... ... ... ... ... ... ... ... ... ... ... ... ... ... ... ... ... ... ...

#molestus1-2            ... ... ... ... ... ... ... ... ... ... ... ... ... ... ... ... ... ... ... ... ... ... ... ... ... ...

#molestus1-3            ... ... ... ... ... ... ... ... ... ... ... ... ... ... ... ... ... ... ... ... ... ... ... ... ... ...

#molestus3-1            ... ... ... ... ... ... ... ... ... ... ... ... ... ... ... ... ... ... ... ... ... ... ... ... ... ...

#molestus3-2            ... ... ... ... ... ... ... ... ... ... ... ... ... ... ... ... ... ... ... ... ... ... ... ... ... ...

#molestus3-3            ... ... ... ... ... ... ... ... ... ... ... ... ... ... ... ... ... ... ... ... ... ... ... ... ... ...

#pipiens1-1             ... ... ... ... ... ... ... ... ... ... ... ... ... ... ... ... ... ... ... ... ... ... ... ... ... ...

#pipiens1-2             ... ... ... ... ... ... ... ... ... ... ... ... ... ... ... ... ... ... ... ... ... ... ... ... ... ...

#pipiens1-3             ... ... ... ... ... ... ... ... ... ... ... ... ... ... ... ... ... ... ... ... ... ... ... ... ... ...

#pipiens2-1             ... ... ... ... ... ... ... ... ... ... ... ... ... ... ... ... ... ... ... ... ... ... ... ... ... ...

#pipiens2-2             ... ... ... ... ... ... ... ... ... ... ... ... ... ... ... ... ... ... ... ... ... ... ... ... ... ...

#pipiens2-3             ... ... ... ... ... ... ... ... ... ... ... ... ... ... ... ... ... ... ... ... ... ... ... ... ... ...

#pipiens3-1             ... ... ... ... ... ... ... ... ... ... ... ... ... ... ... ... ... ... ... ... ... ... ... ... ... ...

#pipiens3-2             ... ... ... ... ... ... ... ... ... ... ... ... ... ... ... ... ... ... ... ... ... ... ... ... ... ...

#pipiens3-3             ... ... ... ... ... ... ... ... ... ... ... ... ... ... ... ... ... ... ... ... ... ... ... ... ... ...

#pipiensUSA_KM355980    ... ... ... ... ... ... ... ... ... ... ... ... ... ... ... ... ... ... ... ... ... ..A ... ... ... ...

#quinq_CPIJ007193       ... ... ... ... ... ... ... ... ... ... ... ... ... ... ... ... ..G ... ... ... ... ... ... ... ... ...


[                       111 111 111 111 111 111 111 111 111 111 111 111 111 111 111 111 111 111 111 111 111 111 111 111 111 111 ]

[                       555 555 555 555 555 555 555 555 555 555 555 555 555 666 666 666 666 666 666 666 666 666 666 666 666 666 ]

[                       666 666 666 777 777 777 788 888 888 889 999 999 999 000 000 000 011 111 111 112 222 222 222 333 333 333 ]
```

```
[                         123 456 789 012 345 678 901 234 567 890 123 456 789 012 345 678 901 234 567 890 123 456 789 012 345 678 ]

#molestus2-1              ACA AAC ACC GAG ACC CAG GAT GAC CGG ACG AAC GTC AGC CCG AAC CAG CGG TTC AGC GCA AGT GGA GGT GGC GGC AGT

#molestus2-2              ... ... ... ... ... ... ... ... ... ... ... ... ... ... ... ... ... ... ... ... ... ... ... ... ... ...

#molestus2-3              ... ... ... ... ... ... ... ... ... ... ... ... ... ... ... ... ... ... ... ... ... ... ... ... ... ...

#molestus1-1              ... ... ... ... ... ... ... ... ... ... ... ... T.. ... ... ... ... ... ... ... ... ... ... ... ... ...

#molestus1-2              ... ... ... ... ... ... ... ... ... ... ... ... T.. ... ... ... ... ... ... ... ... ... ... ... ... ...

#molestus1-3              ... ... ... ... ... ... ... ... ... ... ... ... T.. ... ... ... ... ... ... ... ... ... ... ... ... ...

#molestus3-1              ... ... ... ... ... ... ... ... ... ... ... ... ... ... ... ... ... ... ... ... ... ... ... ... ... ...

#molestus3-2              ... ... ... ... ... ... ... ... ... ... ... ... ... ... ... ... ... ... ... ... ... ... ... ... ... ...

#molestus3-3              ... ... ... ... ... ... ... ... ... ... ... ... ... ... ... ... ... ... ... ... ... ... ... ... ... ...

#pipiens1-1               ... ... ... ... ... ... ... ... ... ... ... ... ... ... ... ... ... ... ... ... ... ... ... ... ... ...

#pipiens1-2               ... ... ... ... ... ... ... ... ... ... ... ... ... ... ... ... ... ... ... ... ... ... ... ... ... ...

#pipiens1-3               ... ... ... ... ... ... ... ... ... ... ... ... ... ... ... ... ... ... ... ... ... ... ... ... ... ...

#pipiens2-1               ... ... ... ... ... ... ... ... ... ... ... ... ... ... ... ... ... ... ... ... ... ... ... ... ... ...

#pipiens2-2               ... ... ... ... ... ... ... ... ... ... ... ... ... ... ... ... ... ... ... ... ... ... ... ... ... ...

#pipiens2-3               ... ... ... ... ... ... ... ... ... ... ... ... ... ... ... ... ... ... ... ... ... ... ... ... ... ...

#pipiens3-1               ... ... ... ... ... ... ... ... ... ... ... ... ... ... ... ... ... ... ... ... ... ... ... ... ... ...

#pipiens3-2               ... ... ... ... ... ... ... ... ... ... ... ... ... ... ... ... ... ... ... ... ... ... ... ... ... ...

#pipiens3-3               ... ... ... ... ... ... ... ... ... ... ... ... ... ... ... ... ... ... ... ... ... ... ... ... ... ...

#pipiensUSA_KM355980      ... ... ... ... ... ... ... ... ... ... ... ... ... ... ... ... ... ... ... ... ... ... ... ... ... ...

#quinq_CPIJ007193         ... ... ... ... .T. ... ... ... ... ... ... ... ... ... ... ... ... ..T ... ... ... ... ... ... ... ...


[                         111 111 111 111 111 111 111 111 111 111 111 111 111 111 111 111 111 111 111 111 111 111 111 111 111 111 ]

[                         666 666 666 666 666 666 666 666 666 666 666 666 666 666 666 666 666 666 666 666 677 777 777 777 777 777 ]
```

```
[                        344 444 444 445 555 555 555 666 666 666 677 777 777 778 888 888 888 999 999 999 900 000 000 001 111 111 ]
[                        901 234 567 890 123 456 789 012 345 678 901 234 567 890 123 456 789 012 345 678 901 234 567 890 123 456 ]
#molestus2-1             GGT GAC AGT GCG GGA AAT TTC AGC TCC GAA AGT AAC GCT CAA ATG GAC AGC ACA ACC AAC ACG ACC AGC AAC ACC GGC
#molestus2-2             ... ... ... ... ... ... ... ... ... ... ... ... ... ... ... ... ... ... ... ... ... ... ... ... ... ...
#molestus2-3             ... ... ... ... ... ... ... ... ... ... ... ... ... ... ... ... ... ... ... ... ... ... ... ... ... ...
#molestus1-1             ... ... ... ... ... ... ... ... ... ... ... ... ... ... ... ... ... ... ... ... ... ... ... ... ... ...
#molestus1-2             ... ... ... ... ... ... ... ... ... ... ... ... ... ... ... ... ... ... ... ... ... ... ... ... ... ...
#molestus1-3             ... ... ... ... ... ... ... ... ... ... ... ... ... ... ... ... ... ... ... ... ... ... ... ... ... ...
#molestus3-1             ... ... ... ... ... ... ... ... ... ... ... ... ... ... ... ... ... ... ... ... ... ... ... ... ... ...
#molestus3-2             ... ... ... ... ... ... ... ... ... ... ... ... ... ... ... ... ... ... ... ... ... ... ... ... ... ...
#molestus3-3             ... ... ... ... ... ... ... ... ... ... ... ... ... ... ... ... ... ... ... ... ... ... ... ... ... ...
#pipiens1-1              ... ... ... ... ... ... ... ... ... ... ... ... ... ... ... ... ... ... ... ... ... ... ... ... ... ...
#pipiens1-2              ... ... ... ... ... ... ... ... ... ... ... ... ... ... ... ... ... ... ... ... ... ... ... ... ... ...
#pipiens1-3              ... ... ... ... ... ... ... ... ... ... ... ... ... ... ... ... ... ... ... ... ... ... ... ... ... ...
#pipiens2-1              ... ... ... ... ... ... ... ... ... ... ... ... ... ... ... ... ... ... ... ... ... ... ... ... ... ...
#pipiens2-2              ... ... ... ... ... ... ... ... ... ... ... ... ... ... ... ... ... ... ... ... ... ... ... ... ... ...
#pipiens2-3              ... ... ... ... ... ... ... ... ... ... ... ... ... ... ... ... ... ... ... ... ... ... ... ... ... ...
#pipiens3-1              ... ... ... ... ..G ... ... ... ... ... ... ... ... ... ... ... ... ... ... ... ... ... ... ... ... ...
#pipiens3-2              ... ... ... ... ..G ... ... ... ... ... ... ... ... ... ... ... ... ... ... ... ... ... ... ... ... ...
#pipiens3-3              ... ... ... ... ..G ... ... ... ... ... ... ... ... ... ... ... ... ... ... ... ... ... ... ... ... ...
#pipiensUSA_KM355980     ... ... ... ... ... ... ... ... ... ..G ... ... ... ... ... ... ... ... ... ... ... ... ... ... ... ...
#quinq_CPIJ007193        ... .G. ... ... ... ... ... ... ... ... ..C ... ... ... ... ... ... ... ... ... ... ... ... ... ... ...

[                        111 111 111 111 111 111 111 111 111 111 111 111 111 111 111 111 111 111 111 111 111 111 111 111 111 111 ]
```

```
[                         777 777 777 777 777 777 777 777 777 777 777 777 777 777 777 777 777 777 777 777 777 777 777 777 777 777 ]
[                         111 222 222 222 233 333 333 334 444 444 444 555 555 555 566 666 666 667 777 777 777 888 888 888 899 999 ]
[                         789 012 345 678 901 234 567 890 123 456 789 012 345 678 901 234 567 890 123 456 789 012 345 678 901 234 ]
#molestus2-1              ACC TCG TCC GGC AGC GTA CAA CCG CCG ACG TTG ACG GAA GAG TTG CTT TGC CTG CAC AAC GAA GAC ATG CAG AAG GTG
#molestus2-2              ... ... ... ... ... ... ... ... ... ... ... ... ... ... ... ... ... ... ... ... ... ... ... ... ... ...
#molestus2-3              ... ... ... ... ... ... ... ... ... ... ... ... ... ... ... ... ... ... ... ... ... ... ... ... ... ...
#molestus1-1              ... ... ... C.. ... ... ... ... ... ... ... ... ... ... ... ... ... ... ... ... ... ... ... ... ... ...
#molestus1-2              ... ... ... C.. ... ... ... ... ... ... ... ... ... ... ... ... ... ... ... ... ... ... ... ... ... ...
#molestus1-3              ... ... ... C.. ... ... ... ... ... ... ... ... ... ... ... ... ... ... ... ... ... ... ... ... ... ...
#molestus3-1              ... ... ... ... ... ... ... ... ... ... ... ... ... ... ... ... ... ... ... ... ... ... ... ... ... ...
#molestus3-2              ... ... ... ... ... ... ... ... ... ... ... ... ... ... ... ... ... ... ... ... ... ... ... ... ... ...
#molestus3-3              ... ... ... ... ... ... ... ... ... ... ... ... ... ... ... ... ... ... ... ... ... ... ... ... ... ...
#pipiens1-1               ... ... ... ... ... ... ... ... ... ... ... ... ... ... ... ... ... ... ... ..T ... ... ... ... ... ...
#pipiens1-2               ... ... ... ... ... ... ... ... ... ... ... ... ... ... ... ... ... ... ... ..T ... ... ... ... ... ...
#pipiens1-3               ... ... ... ... ... ... ... ... ... ... ... ... ... ... ... ... ... ... ... ..T ... ... ... ... ... ...
#pipiens2-1               ... ... ... ... ... ... ... ... ... ... ... ... ... ... ... ... ... ... ... ..T ... ... ... ... ... ...
#pipiens2-2               ... ... ... ... ... ... ... ... ... ... ... ... ... ... ... ... ... ... ... ..T ... ... ... ... ... ...
#pipiens2-3               ... ... ... ... ... ... ... ... ... ... ... ... ... ... ... ... ... ... ... ..T ... ... ... ... ... ...
#pipiens3-1               ... ... ... ... ... ... ... ... ... ... ... ... ... ... ... ... ... ... ... ... ... ... ... ... ... ...
#pipiens3-2               ... ... ... ... ... ... ... ... ... ... ... ... ... ... ... ... ... ... ... ... ... ... ... ... ... ...
#pipiens3-3               ... ... ... ... ... ... ... ... ... ... ... ... ... ... ... ... ... ... ... ... ... ... ... ... ... ...
#pipiensUSA_KM355980      ... ... ... ... ... ... ... ... ... ... ... ... ... ... ... ... ... ... ... ... ... ... ... ... ... ...
#quinq_CPIJ007193         ... ... ... ... ... ... ... ... ... ... ... ... ... ... ... ... ... .A. ... ... ... ... ... ... ... ...
```

```
[                        111 111 111 111 111 111 111 111 111 111 111 111 111 111 111 111 111 111 111 111 111 111 111 111 111 111 ]
[                        777 778 888 888 888 888 888 888 888 888 888 888 888 888 888 888 888 888 888 888 888 888 888 888 888 888 ]
[                        999 990 000 000 000 111 111 111 122 222 222 223 333 333 333 444 444 444 455 555 555 556 666 666 666 777 ]
[                        567 890 123 456 789 012 345 678 901 234 567 890 123 456 789 012 345 678 901 234 567 890 123 456 789 012 ]
#molestus2-1             ATG CTG AAG AGG CAT CGT GAG GCA CGA ACC ACG AGC CGA GGA GCG GAC AAA AGC CGA AAG GGA CCG CCG GAC AAA GTG
#molestus2-2             ... ... ... ... ... ... ... ... ... ... ... ... ... ... ... ... ... ... ... ... ... ... ... ... ... ...
#molestus2-3             ... ... ... ... ... ... ... ... ... ... ... ... ... ... ... ... ... ... ... ... ... ... ... ... ... ...
#molestus1-1             ... ... ... ... ... ..C ... ... ... ... ... ... ... ... ... ... ... ... ... ... ... ... ... ... ... ...
#molestus1-2             ... ... ... ... ... ..C ... ... ... ... ... ... ... ... ... ... ... ... ... ... ... ... ... ... ... ...
#molestus1-3             ... ... ... ... ... ..C ... ... ... ... ... ... ... ... ... ... ... ... ... ... ... ... ... ... ... ...
#molestus3-1             ... ... ... ... ... ..C ... ... ... ... ... ... ... ... ... ... ... ... ... ... ... ... ... ... ... ...
#molestus3-2             ... ... ... ... ... ..C ... ... ... ... ... ... ... ... ... ... ... ... ... ... ... ... ... ... ... ...
#molestus3-3             ... ... ... ... ... ..C ... ... ... ... ... ... ... ... ... ... ... ... ... ... ... ... ... ... ... ...
#pipiens1-1              ... ... ... ... ... ..C ... ... ... ... ... ... ... ... ... ... ... ... ... ... ... ... ... ... ... ...
#pipiens1-2              ... ... ... ... ... ..C ... ... ... ... ... ... ... ... ... ... ... ... ... ... ... ... ... ... ... ...
#pipiens1-3              ... ... ... ... ... ..C ... ... ... ... ... ... ... ... ... ... ... ... ... ... ... ... ... ... ... ...
#pipiens2-1              ... ... ... ... ... ..C ... ... ... ... ... ... ... ... ... ... ... ... ... ... ... ... ... ... ... ...
#pipiens2-2              ... ... ... ... ... ..C ... ... ... ... ... ... ... ... ... ... ... ... ... ... ... ... ... ... ... ...
#pipiens2-3              ... ... ... ... ... ..C ... ... ... ... ... ... ... ... ... ... ... ... ... ... ... ... ... ... ... ...
#pipiens3-1              ... ... ... ... ... ..C ... ... ... ... ... ... ..G ... ... ... ... ... ... ... ... ... ... ... ... ...
#pipiens3-2              ... ... ... ... ... ..C ... ... ... ... ... ... ..G ... ... ... ... ... ... ... ... ... ... ... ... ...
#pipiens3-3              ... ... ... ... ... ..C ... ... ... ... ... ... ..G ... ... ... ... ... ... ... ... ... ... ... ... ...
#pipiensUSA_KM355980     ... ... ... ... ... ..C ... ... ... ... ... ... ..G ... ... ... ... ... ... ... ... ... ... ... ... ...
#quinq_CPIJ007193        ... ... ... ... ... ..C ... ... ... ... ... ... A.. ... ... ... ... ... ... ... ... ... ... ... ... ...
```

```
[                        111 111 111 111 111 111 111 111 111 111 111 111 111 111 111 111 111 111 111 111 111 111 111 111 111 111 ]
[                        888 888 888 888 888 888 888 888 888 999 999 999 999 999 999 999 999 999 999 999 999 999 999 999 999 999 ]
[                        777 777 788 888 888 889 999 999 999 000 000 000 011 111 111 112 222 222 222 333 333 333 344 444 444 445 ]
[                        345 678 901 234 567 890 123 456 789 012 345 678 901 234 567 890 123 456 789 012 345 678 901 234 567 890 ]
#molestus2-1             TAC ACG AAC ATC GCA ACG CAT GGC GTC AAG CGG GGA TCA TCG AAC TCG TGG GAG GAA GAC ATG CAC AAA ACT GCA AAG
#molestus2-2             ... ... ... ... ... ... ... ... ... ... ... ... ... ... ... ... ... ... ... ... ... ... ... ... ... ...
#molestus2-3             ... ... ... ... ... ... ... ... ... ... ... ... ... ... ... ... ... ... ... ... ... ... ... ... ... ...
#molestus1-1             ... ... ... ... ... ... ... ... ... ... ... ... ... ... ... ... ... ... ... ... ... ... ... ... ... ...
#molestus1-2             ... ... ... ... ... ... ... ... ... ... ... ... ... ... ... ... ... ... ... ... ... ... ... ... ... ...
#molestus1-3             ... ... ... ... ... ... ... ... ... ... ... ... ... ... ... ... ... ... ... ... ... ... ... ... ... ...
#molestus3-1             ... ... ... ... ... ... ... ... ... ... ... ... ... ... ... ... ... ... ... ... ... ... ... ... ... ...
#molestus3-2             ... ... ... ... ... ... ... ... ... ... ... ... ... ... ... ... ... ... ... ... ... ... ... ... ... ...
#molestus3-3             ... ... ... ... ... ... ... ... ... ... ... ... ... ... ... ... ... ... ... ... ... ... ... ... ... ...
#pipiens1-1              ... ... ... ... ... ... ... ... ... ... ... ... ... ... ... ... ... ... ... ..T ... ... ... ... ..C ...
#pipiens1-2              ... ... ... ... ... ... ... ... ... ... ... ... ... ... ... ... ... ... ... ..T ... ... ... ... ..C ...
#pipiens1-3              ... ... ... ... ... ... ... ... ... ... ... ... ... ... ... ... ... ... ... ..T ... ... ... ... ..C ...
#pipiens2-1              ... ... ... ... ... ... ... ... ... ... ... ... ... ... ... ... ... ... ... ..T ... ... ... ... ..C ...
#pipiens2-2              ... ... ... ... ... ... ... ... ... ... ... ... ... ... ... ... ... ... ... ..T ... ... ... ... ..C ...
#pipiens2-3              ... ... ... ... ... ... ... ... ... ... ... ... ... ... ... ... ... ... ... ..T ... ... ... ... ..C ...
#pipiens3-1              ... ... ... ... ... ... ... ... ... ... ... ... ... ... ... ... ... ... ... ..T ... ... ... ... ..C ...
#pipiens3-2              ... ... ... ... ... ... ... ... ... ... ... ... ... ... ... ... ... ... ... ..T ... ... ... ... ..C ...
#pipiens3-3              ... ... ... ... ... ... ... ... ... ... ... ... ... ... ... ... ... ... ... ..T ... ... ... ... ..C ...
#pipiensUSA_KM355980     ... ... ... ... ... ... ... ... ... ... ... ... ... ... ... ... ... ... ... ... ... ... ... ... ..C ...
```

```
#quinq_CPIJ007193        ... ... ... ... ... ... ... ... ... ... ... ... ... ... C.. ... ... ... ... ... ... ... ... ... ... ...


[                        111 111 111 111 111 111 111 111 111 111 111 111 111 111 111 111 122 222 222 222 222 222 222 222 222 222 ]
[                        999 999 999 999 999 999 999 999 999 999 999 999 999 999 999 999 900 000 000 000 000 000 000 000 000 000 ]
[                        555 555 555 666 666 666 677 777 777 778 888 888 888 999 999 999 900 000 000 001 111 111 111 222 222 222 ]
[                        123 456 789 012 345 678 901 234 567 890 123 456 789 012 345 678 901 234 567 890 123 456 789 012 345 678 ]
#molestus2-1             CAT CAG CAC AAT TCC AAC CCC ATG CGC GAA TAT CAA CCA CAA TCC TCT TCA GCG CAA CAC ACG GTG CTG CCC AAA CCC
#molestus2-2             ... ... ... ... ... ... ... ... ... ... ... ... ... ... ... ... ... ... ... ... ... ... ... ... ... ...
#molestus2-3             ... ... ... ... ... ... ... ... ... ... ... ... ... ... ... ... ... ... ... ... ... ... ... ... ... ...
#molestus1-1             ..C ... ... ... ... ... ... ... ... ..C ... ... ... ... ... ... ... ... ... ... ... ... ... ... ... ...
#molestus1-2             ..C ... ... ... ... ... ... ... ... ..C ... ... ... ... ... ... ... ... ... ... ... ... ... ... ... ...
#molestus1-3             ..C ... ... ... ... ... ... ... ... ..C ... ... ... ... ... ... ... ... ... ... ... ... ... ... ... ...
#molestus3-1             ..C ... ... ... ... ... ... ... ... ..C ... ... ... ... ... ... ... ... ... ... ... ... ... ... ... ...
#molestus3-2             ..C ... ... ... ... ... ... ... ... ..C ... ... ... ... ... ... ... ... ... ... ... ... ... ... ... ...
#molestus3-3             ..C ... ... ... ... ... ... ... ... ..C ... ... ... ... ... ... ... ... ... ... ... ... ... ... ... ...
#pipiens1-1              ..C ... ... ... ... ... ... ... ... ... ... ... ... ... ... ... ... ... ... ... ... ... ... ... ... ...
#pipiens1-2              ..C ... ... ... ... ... ... ... ... ... ... ... ... ... ... ... ... ... ... ... ... ... ... ... ... ...
#pipiens1-3              ..C ... ... ... ... ... ... ... ... ... ... ... ... ... ... ... ... ... ... ... ... ... ... ... ... ...
#pipiens2-1              ..C ... ... ... ... ... ... ... ... ... ... ... ... ... ... ... ... ... ... ... ... ... ... ... ... ...
#pipiens2-2              ..C ... ... ... ... ... ... ... ... ... ... ... ... ... ... ... ... ... ... ... ... ... ... ... ... ...
#pipiens2-3              ..C ... ... ... ... ... ... ... ... ... ... ... ... ... ... ... ... ... ... ... ... ... ... ... ... ...
#pipiens3-1              ..C ... ... ... ... ... ... ... ... ... ... ... ... ... ... ... ... ... ... ... ... ... ... ... ... ...
#pipiens3-2              ..C ... ... ... ... ... ... ... ... ... ... ... ... ... ... ... ... ... ... ... ... ... ... ... ... ...
#pipiens3-3              ..C ... ... ... ... ... ... ... ... ... ... ... ... ... ... ... ... ... ... ... ... ... ... ... ... ...
```

```
#pipiensUSA_KM355980    ..C ... ... ... ... ... ... ... ... ... ... ... ... ... ... ... ... ... ... ... ... ... ... ... ... ...

#quinq_CPIJ007193       ..C ... ... ... ... ... ... ... ... ..C ... ... ... ... ... ..A ... ... ... ... ... ... ... ... ... ...


[                       222 222 222 222 222 222 222 222 222 222 222 222 222 222 222 222 222 222 222 222 222 222 222 222 222 222 ]

[                       000 000 000 000 000 000 000 000 000 000 000 000 000 000 000 000 000 000 000 000 000 000 000 001 111 111 ]

[                       233 333 333 334 444 444 444 555 555 555 566 666 666 667 777 777 777 888 888 888 899 999 999 990 000 000 ]

[                       901 234 567 890 123 456 789 012 345 678 901 234 567 890 123 456 789 012 345 678 901 234 567 890 123 456 ]

#molestus2-1            ACC CAA CAA CGA CTT CCC GTA GTG CCG ATG GAT ACC TGC CGA GCG GTC GTG ACC ACG ACG GCC GTT ACG ATG GGC CAA

#molestus2-2            ... ... ... ... ... ... ... ... ... ... ... ... ... ... ... ... ... ... ... ... ... ... ... ... ... ...

#molestus2-3            ... ... ... ... ... ... ... ... ... ... ... ... ... ... ... ... ... ... ... ... ... ... ... ... ... ...

#molestus1-1            ... ... ... ... ... ... ... ... ... ... ... ... ... ... ... ... ... ... ... ... ... ... ... ... ... ...

#molestus1-2            ... ... ... ... ... ... ... ... ... ... ... ... ... ... ... ... ... ... ... ... ... ... ... ... ... ...

#molestus1-3            ... ... ... ... ... ... ... ... ... ... ... ... ... ... ... ... ... ... ... ... ... ... ... ... ... ...

#molestus3-1            ... ... ... ... ... ... ... ... ... ... ... ... ... ... ... ... ... ... ... ... ... ... ... ... ... ...

#molestus3-2            ... ... ... ... ... ... ... ... ... ... ... ... ... ... ... ... ... ... ... ... ... ... ... ... ... ...

#molestus3-3            ... ... ... ... ... ... ... ... ... ... ... ... ... ... ... ... ... ... ... ... ... ... ... ... ... ...

#pipiens1-1             ... ... ... ... ... ... ... ... ... ... ... ... ... ... ... ... ... ... ..T ... ... ... ... ... ... ...

#pipiens1-2             ... ... ... ... ... ... ... ... ... ... ... ... ... ... ... ... ... ... ... ... ... ... ... ... ... ...

#pipiens1-3             ... ... ... ... ... ... ... ... ... ... ... ... ... ... ... ... ... ... ..T ... ... ... ... ... ... ...

#pipiens2-1             ... ... ... ... ... ... ... ... ... ... ... ... ... ... ... ... ... ... ..T ... ... ... ... ... ... ...

#pipiens2-2             ... ... ... ... ... ... ... ... ... ... ... ... ... ... ... ... ... ... ... ... ... ... ... ... ... ...

#pipiens2-3             ... ... ... ... ... ... ... ... ... ... ... ... ... ... ... ... ... ... ..T ... ... ... ... ... ... ...

#pipiens3-1             ... ... ... ... ... ... ... ... ... ... ... ... ... ... ... ... ... ... ..T ... ... ... ... ... ... ...

#pipiens3-2             ... ... ... ... ... ... ... ... ... ... ... ... ... ... ... ... ... ... ..T ... ... ... ... ... ... ...
```

```
#pipiens3-3              ... ... ... ... ... ... ... ... ... ... ... ... ... ... ... ... ... ... ..T ... ... ... ... ... ... ...

#pipiensUSA_KM355980     ... ... ... ... ... ... ... ... ... ... ... ... ... ... ... ... ... ... ... ... ... ... ... ... ... ...

#quinq_CPIJ007193        ... ... ... ... ... ... ... ..T ... ... ... ... ... ... ..A ... ... ... ..A ... ... ..A ..C ... ... ...


[                        222 222 222 222 222 222 222 222 222 222 222 222 222 222 222 222 222 222 222 222 222 222 222 222 222 222 ]

[                        111 111 111 111 111 111 111 111 111 111 111 111 111 111 111 111 111 111 111 111 111 111 111 111 111 111 ]

[                        000 111 111 111 122 222 222 223 333 333 333 444 444 444 455 555 555 556 666 666 666 777 777 777 788 888 ]

[                        789 012 345 678 901 234 567 890 123 456 789 012 345 678 901 234 567 890 123 456 789 012 345 678 901 234 ]

#molestus2-1             CAG ACG GCT ACC GTT CCG TAC AAC CTG CCA CGT GCC GGC GAA ATG TGG CCA CCC TTC TCC GTC AGT GTC ACA ACC GTC

#molestus2-2             ... ... ... ... ... ... ... ... ... ... ... ... ... ... ... ... ... ... ... ... ... ... ... ... ... ...

#molestus2-3             ... ... ... ... ... ... ... ... ... ... ... ... ... ... ... ... ... ... ... ... ... ... ... ... ... ...

#molestus1-1             ... ... ... ... ... ... ... ... ... ... ... ... ... ... ... ... ... ... ... ... ... ... ... ... ... ...

#molestus1-2             ... ... ... ... ... ... ... ... ... ... ... ... ... ... ... ... ... ... ... ... ... ... ... ... ... ...

#molestus1-3             ... ... ... ... ... ... ... ... ... ... ... ... ... ... ... ... ... ... ... ... ... ... ... ... ... ...

#molestus3-1             ... ... ... ... ... ... ... ... ... ... ... ... ... ... ... ... ... ... ... ... ... ... ... ... ... ...

#molestus3-2             ... ... ... ... ... ... ... ... ... ... ... ... ... ... ... ... ... ... ... ... ... ... ... ... ... ...

#molestus3-3             ... ... ... ... ... ... ... ... ... ... ... ... ... ... ... ... ... ... ... ... ... ... ... ... ... ...

#pipiens1-1              ... ... ... ... ... ... ... ... ... ... ... ... ... ... ... ... ... ... ... ..T ... ..C ... ... ... ...

#pipiens1-2              ... ... ... ... ... ... ... ... ... ... ... ... ... ... ... ... ... ... ... ..T ... ..C ... ... ... ...

#pipiens1-3              ... ... ... ... ... ... ... ... ... ... ... ... ... ... ... ... ... ... ... ..T ... ..C ... ... ... ...

#pipiens2-1              ... ... ... ... ... ... ... ... ... ... ... ... ... ... ... ... ... ... ... ..T ... ..C ... ... ... ...

#pipiens2-2              ... ... ... ... ... ... ... ... ... ... ... ... ... ... ... ... ... ... ... ..T ... ..C ... ... ... ...

#pipiens2-3              ... ... ... ... ... ... ... ... ... ... ... ... ... ... ... ... ... ... ... ..T ... ..C ... ... ... ...

#pipiens3-1              ... ... ... ... ... ... ... ... ... ... ... ... ... ... ... ... ... ... ... ... ... ..C ... ... ... ...
```

```
#pipiens3-2              ... ... ... ... ... ... ... ... ... ... ... ... ... ... ... ... ... ... ... ... ... ..C ... ... ... ...
#pipiens3-3              ... ... ... ... ... ... ... ... ... ... ... ... ... ... ... ... ... ... ... ... ... ..C ... ... ... ...
#pipiensUSA_KM355980     ... ... ... ... ... ... ... ... ... ... ... ... ... ... ... ... ... ... ... ... ... ..C ... ... ... ...
#quinq_CPIJ007193        ... ... ... ... ... ... ... ... ... ... ... ... ... ... ... ... ... ... ... ... ... ... ... ... ... ...

[                        222 222 222 222 222 222 222 222 222 222 222 222 222 222 222 222 222 222 222 222 222 222 222 222 222 222 ]
[                        111 111 111 111 111 222 222 222 222 222 222 222 222 222 222 222 222 222 222 222 222 222 222 222 222 222 ]
[                        888 889 999 999 999 000 000 000 011 111 111 112 222 222 222 333 333 333 344 444 444 445 555 555 555 666 ]
[                        567 890 123 456 789 012 345 678 901 234 567 890 123 456 789 012 345 678 901 234 567 890 123 456 789 012 ]
#molestus2-1             CAA ACC GCC CAG ACC AGC TCG TCC GCC AGC TTC GTC CCT TCG CAC AGC ATC TTC CCA ACG TTG TAC TAC ATC CCG GCC
#molestus2-2             ... ... ... ... ... ... ... ... ... ... ... ... ... ... ... ... ... ... ... ... ... ... ... ... ... ...
#molestus2-3             ... ... ... ... ... ... ... ... ... ... ... ... ... ... ... ... ... ... ... ... ... ... ... ... ... ...
#molestus1-1             ... ... ... ... ... ... ... ... ... ... ... ... ... ... ... ... ... ... ... ... ... ... ... ... ... ...
#molestus1-2             ... ... ... ... ... ... ... ... ... ... ... ... ... ... ... ... ... ... ... ... ... ... ... ... ... ...
#molestus1-3             ... ... ... ... ... ... ... ... ... ... ... ... ... ... ... ... ... ... ... ... ... ... ... ... ... ...
#molestus3-1             ... ... ... ... ... ... ... ... ... ... ... ... ... ... ... ... ... ... ... ... ... ... ... ... ... ...
#molestus3-2             ... ... ... ... ... ... ... ... ... ... ... ... ... ... ... ... ... ... ... ... ... ... ... ... ... ...
#molestus3-3             ... ... ... ... ... ... ... ... ... ... ... ... ... ... ... ... ... ... ... ... ... ... ... ... ... ...
#pipiens1-1              ... ... ... ... ... ... ... ... ... ... ... ... ... ... ... ... ... ... ... ... ... ... ... ... ... ...
#pipiens1-2              ... ... ... ... ... ... ... ... ... ... ... ... ... ... ... ... ... ... ... ... ... ... ... ... ... ...
#pipiens1-3              ... ... ... ... ... ... ... ... ... ... ... ... ... ... ... ... ... ... ... ... ... ... ... ... ... ...
#pipiens2-1              ... ... ... ... ... ... ... ... ... ... ... ... ... ... ... ... ... ... ... ... ... ... ... ... ... ...
#pipiens2-2              ... ... ... ... ... ... ... ... ... ... ... ... ... ... ... ... ... ... ... ... ... ... ... ... ... ...
#pipiens2-3              ... ... ... ... ... ... ... ... ... ... ... ... ... ... ... ... ... ... ... ... ... ... ... ... ... ...
```

```
#pipiens3-1              ... ... ... ... ... ... ... ... ... ... ... ... ... ... ... ... ... ... ... ... ... ... ... ... ... ...

#pipiens3-2              ... ... ... ... ... ... ... ... ... ... ... ... ... ... ... ... ... ... ... ... ... ... ... ... ... ...

#pipiens3-3              ... ... ... ... ... ... ... ... ... ... ... ... ... ... ... ... ... ... ... ... ... ... ... ... ... ...

#pipiensUSA_KM355980     ... ... ... ... ... ... ... ... ... ... ... ... ... ... ... .A. ... ... ... ... ... ... ... ... ... ...

#quinq_CPIJ007193        ... ... ... ... ... ... ... ... ... ... ... ... ... ... ... ... ... ... ... ... ... ... ... ... ... ---


[                        222 222 222 222 222 222 222 222 222 222 222 222 222 222 22]

[                        222 222 222 222 222 222 222 222 222 222 222 222 233 333 33]

[                        666 666 677 777 777 778 888 888 888 999 999 999 900 000 00]

[                        345 678 901 234 567 890 123 456 789 012 345 678 901 234 56]

#molestus2-1             GCC GCG GCT CAA CCG ACA CCA ACC AAC CCT ACC CTC AAC CCC AT

#molestus2-2             ... ... ... ... ... ... ... ... ... ... ... ... ... ... ..

#molestus2-3             ... ... ... ... ..A ... ... ... ... ... ... ... ... ... ..

#molestus1-1             ... ... ... ... ... ... ... ... ... ... ... ... ... ... ..

#molestus1-2             ... ... ... ... ..A ... ... ... ... ... ... ... ... ... ..

#molestus1-3             ... ... ... ... ..A ... ... ... ... ... ... ... ... ... ..

#molestus3-1             ... ... ... ... ..A ... ... ... ... ... ... ..A ... ... ..

#molestus3-2             ... ... ... ... ... ... ... ... ... ... ... ... ... ... ..

#molestus3-3             ... ... ... ... ..A ... ... ... ... ... ... ..A ... ... ..

#pipiens1-1              ... ..A ... ... ... .T. ... ... ... ..A ... ... ... ... ..

#pipiens1-2              ... ..A ... ... ... .T. ... ... ... ..A ... ... ... ... ..

#pipiens1-3              ... ..A ... ... ... .T. ... ... ... ..A ... ... ... ... ..

#pipiens2-1              ... ..A ... ... ... .T. ... ... ... ..A ... ... ... ... ..

#pipiens2-2              ... ..A ... ... ... .T. ... ... ... ..A ... ... ... ... ..
```

```
#pipiens2-3             ... ..A ... ... ... .T. ... ... ... ..A ... ... ... ... ..

#pipiens3-1             ... ..A ... ... ... .T. ... ... ... ..A ... ... ... ... ..

#pipiens3-2             ... ..A ... ... ... .T. ... ... ... ..A ... ... ... ... ..

#pipiens3-3             ... ..A ... ... ... .T. ... ... ... ..A ... ... ... ... ..

#pipiensUSA_KM355980    ... ..A ... ... ..A .T. ... ... ... ... ... ..A ... ..A ..

#quinq_CPIJ007193       ... ... ... ... ... ... T.. .T. ... ... ... ..A ... ... ..
```

Aligned sequence data. Nucleotide sequences of *per* gene: exon 2 (positions 1-333), exon 3 (positions 337-1074), exon 4 (positions 1078-2306). Positions of the sites in combined sequences shown on the top.
