# Supplementary material for: Comparative analysis of the circadian rhythm genes period and timeless in Culex pipiens Linnaeus, 1758 (Diptera, Culicidae)
Source: Comp Cytogenet. 2016 Oct 10;10(4):483–504. doi: 10.3897/CompCytogen.v10i4.7582 (PMC5240504; doi:10.3897/CompCytogen.v10i4.7582)
Supplement: Supplementary material 2 — Aligned nucleotide sequences of tim gene. [file CompCytogen-010-483-s002.pdf]

Supplemented file 2.

Aligned nucleotide sequences of *tim* gene. DNA sequences of three clones of each individual *C. pipiens* are presented and compared with sequences of *C. quinquefasciatus* (CPIJ007193) and *C. pipiens* from the USA (KM355980).

```
[                                                                          1111111 1111111111 1]
[                                  1112222 2333344444 5555666677 8888899999 9990112223 3344445555 5]
[                               3574460366 7488903366 0114156847 3345800246 6698122391 1423590122 5]
[                               2074940739 4179884717 6893131300 4711416370 2812910830 9976702436 3]
#molestus2-1                    ACTTTCCTGC ATGACACCGC ATCTTAGGTA ACTGGTAAGA GTCCGAGATT CCGAAGCCTA C
#molestus2-2                    .......... .......... .....G.... .......... .......... .......... .
#molestus2-3                    .......... .......... .......... .......... .......... .......... .
#molestus1-1                    .......... .......... .......... .......... .......... .......... .
#molestus1-2                    .......... .......... .......... .......... .......... .......... .
#molestus1-3                    .......... .......... .......... .......... .......... .......... .
#molestus3-1                    .......... .......... .....G.... .......... .......... .......... .
#molestus3-2                    .......... .......... .....G.... .......... .......... .......... .
#molestus3-3                    .......... .......... .....G.... .......... .......... .......... .
#pipiens1-1                     ..ACCTT... .C........ .....G..A. .........T .GTA...GCC .T.G...... .
#pipiens1-2                     ..ACCTT... .C........ .....G..A. .........T .GTA...GCC ..A...T... .
#pipiens1-3                     ..ACCTT... .C........ .....G..A. .........T .GTA...GCC ..A...T... .
#pipiens2-1                     ..ACCTT..G .C......A. .....G..A. C........T AGTA....CC .T.G...... .
#pipiens2-2                     ..ACCTT..G .C......A. .....G..A. C........T AGTA....CC .T.G...... .
```

```
#pipiens2-3                     ..A.C.T..G .C......A. .....G..A. C.......T AGTA....C. .TA...T... .
#pipiens3-1                     ..ACCTT..G .C......A. .....G..A. C.......T AGTA...GCC .T.G...... .
#pipiens3-2                     ..ACCTT..G .C......A. .....G..A. C.......T AGTA...GCC .T.G...... .
#pipiens3-3                     ..AC.TT..G .C......A. .....G..A. C.......T .GTA....C. ..A...T... .
#pipiensUSA_KM355979            CT...TTCC. T.ATTGTT.T GCTCCGTC.T ---ATATGAT .G........ .....T.TCG T
#quinquefasciatus_CPIJ007082    CT...TTC.. T.A.T..T.T GCTCCGTC.T .TCATATGAT .G.ACGAG.C TT..GT.TCG T
```

Aligned sequence data, only variable sites are shown. Nucleotide sequences of *tim* gene: exon 1 (positions 1-1037), exon 5 (positions 1041-1418), exon 6 (positions 1422-1566). Positions of the variable sites in combined sequences shown on the top.

```
#MEGA
!Title ClonsTimeless exon1,5,6_04.10.txt;
!Format
   DataType=Nucleotide CodeTable=Standard
   NSeqs=20 NSites=1566
   Identical=. Missing=? Indel=-;


[                               12]
#molestus2-1                    AT
#molestus2-2                    ..
#molestus2-3                    ..
#molestus1-1                    ..
#molestus1-2                    ..
```

```
#molestus1-3                        ..

#molestus3-1                        ..

#molestus3-2                        ..

#molestus3-3                        ..

#pipiens1-1                         ..

#pipiens1-2                         ..

#pipiens1-3                         ..

#pipiens2-1                         ..

#pipiens2-2                         ..

#pipiens2-3                         ..

#pipiens3-1                         ..

#pipiens3-2                         ..

#pipiens3-3                         ..

#pipiensUSA_KM355979                ..

#quinquefasciatus_CPIJ007082        ..


!Domain=Data property=Coding CodonStart=1;

[                                            11 111 111 112 222 222 222 333 333 333 344 444 444 445 555 555 555 666 666 666 677 777 777 778 ]

[                                   345 678 901 234 567 890 123 456 789 012 345 678 901 234 567 890 123 456 789 012 345 678 901 234 567 890 ]

#molestus2-1                        GAA GTG TAC CTG GTG AGT GAG GAC TGT CTA GTG AAT CTG GAG GAA ATC ATC GGC AAA TTG GCC GTC GAA GAT ACT ACG

#molestus2-2                        ... ... ... ... ... ... ... ... ... ... ... ... ... ... ... ... ... ... ... ... ... ... ... ... ... ...

#molestus2-3                        ... ... ... ... ... ... ... ... ... ... ... ... ... ... ... ... ... ... ... ... ... ... ... ... ... ...

#molestus1-1                        ... ... ... ... ... ... ... ... ... ... ... ... ... ... ... ... ... ... ... ... ... ... ... ... ... ...

#molestus1-2                        ... ... ... ... ... ... ... ... ... ... ... ... ... ... ... ... ... ... ... ... ... ... ... ... ... ...
```

```
#molestus1-3                  ... ... ... ... ... ... ... ... ... ... ... ... ... ... ... ... ... ... ... ... ... ... ... ... ... ...
#molestus3-1                  ... ... ... ... ... ... ... ... ... ... ... ... ... ... ... ... ... ... ... ... ... ... ... ... ... ...
#molestus3-2                  ... ... ... ... ... ... ... ... ... ... ... ... ... ... ... ... ... ... ... ... ... ... ... ... ... ...
#molestus3-3                  ... ... ... ... ... ... ... ... ... ... ... ... ... ... ... ... ... ... ... ... ... ... ... ... ... ...
#pipiens1-1                   ... ... ... ... ... ... ... ... ... ... ... ... ... ... ... ... ... ... ... ... ... ... ... ... ..A ...
#pipiens1-2                   ... ... ... ... ... ... ... ... ... ... ... ... ... ... ... ... ... ... ... ... ... ... ... ... ..A ...
#pipiens1-3                   ... ... ... ... ... ... ... ... ... ... ... ... ... ... ... ... ... ... ... ... ... ... ... ... ..A ...
#pipiens2-1                   ... ... ... ... ... ... ... ... ... ... ... ... ... ... ... ... ... ... ... ... ... ... ... ... ..A ...
#pipiens2-2                   ... ... ... ... ... ... ... ... ... ... ... ... ... ... ... ... ... ... ... ... ... ... ... ... ..A ...
#pipiens2-3                   ... ... ... ... ... ... ... ... ... ... ... ... ... ... ... ... ... ... ... ... ... ... ... ... ..A ...
#pipiens3-1                   ... ... ... ... ... ... ... ... ... ... ... ... ... ... ... ... ... ... ... ... ... ... ... ... ..A ...
#pipiens3-2                   ... ... ... ... ... ... ... ... ... ... ... ... ... ... ... ... ... ... ... ... ... ... ... ... ..A ...
#pipiens3-3                   ... ... ... ... ... ... ... ... ... ... ... ... ... ... ... ... ... ... ... ... ... ... ... ... ..A ...
#pipiensUSA_KM355979          ... ... ... ... ... ... ... ... ... ..C ... ... ... ... ... ..T ... ... ... ... ... ... ... ... ... ...
#quinquefasciatus_CPIJ007082  ... ... ... ... ... ... ... ... ... ..C ... ... ... ... ... ..T ... ... ... ... ... ... ... ... ... ...

[                                                      11 111 111 111 111 111 111 111 111 111 111 111 111 111 111 111 111 111 111 111 ]
[                             888 888 888 999 999 999 900 000 000 001 111 111 111 222 222 222 233 333 333 334 444 444 444 555 555 555 ]
[                             123 456 789 012 345 678 901 234 567 890 123 456 789 012 345 678 901 234 567 890 123 456 789 012 345 678 ]
#molestus2-1                  CTG AGA ACA TTC CGA AGG GCG ATT GGG TTT GGT CAG AAT GTT AAG AAC GAT ATT GTC CCG TTG TTG GTT CAT GCC AAG
#molestus2-2                  ... ... ... ... ... ... ... ... ... ... ... ... ... ... ... ... ... ... ... ... ... ... ... ... ... ...
#molestus2-3                  ... ... ... ... ... ... ... ... ... ... ... ... ... ... ... ... ... ... ... ... ... ... ... ... ... ...
#molestus1-1                  ... ... ... ... ... ... ... ... ... ... ... ... ... ... ... ... ... ... ... ... ... ... ... ... ... ...
#molestus1-2                  ... ... ... ... ... ... ... ... ... ... ... ... ... ... ... ... ... ... ... ... ... ... ... ... ... ...
```

```
#molestus1-3                  ... ... ... ... ... ... ... ... ... ... ... ... ... ... ... ... ... ... ... ... ... ... ... ... ... ...
#molestus3-1                  ... ... ... ... ... ... ... ... ... ... ... ... ... ... ... ... ... ... ... ... ... ... ... ... ... ...
#molestus3-2                  ... ... ... ... ... ... ... ... ... ... ... ... ... ... ... ... ... ... ... ... ... ... ... ... ... ...
#molestus3-3                  ... ... ... ... ... ... ... ... ... ... ... ... ... ... ... ... ... ... ... ... ... ... ... ... ... ...
#pipiens1-1                   ... ... ... ... ... ... ... ... ... ... ... ... ... ... ... ... ... ... ... ... ... C.. ..C ... ... ...
#pipiens1-2                   ... ... ... ... ... ... ... ... ... ... ... ... ... ... ... ... ... ... ... ... ... C.. ..C ... ... ...
#pipiens1-3                   ... ... ... ... ... ... ... ... ... ... ... ... ... ... ... ... ... ... ... ... ... C.. ..C ... ... ...
#pipiens2-1                   ... ... ... ... ... ... ... ... ... ... ... ... ... ... ... ... ... ... ... ... ... C.. ..C ... ... ...
#pipiens2-2                   ... ... ... ... ... ... ... ... ... ... ... ... ... ... ... ... ... ... ... ... ... C.. ..C ... ... ...
#pipiens2-3                   ... ... ... ... ... ... ... ... ... ... ... ... ... ... ... ... ... ... ... ... ... ... ..C ... ... ...
#pipiens3-1                   ... ... ... ... ... ... ... ... ... ... ... ... ... ... ... ... ... ... ... ... ... C.. ..C ... ... ...
#pipiens3-2                   ... ... ... ... ... ... ... ... ... ... ... ... ... ... ... ... ... ... ... ... ... C.. ..C ... ... ...
#pipiens3-3                   ... ... ... ... ... ... ... ... ... ... ... ... ... ... ... ... ... ... ... ... ... C.. ... ... ... ...
#pipiensUSA_KM355979          ... ... ... ... ... ... ... ... ... ... ... ... ... ... ... ... ... ... ... ... ... ... ... ... ... ...
#quinquefasciatus_CPIJ007082  ... ... ... ... ... ... ... ... ... ... ... ... ... ... ... ... ... ... ... ... ... ... ... ... ... ...

[                             111 111 111 111 111 111 111 111 111 111 111 111 111 112 222 222 222 222 222 222 222 222 222 222 222 222 ]
[                             566 666 666 667 777 777 777 888 888 888 899 999 999 990 000 000 000 111 111 111 122 222 222 223 333 333 ]
[                             901 234 567 890 123 456 789 012 345 678 901 234 567 890 123 456 789 012 345 678 901 234 567 890 123 456 ]
#molestus2-1                  GAT TCC AAG ATT CTC GAT CCG ACA ATT CGG TTA CTG GTG AAC CTG ACC GTG CCG GCG GAA TGC TTA CTT CCG GTG GAT
#molestus2-2                  ... ... ... ... ... ... ... ... ... ... ... ... ... ... ... ... ... ... ... ... ... ... ... ... ... ...
#molestus2-3                  ... ... ... ... ... ... ... ... ... ... ... ... ... ... ... ... ... ... ... ... ... ... ... ... ... ...
#molestus1-1                  ... ... ... ... ... ... ... ... ... ... ... ... ... ... ... ... ... ... ... ... ... ... ... ... ... ...
#molestus1-2                  ... ... ... ... ... ... ... ... ... ... ... ... ... ... ... ... ... ... ... ... ... ... ... ... ... ...
```

```
#molestus1-3                  ... ... ... ... ... ... ... ... ... ... ... ... ... ... ... ... ... ... ... ... ... ... ... ... ... ...
#molestus3-1                  ... ... ... ... ... ... ... ... ... ... ... ... ... ... ... ... ... ... ... ... ... ... ... ... ... ...
#molestus3-2                  ... ... ... ... ... ... ... ... ... ... ... ... ... ... ... ... ... ... ... ... ... ... ... ... ... ...
#molestus3-3                  ... ... ... ... ... ... ... ... ... ... ... ... ... ... ... ... ... ... ... ... ... ... ... ... ... ...
#pipiens1-1                   ... ..T ... ... ... ... ... ... ... ... ... ... ... ..T ... ... ... ... ... ... ... ... ... ... ... ...
#pipiens1-2                   ... ..T ... ... ... ... ... ... ... ... ... ... ... ..T ... ... ... ... ... ... ... ... ... ... ... ...
#pipiens1-3                   ... ..T ... ... ... ... ... ... ... ... ... ... ... ..T ... ... ... ... ... ... ... ... ... ... ... ...
#pipiens2-1                   ... ..T ... ... ... ... ... ... ... ... ... ... ... ..T ... ... ... ... ... ... ... ... ... ... ... ...
#pipiens2-2                   ... ..T ... ... ... ... ... ... ... ... ... ... ... ..T ... ... ... ... ... ... ... ... ... ... ... ...
#pipiens2-3                   ... ... ... ... ... ... ... ... ... ... ... ... ... ..T ... ... ... ... ... ... ... ... ... ... ... ...
#pipiens3-1                   ... ..T ... ... ... ... ... ... ... ... ... ... ... ..T ... ... ... ... ... ... ... ... ... ... ... ...
#pipiens3-2                   ... ..T ... ... ... ... ... ... ... ... ... ... ... ..T ... ... ... ... ... ... ... ... ... ... ... ...
#pipiens3-3                   ... ..T ... ... ... ... ... ... ... ... ... ... ... ..T ... ... ... ... ... ... ... ... ... ... ... ...
#pipiensUSA_KM355979          ... ..T ... ... ... ... ... ... ... ... ... ... ... ..T ... ... ... ... ... ... ... ... ... ... ... ...
#quinquefasciatus_CPIJ007082  ... ..T ... ... ... ... ... ... ... ... ... ... ... ..T ... ... ... ... ... ... ... ... ... ... ... ...

[                             222 222 222 222 222 222 222 222 222 222 222 222 222 222 222 222 222 222 222 222 222 333 333 333 333 333 ]
[                             333 444 444 444 455 555 555 556 666 666 666 777 777 777 788 888 888 889 999 999 999 000 000 000 011 111 ]
[                             789 012 345 678 901 234 567 890 123 456 789 012 345 678 901 234 567 890 123 456 789 012 345 678 901 234 ]
#molestus2-1                  TTG GTC TCC AAG TCG GAG ATT GGA CGG CAC ACC ATT TAC GAG TTG AAC AAA CTG CTC ATA ACC AGC AAG GAA GCG TTT
#molestus2-2                  ... ... ... ... ... ... ... ... ... ... ... ... ... ... ... ... ... ... ... ... ... ... ... ... ... ...
#molestus2-3                  ... ... ... ... ... ... ... ... ... ... ... ... ... ... ... ... ... ... ... ... ... ... ... ... ... ...
#molestus1-1                  ... ... ... ... ... ... ... ... ... ... ... ... ... ... ... ... ... ... ... ... ... ... ... ... ... ...
#molestus1-2                  ... ... ... ... ... ... ... ... ... ... ... ... ... ... ... ... ... ... ... ... ... ... ... ... ... ...
```

```
#molestus1-3                  ... ... ... ... ... ... ... ... ... ... ... ... ... ... ... ... ... ... ... ... ... ... ... ... ... ...
#molestus3-1                  ... ... ... ... ... ... ... ... ... ... ... ... ... ... ... ... ... ... ... ... ... ... ... ... ... ...
#molestus3-2                  ... ... ... ... ... ... ... ... ... ... ... ... ... ... ... ... ... ... ... ... ... ... ... ... ... ...
#molestus3-3                  ... ... ... ... ... ... ... ... ... ... ... ... ... ... ... ... ... ... ... ... ... ... ... ... ... ...
#pipiens1-1                   ... ... ... ... ... ... ... ... ... ... ... ... ... ... ... ... ... ... ... ... ... ... ... ... ... ...
#pipiens1-2                   ... ... ... ... ... ... ... ... ... ... ... ... ... ... ... ... ... ... ... ... ... ... ... ... ... ...
#pipiens1-3                   ... ... ... ... ... ... ... ... ... ... ... ... ... ... ... ... ... ... ... ... ... ... ... ... ... ...
#pipiens2-1                   ... ... ... ... ... ... ... ... ... ... ..G ... ... ... ... ... ... ... ... ... ... ... ... ... ... ...
#pipiens2-2                   ... ... ... ... ... ... ... ... ... ... ..G ... ... ... ... ... ... ... ... ... ... ... ... ... ... ...
#pipiens2-3                   ... ... ... ... ... ... ... ... ... ... ..G ... ... ... ... ... ... ... ... ... ... ... ... ... ... ...
#pipiens3-1                   ... ... ... ... ... ... ... ... ... ... ..G ... ... ... ... ... ... ... ... ... ... ... ... ... ... ...
#pipiens3-2                   ... ... ... ... ... ... ... ... ... ... ..G ... ... ... ... ... ... ... ... ... ... ... ... ... ... ...
#pipiens3-3                   ... ... ... ... ... ... ... ... ... ... ..G ... ... ... ... ... ... ... ... ... ... ... ... ... ... ...
#pipiensUSA_KM355979          C.. ... ... ... ... ... ... ... ..C ... ... ... .T. ... ... ... ... ... ... ... ... ... ... ... ... ...
#quinquefasciatus_CPIJ007082  C.. ... ... ... ... ... ... ... ... ... ... ... .T. ... ... ... ... ... ... ... ... ... ... ... ... ...

[                             333 333 333 333 333 333 333 333 333 333 333 333 333 333 333 333 333 333 333 333 333 333 333 333 333 333 ]
[                             111 112 222 222 222 333 333 333 344 444 444 445 555 555 555 666 666 666 677 777 777 778 888 888 888 999 ]
[                             567 890 123 456 789 012 345 678 901 234 567 890 123 456 789 012 345 678 901 234 567 890 123 456 789 012 ]
#molestus2-1                  GTC GAC TGG AAA ACA ACC AAG GCA GTT ATC GAT TAT ATG AAG TCC ATC CTC GAG AAG GAC AGC AAG CTA TCG GTA CAG
#molestus2-2                  ... ... ... ... ... ... ... ... ... ... ... ... ... ... ... ... ... ... ... ... ... ... ... ... ... ...
#molestus2-3                  ... ... ... ... ... ... ... ... ... ... ... ... ... ... ... ... ... ... ... ... ... ... ... ... ... ...
#molestus1-1                  ... ... ... ... ... ... ... ... ... ... ... ... ... ... ... ... ... ... ... ... ... ... ... ... ... ...
#molestus1-2                  ... ... ... ... ... ... ... ... ... ... ... ... ... ... ... ... ... ... ... ... ... ... ... ... ... ...
```

```
#molestus1-3                      ... ... ... ... ... ... ... ... ... ... ... ... ... ... ... ... ... ... ... ... ... ... ... ... ... ...
#molestus3-1                      ... ... ... ... ... ... ... ... ... ... ... ... ... ... ... ... ... ... ... ... ... ... ... ... ... ...
#molestus3-2                      ... ... ... ... ... ... ... ... ... ... ... ... ... ... ... ... ... ... ... ... ... ... ... ... ... ...
#molestus3-3                      ... ... ... ... ... ... ... ... ... ... ... ... ... ... ... ... ... ... ... ... ... ... ... ... ... ...
#pipiens1-1                       ... ... ... ... ... ... ... ... ..C ... ... ... ... ... ... ... ... ... ... ... ... ... ... ... ... ...
#pipiens1-2                       ... ... ... ... ... ... ... ... ..C ... ... ... ... ... ... ... ... ... ... ... ... ... ... ... ... ...
#pipiens1-3                       ... ... ... ... ... ... ... ... ..C ... ... ... ... ... ... ... ... ... ... ... ... ... ... ... ... ...
#pipiens2-1                       ... ... ... ... ... ... ... ... ..C ... ... ... ... ... ... ... ... ... ... ... ... ... ... ... ... ...
#pipiens2-2                       ... ... ... ... ... ... ... ... ..C ... ... ... ... ... ... ... ... ... ... ... ... ... ... ... ... ...
#pipiens2-3                       ... ... ... ... ... ... ... ... ..C ... ... ... ... ... ... ... ... ... ... ... ... ... ... ... ... ...
#pipiens3-1                       ... ... ... ... ... ... ... ... ..C ... ... ... ... ... ... ... ... ... ... ... ... ... ... ... ... ...
#pipiens3-2                       ... ... ... ... ... ... ... ... ..C ... ... ... ... ... ... ... ... ... ... ... ... ... ... ... ... ...
#pipiens3-3                       ... ... ... ... ... ... ... ... ..C ... ... ... ... ... ... ... ... ... ... ... ... ... ... ... ... ...
#pipiensUSA_KM355979              ... ... ... ... ... ... ... ... ... ... ... ... ... ... ... ... ... ... ... ... ... ... ... ... A.T ...
#quinquefasciatus_CPIJ007082      ... ... ... ... ... ... ... ... ... ... ... ... ... ... ... ... ... ... ... ... ... ... ... ... A.. ...

[                                 333 333 344 444 444 444 444 444 444 444 444 444 444 444 444 444 444 444 444 444 444 444 444 444 444 444 ]
[                                 999 999 900 000 000 001 111 111 111 222 222 222 233 333 333 334 444 444 444 555 555 555 566 666 666 667 ]
[                                 345 678 901 234 567 890 123 456 789 012 345 678 901 234 567 890 123 456 789 012 345 678 901 234 567 890 ]
#molestus2-1                      AAT TGC GAC AGT ATT AAC AAT TGT TTG TTG CTA TTA CGG AAC ATC CTG CAT GTT CCT GAG GTG AAC ACG CCT GGC GAA
#molestus2-2                      ... ... ... ... ... ... ... ... ... ... ... ... ... ... ... ... ... ... ... ... ... ... ... ... ... ...
#molestus2-3                      ... ... ... ... ... ... ... ... ... ... ... ... ... ... ... ... ... ... ... ... ... ... ... ... ... ...
#molestus1-1                      ... ... ... ... ... ... ... ... ... ... ... ... ... ... ... ... ... ... ... ... ... ... ... ... ... ...
#molestus1-2                      ... ... ... ... ... ... ... ... ... ... ... ... ... ... ... ... ... ... ... ... ... ... ... ... ... ...
```

```
#molestus1-3                     ... ... ... ... ... ... ... ... ... ... ... ... ... ... ... ... ... ... ... ... ... ... ... ... ... ...
#molestus3-1                     ... ... ... ... ... ... ... ... ... ... ... ... ... ... ... ... ... ... ... ... ... ... ... ... ... ...
#molestus3-2                     ... ... ... ... ... ... ... ... ... ... ... ... ... ... ... ... ... ... ... ... ... ... ... ... ... ...
#molestus3-3                     ... ... ... ... ... ... ... ... ... ... ... ... ... ... ... ... ... ... ... ... ... ... ... ... ... ...
#pipiens1-1                      ... ... ... ... ... ... ... ... ... ... ... ... ... ... ... ... ... ... ... ... ... ... ... ... ... ...
#pipiens1-2                      ... ... ... ... ... ... ... ... ... ... ... ... ... ... ... ... ... ... ... ... ... ... ... ... ... ...
#pipiens1-3                      ... ... ... ... ... ... ... ... ... ... ... ... ... ... ... ... ... ... ... ... ... ... ... ... ... ...
#pipiens2-1                      ... ... ... ... ... ... ... ... ... ... ... ... ... ... ... ... ... ... ... ... ... ... ..A ... ... ...
#pipiens2-2                      ... ... ... ... ... ... ... ... ... ... ... ... ... ... ... ... ... ... ... ... ... ... ..A ... ... ...
#pipiens2-3                      ... ... ... ... ... ... ... ... ... ... ... ... ... ... ... ... ... ... ... ... ... ... ..A ... ... ...
#pipiens3-1                      ... ... ... ... ... ... ... ... ... ... ... ... ... ... ... ... ... ... ... ... ... ... ..A ... ... ...
#pipiens3-2                      ... ... ... ... ... ... ... ... ... ... ... ... ... ... ... ... ... ... ... ... ... ... ..A ... ... ...
#pipiens3-3                      ... ... ... ... ... ... ... ... ... ... ... ... ... ... ... ... ... ... ... ... ... ... ..A ... ... ...
#pipiensUSA_KM355979             ... ..T ... ... ... G.. ... ... ... ... ... ... ... ..T ..T ... ... ... ... ... ... ... ... ... ..T ...
#quinquefasciatus_CPIJ007082     ... ..T ... ... ... ... ... ... ... ... ... ... ... ... ..T ... ... ... ... ... ... ... ... ... ..T ...

[                                444 444 444 444 444 444 444 444 444 445 555 555 555 555 555 555 555 555 555 555 555 555 555 555 555 555 ]
[                                777 777 777 888 888 888 899 999 999 990 000 000 000 111 111 111 122 222 222 223 333 333 333 444 444 444 ]
[                                123 456 789 012 345 678 901 234 567 890 123 456 789 012 345 678 901 234 567 890 123 456 789 012 345 678 ]
#molestus2-1                     ACG AAG CCA GCT CAC AGT ACA TCG TTG CAA AAT CAA ATA CTA TGG AAT CTG TTT ACG CAA AGC GTG GAC AAG TTG TTG
#molestus2-2                     ... ... ... ... ... ... ... ... ... ... ... ... ... ... ... ... ... ... ... ... ... ... ... ... ... ...
#molestus2-3                     ... ... ... ... ... ... ... ... ... ... ... ... ... ... ... ... ... ... ... ... ... ... ... ... ... ...
#molestus1-1                     ... ... ... ... ... ... ... ... ... ... ... ... ... ... ... ... ... ... ... ... ... ... ... ... ... ...
#molestus1-2                     ... ... ... ... ... ... ... ... ... ... ... ... ... ... ... ... ... ... ... ... ... ... ... ... ... ...
```

```
#molestus1-3                    ... ... ... ... ... ... ... ... ... ... ... ... ... ... ... ... ... ... ... ... ... ... ... ... ... ...
#molestus3-1                    ... ... ... ... ... ... ... ... ... ... ... ... ... ... ... ... ... ... ... ... ... ... ... ... ... ...
#molestus3-2                    ... ... ... ... ... ... ... ... ... ... ... ... ... ... ... ... ... ... ... ... ... ... ... ... ... ...
#molestus3-3                    ... ... ... ... ... ... ... ... ... ... ... ... ... ... ... ... ... ... ... ... ... ... ... ... ... ...
#pipiens1-1                     ... ... ... ... ... ... ... ... ... ... ... ... ... ... ... ... ... ... ... ... ... ... ... ... ... ...
#pipiens1-2                     ... ... ... ... ... ... ... ... ... ... ... ... ... ... ... ... ... ... ... ... ... ... ... ... ... ...
#pipiens1-3                     ... ... ... ... ... ... ... ... ... ... ... ... ... ... ... ... ... ... ... ... ... ... ... ... ... ...
#pipiens2-1                     ... ... ... ... ... ... ... ... ... ... ... ... ... ... ... ... ... ... ... ... ... ... ... ... ... ...
#pipiens2-2                     ... ... ... ... ... ... ... ... ... ... ... ... ... ... ... ... ... ... ... ... ... ... ... ... ... ...
#pipiens2-3                     ... ... ... ... ... ... ... ... ... ... ... ... ... ... ... ... ... ... ... ... ... ... ... ... ... ...
#pipiens3-1                     ... ... ... ... ... ... ... ... ... ... ... ... ... ... ... ... ... ... ... ... ... ... ... ... ... ...
#pipiens3-2                     ... ... ... ... ... ... ... ... ... ... ... ... ... ... ... ... ... ... ... ... ... ... ... ... ... ...
#pipiens3-3                     ... ... ... ... ... ... ... ... ... ... ... ... ... ... ... ... ... ... ... ... ... ... ... ... ... ...
#pipiensUSA_KM355979            ... ... ... ... ... ... ... ... ... ... ... ..G ... ... ... ..C T.. ... ... ... ... ... ... ... C.. ...
#quinquefasciatus_CPIJ007082    ... ... ... ... ... ... ... ... ... ... ... ..G ... ... ... ..C T.. ... ... ... ... ... ... ... C.. ...

[                               555 555 555 555 555 555 555 555 555 555 555 555 555 555 555 555 555 666 666 666 666 666 666 666 666 666 ]
[                               455 555 555 556 666 666 666 777 777 777 788 888 888 889 999 999 999 000 000 000 011 111 111 112 222 222 ]
[                               901 234 567 890 123 456 789 012 345 678 901 234 567 890 123 456 789 012 345 678 901 234 567 890 123 456 ]
#molestus2-1                    ATC TAC CTG ATG TCT TGT CCT CAG CGG GCA TTC TGG GCC GTT ACG ATG GCT CAG TTG GTG GCT CTG ATG TAC AAA GAT
#molestus2-2                    ... ... ... ... ... ... ... ... ... ... ... ... ... ... ... ... ... ... ... ... ... ... ... ... ... ...
#molestus2-3                    ... ... ... ... ... ... ... ... ... ... ... ... ... ... ... ... ... ... ... ... ... ... ... ... ... ...
#molestus1-1                    ... ... ... ... ... ... ... ... ... ... ... ... ... ... ... ... ... ... ... ... ... ... ... ... ... ...
#molestus1-2                    ... ... ... ... ... ... ... ... ... ... ... ... ... ... ... ... ... ... ... ... ... ... ... ... ... ...
```

```
#molestus1-3                    ... ... ... ... ... ... ... ... ... ... ... ... ... ... ... ... ... ... ... ... ... ... ... ... ... ...
#molestus3-1                    ... ... ... ... ... ... ... ... ... ... ... ... ... ... ... ... ... ... ... ... ... ... ... ... ... ...
#molestus3-2                    ... ... ... ... ... ... ... ... ... ... ... ... ... ... ... ... ... ... ... ... ... ... ... ... ... ...
#molestus3-3                    ... ... ... ... ... ... ... ... ... ... ... ... ... ... ... ... ... ... ... ... ... ... ... ... ... ...
#pipiens1-1                     ... ... ... ... ... ... ... ... ... ... ... ... ... ... ... ... ... ... ... ... ... ... ... ... ... ...
#pipiens1-2                     ... ... ... ... ... ... ... ... ... ... ... ... ... ... ... ... ... ... ... ... ... ... ... ... ... ...
#pipiens1-3                     ... ... ... ... ... ... ... ... ... ... ... ... ... ... ... ... ... ... ... ... ... ... ... ... ... ...
#pipiens2-1                     ... ... ... ... ... ... ... ... ... ... ... ... ... ... ... ... ... ... ... ... ... ... ... ... ... ...
#pipiens2-2                     ... ... ... ... ... ... ... ... ... ... ... ... ... ... ... ... ... ... ... ... ... ... ... ... ... ...
#pipiens2-3                     ... ... ... ... ... ... ... ... ... ... ... ... ... ... ... ... ... ... ... ... ... ... ... ... ... ...
#pipiens3-1                     ... ... ... ... ... ... ... ... ... ... ... ... ... ... ... ... ... ... ... ... ... ... ... ... ... ...
#pipiens3-2                     ... ... ... ... ... ... ... ... ... ... ... ... ... ... ... ... ... ... ... ... ... ... ... ... ... ...
#pipiens3-3                     ... ... ... ... ... ... ... ... ... ... ... ... ... ... ... ... ... ... ... ... ... ... ... ... ... ...
#pipiensUSA_KM355979            ... ... ... ... ... ... ... ... ... ... ... ... ... ... ... ... ... ... ... ... ..C ... ... ... ... ...
#quinquefasciatus_CPIJ007082    ... ... ... ... ... ... ... ... ... ... ... ... ... ... ... ... ... ... ... ... ..C ... ... ... ... ...

[                               666 666 666 666 666 666 666 666 666 666 666 666 666 666 666 666 666 666 666 666 666 666 666 666 677 777 ]
[                               222 333 333 333 344 444 444 445 555 555 555 666 666 666 677 777 777 778 888 888 888 999 999 999 900 000 ]
[                               789 012 345 678 901 234 567 890 123 456 789 012 345 678 901 234 567 890 123 456 789 012 345 678 901 234 ]
#molestus2-1                    CAG CAC ATC AGC ACC CTC CAA AAG CTA CTC AGC TGT TGG TTC GAA TCT ACC CTG TCG GAA AGC TCC GAA GAC AAC GAG
#molestus2-2                    ... ... ... ... ... ... ... ... ..G ... ... ... ... ... ... ... ... ... ... ... ... ... ... ... ... ...
#molestus2-3                    ... ... ... ... ... ... ... ... ... ... ... ... ... ... ... ... ... ... ... ... ... ... ... ... ... ...
#molestus1-1                    ... ... ... ... ... ... ... ... ... ... ... ... ... ... ... ... ... ... ... ... ... ... ... ... ... ...
#molestus1-2                    ... ... ... ... ... ... ... ... ... ... ... ... ... ... ... ... ... ... ... ... ... ... ... ... ... ...
```

```
#molestus1-3                   ... ... ... ... ... ... ... ... ... ... ... ... ... ... ... ... ... ... ... ... ... ... ... ... ... ...

#molestus3-1                   ... ... ... ... ... ... ... ... ..G ... ... ... ... ... ... ... ... ... ... ... ... ... ... ... ... ...

#molestus3-2                   ... ... ... ... ... ... ... ... ..G ... ... ... ... ... ... ... ... ... ... ... ... ... ... ... ... ...

#molestus3-3                   ... ... ... ... ... ... ... ... ..G ... ... ... ... ... ... ... ... ... ... ... ... ... ... ... ... ...

#pipiens1-1                    ... ... ... ... ... ... ... ... ..G ... ... ... ... ... ... ... ... ... ... ... ... ... ... ... ... ...

#pipiens1-2                    ... ... ... ... ... ... ... ... ..G ... ... ... ... ... ... ... ... ... ... ... ... ... ... ... ... ...

#pipiens1-3                    ... ... ... ... ... ... ... ... ..G ... ... ... ... ... ... ... ... ... ... ... ... ... ... ... ... ...

#pipiens2-1                    ... ... ... ... ... ... ... ... ..G ... ... ... ... ... ... ... ... ... ... ... ... ... ... ... ... ...

#pipiens2-2                    ... ... ... ... ... ... ... ... ..G ... ... ... ... ... ... ... ... ... ... ... ... ... ... ... ... ...

#pipiens2-3                    ... ... ... ... ... ... ... ... ..G ... ... ... ... ... ... ... ... ... ... ... ... ... ... ... ... ...

#pipiens3-1                    ... ... ... ... ... ... ... ... ..G ... ... ... ... ... ... ... ... ... ... ... ... ... ... ... ... ...

#pipiens3-2                    ... ... ... ... ... ... ... ... ..G ... ... ... ... ... ... ... ... ... ... ... ... ... ... ... ... ...

#pipiens3-3                    ... ... ... ... ... ... ... ... ..G ... ... ... ... ... ... ... ... ... ... ... ... ... ... ... ... ...

#pipiensUSA_KM355979           ... ... ... ... ... ... ... ... ..G ... ... .T. ... ... ... ... ... ... ..C ... ... ... ... ... ... ...

#quinquefasciatus_CPIJ007082   ... ... ... ... ... ... ... ... ..G ... ... .T. ... ... ... ... ... ... ..C ... ... ... ... ... ... ...


[                              777 777 777 777 777 777 777 777 777 777 777 777 777 777 777 777 777 777 777 777 777 777 777 777 777 777 ]

[                              000 001 111 111 111 222 222 222 233 333 333 334 444 444 444 555 555 555 566 666 666 667 777 777 777 888 ]

[                              567 890 123 456 789 012 345 678 901 234 567 890 123 456 789 012 345 678 901 234 567 890 123 456 789 012 ]

#molestus2-1                   AGC AAC ACT TCG CCA CCA CAG CAG TGC AGT GGA GAT TCT AGT CCC ATG CTG ACG TCG GAC CCG ACA TCG GAC TCA TCC

#molestus2-2                   ... ... ... ... ... ... ... ... ... ... ... ... ... ... ... ... ... ... ... ... ... ... ... ... ... ...

#molestus2-3                   ... ... ... ... ... ... ... ... ... ... ... ... ... ... ... ... ... ... ... ... ... ... ... ... ... ...

#molestus1-1                   ... ... ... ... ... ... ... ... ... ... ... ... ... ... ... ... ... ... ... ... ... ... ... ... ... ...

#molestus1-2                   ... ... ... ... ... ... ... ... ... ... ... ... ... ... ... ... ... ... ... ... ... ... ... ... ... ...
```

```
#molestus1-3                    ... ... ... ... ... ... ... ... ... ... ... ... ... ... ... ... ... ... ... ... ... ... ... ... ... ...
#molestus3-1                    ... ... ... ... ... ... ... ... ... ... ... ... ... ... ... ... ... ... ... ... ... ... ... ... ... ...
#molestus3-2                    ... ... ... ... ... ... ... ... ... ... ... ... ... ... ... ... ... ... ... ... ... ... ... ... ... ...
#molestus3-3                    ... ... ... ... ... ... ... ... ... ... ... ... ... ... ... ... ... ... ... ... ... ... ... ... ... ...
#pipiens1-1                     ... ... ... ... ... ... ... ... ... ... ... ..A ... ... ... ... ... ... ... ... ... ... ... ... ... ...
#pipiens1-2                     ... ... ... ... ... ... ... ... ... ... ... ..A ... ... ... ... ... ... ... ... ... ... ... ... ... ...
#pipiens1-3                     ... ... ... ... ... ... ... ... ... ... ... ..A ... ... ... ... ... ... ... ... ... ... ... ... ... ...
#pipiens2-1                     ... ... ... ... ... ... ... ... ... ... ... ..A ... ... ... ... ... ... ... ... ... ... ... ... ... ...
#pipiens2-2                     ... ... ... ... ... ... ... ... ... ... ... ..A ... ... ... ... ... ... ... ... ... ... ... ... ... ...
#pipiens2-3                     ... ... ... ... ... ... ... ... ... ... ... ..A ... ... ... ... ... ... ... ... ... ... ... ... ... ...
#pipiens3-1                     ... ... ... ... ... ... ... ... ... ... ... ..A ... ... ... ... ... ... ... ... ... ... ... ... ... ...
#pipiens3-2                     ... ... ... ... ... ... ... ... ... ... ... ..A ... ... ... ... ... ... ... ... ... ... ... ... ... ...
#pipiens3-3                     ... ... ... ... ... ... ... ... ... ... ... ..A ... ... ... ... ... ... ... ... ... ... ... ... ... ...
#pipiensUSA_KM355979            ... ... ... ... ... ... ... ... ... ... ... ... ... ... ... ... ... ... ... ... ... ..T ... ... ... ...
#quinquefasciatus_CPIJ007082    ... ... ... ... ... ... ... ... ... ... ... ... ... ... ... ... ... ... ... ... ... ..T ... ... ... ...

[                               777 777 777 777 777 778 888 888 888 888 888 888 888 888 888 888 888 888 888 888 888 888 888 888 888 888 ]
[                               888 888 899 999 999 990 000 000 000 111 111 111 122 222 222 223 333 333 333 444 444 444 455 555 555 556 ]
[                               345 678 901 234 567 890 123 456 789 012 345 678 901 234 567 890 123 456 789 012 345 678 901 234 567 890 ]
#molestus2-1                    GAT AAT GGT GAT AGC TTG ACA CTG GGA TGG GAT CAT CAG TGT ACT CAA ACT ACT CTA CTA ATT CCA GGG AGT TCC AAA
#molestus2-2                    ... ... ... ... ... ... ... ... ... ... ... ... ... ... ... ... ... ... ... ... ... ... ... ... ... ...
#molestus2-3                    ... ... ... ... ... ... ... ... ... ... ... ... ... ... ... ... ... ... ... ... ... ... ... ... ... ...
#molestus1-1                    ... ... ... ... ... ... ... ... ... ... ... ... ... ... ... ... ... ... ... ... ... ... ... ... ... ...
#molestus1-2                    ... ... ... ... ... ... ... ... ... ... ... ... ... ... ... ... ... ... ... ... ... ... ... ... ... ...
```

```
#molestus1-3                   ... ... ... ... ... ... ... ... ... ... ... ... ... ... ... ... ... ... ... ... ... ... ... ... ... ...
#molestus3-1                   ... ... ... ... ... ... ... ... ... ... ... ... ... ... ... ... ... ... ... ... ... ... ... ... ... ...
#molestus3-2                   ... ... ... ... ... ... ... ... ... ... ... ... ... ... ... ... ... ... ... ... ... ... ... ... ... ...
#molestus3-3                   ... ... ... ... ... ... ... ... ... ... ... ... ... ... ... ... ... ... ... ... ... ... ... ... ... ...
#pipiens1-1                    ... ... ... ... ... ... ... ... ... ... ... ... ... ... ... ... ... ... ... ... ... ... ... ... ... ...
#pipiens1-2                    ... ... ... ... ... ... ... ... ... ... ... ... ... ... ... ... ... ... ... ... ... ... ... ... ... ...
#pipiens1-3                    ... ... ... ... ... ... ... ... ... ... ... ... ... ... ... ... ... ... ... ... ... ... ... ... ... ...
#pipiens2-1                    ... ... ... ... ... ... ... ... ... ... ... ... ... ... ... ... ... C.. ... ... ... ... ... ... ... ...
#pipiens2-2                    ... ... ... ... ... ... ... ... ... ... ... ... ... ... ... ... ... C.. ... ... ... ... ... ... ... ...
#pipiens2-3                    ... ... ... ... ... ... ... ... ... ... ... ... ... ... ... ... ... C.. ... ... ... ... ... ... ... ...
#pipiens3-1                    ... ... ... ... ... ... ... ... ... ... ... ... ... ... ... ... ... C.. ... ... ... ... ... ... ... ...
#pipiens3-2                    ... ... ... ... ... ... ... ... ... ... ... ... ... ... ... ... ... C.. ... ... ... ... ... ... ... ...
#pipiens3-3                    ... ... ... ... ... ... ... ... ... ... ... ... ... ... ... ... ... C.. ... ... ... ... ... ... ... ...
#pipiensUSA_KM355979           ... ... --- --- --- --- --- --- --- --- --- --- --- --- --- --- --- --- --- --- --- --- ..A ... ... ...
#quinquefasciatus_CPIJ007082   ... ... ... ... ... ... ... ... ... ... ... ... ... ... ... ... ... ... T.. .C. ... ... ..A ... ... ...

[                              888 888 888 888 888 888 888 888 888 888 888 888 888 999 999 999 999 999 999 999 999 999 999 999 999 999 ]
[                              666 666 666 777 777 777 788 888 888 889 999 999 999 000 000 000 011 111 111 112 222 222 222 333 333 333 ]
[                              123 456 789 012 345 678 901 234 567 890 123 456 789 012 345 678 901 234 567 890 123 456 789 012 345 678 ]
#molestus2-1                   ATG AAC ACC TCC ATT AGC AAG GAG CCG AGT GAG GCC GAG CTG GTT ACC CGA ATG GGG ACC GAA TTT CCG ACG CAG ATC
#molestus2-2                   ... ... ... ... ... ... ... ... ... ... ... ... ... ... ... ... ... ... ... ... ... ... ... ... ... ...
#molestus2-3                   ... ... ... ... ... ... ... ... ... ... ... ... ... ... ... ... ... ... ... ... ... ... ... ... ... ...
#molestus1-1                   ... ... ... ... ... ... ... ... ... ... ... ... ... ... ... ... ... ... ... ... ... ... ... ... ... ...
#molestus1-2                   ... ... ... ... ... ... ... ... ... ... ... ... ... ... ... ... ... ... ... ... ... ... ... ... ... ...
```

```
#molestus1-3                    ... ... ... ... ... ... ... ... ... ... ... ... ... ... ... ... ... ... ... ... ... ... ... ... ... ...

#molestus3-1                    ... ... ... ... ... ... ... ... ... ... ... ... ... ... ... ... ... ... ... ... ... ... ... ... ... ...

#molestus3-2                    ... ... ... ... ... ... ... ... ... ... ... ... ... ... ... ... ... ... ... ... ... ... ... ... ... ...

#molestus3-3                    ... ... ... ... ... ... ... ... ... ... ... ... ... ... ... ... ... ... ... ... ... ... ... ... ... ...

#pipiens1-1                     ... ... ... ... ... ... ... ... ... ... ... ... ... ... ... ... ... ... ... ... ... ... ... ... ... ...

#pipiens1-2                     ... ... ... ... ... ... ... ... ... ... ... ... ... ... ... ... ... ... ... ... ... ... ... ... ... ...

#pipiens1-3                     ... ... ... ... ... ... ... ... ... ... ... ... ... ... ... ... ... ... ... ... ... ... ... ... ... ...

#pipiens2-1                     ... ... ... ... ... ... ... ... ... ... ... ... ... ... ... ... ... ... ... ... ... ... ... ... ... ...

#pipiens2-2                     ... ... ... ... ... ... ... ... ... ... ... ... ... ... ... ... ... ... ... ... ... ... ... ... ... ...

#pipiens2-3                     ... ... ... ... ... ... ... ... ... ... ... ... ... ... ... ... ... ... ... ... ... ... ... ... ... ...

#pipiens3-1                     ... ... ... ... ... ... ... ... ... ... ... ... ... ... ... ... ... ... ... ... ... ... ... ... ... ...

#pipiens3-2                     ... ... ... ... ... ... ... ... ... ... ... ... ... ... ... ... ... ... ... ... ... ... ... ... ... ...

#pipiens3-3                     ... ... ... ... ... ... ... ... ... ... ... ... ... ... ... ... ... ... ... ... ... ... ... ... ... ...

#pipiensUSA_KM355979            ... ... ... ... ... ... ... ..T ... ... ... ... ... .A. ... T.. ... ... ... ... ..G ... ... ... ... ...

#quinquefasciatus_CPIJ007082    ... ... ... ... ... ... ... ..T ... ... ... ... ... .A. ... T.. ... ... ... ... ..G ... ... ... ... ...


[                                                                                                    11 111 111 111 111 111 ]

[                               999 999 999 999 999 999 999 999 999 999 999 999 999 999 999 999 999 999 999 999 900 000 000 000 000 000 ]

[                               344 444 444 445 555 555 555 666 666 666 677 777 777 778 888 888 888 999 999 999 900 000 000 001 111 111 ]

[                               901 234 567 890 123 456 789 012 345 678 901 234 567 890 123 456 789 012 345 678 901 234 567 890 123 456 ]

#molestus2-1                    ATC GTG GCG CGA GCC ATC AAA ACG CAT CAT ATA TAC CAT CAA ACG ATG GGA ACC ACC TGC GCA GCT AAG CAT CCA ACA

#molestus2-2                    ... ... ... ... ... ... ... ... ... ... ... ... ... ... ... ... ... ... ... ... ... ... ... ... ... ...

#molestus2-3                    ... ... ... ... ... ... ... ... ... ... ... ... ... ... ... ... ... ... ... ... ... ... ... ... ... ...

#molestus1-1                    ... ... ... ... ... ... ... ... ... ... ... ... ... ... ... ... ... ... ... ... ... ... ... ... ... ...
```

```
#molestus1-2                      ... ... ... ... ... ... ... ... ... ... ... ... ... ... ... ... ... ... ... ... ... ... ... ... ... ...

#molestus1-3                      ... ... ... ... ... ... ... ... ... ... ... ... ... ... ... ... ... ... ... ... ... ... ... ... ... ...

#molestus3-1                      ... ... ... ... ... ... ... ... ... ... ... ... ... ... ... ... ... ... ... ... ... ... ... ... ... ...

#molestus3-2                      ... ... ... ... ... ... ... ... ... ... ... ... ... ... ... ... ... ... ... ... ... ... ... ... ... ...

#molestus3-3                      ... ... ... ... ... ... ... ... ... ... ... ... ... ... ... ... ... ... ... ... ... ... ... ... ... ...

#pipiens1-1                       ... ... ... ... ... ... ... T.. ... ..G ... ... ... ... ... ... ... .T. ... ... ... ... ... ... ... ...

#pipiens1-2                       ... ... ... ... ... ... ... T.. ... ..G ... ... ... ... ... ... ... .T. ... ... ... ... ... ... ... ...

#pipiens1-3                       ... ... ... ... ... ... ... T.. ... ..G ... ... ... ... ... ... ... .T. ... ... ... ... ... ... ... ...

#pipiens2-1                       ... ... ... ... ... ... ... T.A ... ..G ... ... ... ... ... ... ... .T. ... ... ... ... ... ... ... ...

#pipiens2-2                       ... ... ... ... ... ... ... T.A ... ..G ... ... ... ... ... ... ... .T. ... ... ... ... ... ... ... ...

#pipiens2-3                       ... ... ... ... ... ... ... T.A ... ..G ... ... ... ... ... ... ... .T. ... ... ... ... ... ... ... ...

#pipiens3-1                       ... ... ... ... ... ... ... T.A ... ..G ... ... ... ... ... ... ... .T. ... ... ... ... ... ... ... ...

#pipiens3-2                       ... ... ... ... ... ... ... T.A ... ..G ... ... ... ... ... ... ... .T. ... ... ... ... ... ... ... ...

#pipiens3-3                       ... ... ... ... ... ... ... T.. ... ..G ... ... ... ... ... ... ... .T. ... ... ... ... ... ... ... ...

#pipiensUSA_KM355979              ... ... ..A ... ... ... ... T.. ... ..G ... ... ... ... ... ... ... ... ... ... ... ... ... ... ... ...

#quinquefasciatus_CPIJ007082      ... ... ..A ... ... ... ... T.. ... ..G ... ... ... ... ... ... ... ... ... ... ... ... ... ... ... ...


[                                 111 111 111 111 111 111 111 111 111 111 111 111 111 111 111 111 111 111 111 111 111 111 111 111 111 111 ]

[                                 000 000 000 000 000 000 000 000 000 000 000 000 000 000 000 000 000 000 000 000 000 000 000 000 000 000 ]

[                                 111 222 222 222 233 333 333 334 444 444 444 555 555 555 566 666 666 667 777 777 777 888 888 888 899 999 ]

[                                 789 012 345 678 901 234 567 890 123 456 789 012 345 678 901 234 567 890 123 456 789 012 345 678 901 234 ]

#molestus2-1                      CAA CCA GCC CAT CCG CAT GAT --- AGC CCG CTA ACG CTG ACG AGC ACG CTG CCA GAC CTG AGC TCC CAA CTG CTG TCG

#molestus2-2                      ... ... ... ... ... ... ... --- ... ... ... ... ... ... ... ... ... ... ... ... ... ... ... ... ... ...

#molestus2-3                      ... ... ... ... ... ... ... --- ... ... ... ... ... ... ... ... ... ... ... ... ... ... ... ... ... ...
```

```
#molestus1-1                   ... ... ... ... ... ... ... --- ... ... ... ... ... ... ... ... ... ... ... ... ... ... ... ... ... ...
#molestus1-2                   ... ... ... ... ... ... ... --- ... ... ... ... ... ... ... ... ... ... ... ... ... ... ... ... ... ...
#molestus1-3                   ... ... ... ... ... ... ... --- ... ... ... ... ... ... ... ... ... ... ... ... ... ... ... ... ... ...
#molestus3-1                   ... ... ... ... ... ... ... --- ... ... ... ... ... ... ... ... ... ... ... ... ... ... ... ... ... ...
#molestus3-2                   ... ... ... ... ... ... ... --- ... ... ... ... ... ... ... ... ... ... ... ... ... ... ... ... ... ...
#molestus3-3                   ... ... ... ... ... ... ... --- ... ... ... ... ... ... ... ... ... ... ... ... ... ... ... ... ... ...
#pipiens1-1                    ... ... ... ... ... ... ... --- ... ... ... ... ... ... ... ... ... ... ... ... ... ..A ... ... ... ...
#pipiens1-2                    ... ... ... ... ... ... ... --- ... ... ... ... ... ... ... ... ... ... ... ... ... ..A ... ... ... ...
#pipiens1-3                    ... ... ... ... ... ... ... --- ... ... ... ... ... ... ... ... ... ... ... ... ... ..A ... ... ... ...
#pipiens2-1                    ... ... ... ... ... ... ... --- ... ... ... ... ... ... ... ... ... ... ... ... ... ..A ... ... ... ...
#pipiens2-2                    ... ... ... ... ... ... ... --- ... ... ... ... ... ... ... ... ... ... ... ... ... ..A ... ... ... ...
#pipiens2-3                    ... ... ... ... ... ... ... --- ... ... ... ... ... ... ... ... ... ... ... ... ... ..A ... ... ... ...
#pipiens3-1                    ... ... ... ... ... ... ... --- ... ... ... ... ... ... ... ... ... ... ... ... ... ..A ... ... ... ...
#pipiens3-2                    ... ... ... ... ... ... ... --- ... ... ... ... ... ... ... ... ... ... ... ... ... ..A ... ... ... ...
#pipiens3-3                    ... ... ... ... ... ... ... --- ... ... ... ... ... ... ... ... ... ... ... ... ... ..A ... ... ... ...
#pipiensUSA_KM355979           ... ... ... ... ... ... ... --- ... ... ... ... ... ... ... ... ... ... ... ... ... ... ... ... ... ...
#quinquefasciatus_CPIJ007082   ... ... ... ... ... ... ... --- ... ... ... ... ... ... ... ... ... ... ... ... ... ..A ... ... ... ...

[                              111 111 111 111 111 111 111 111 111 111 111 111 111 111 111 111 111 111 111 111 111 111 111 111 111 111 ]
[                              000 001 111 111 111 111 111 111 111 111 111 111 111 111 111 111 111 111 111 111 111 111 111 111 111 111 ]
[                              999 990 000 000 000 111 111 111 122 222 222 223 333 333 333 444 444 444 455 555 555 556 666 666 666 777 ]
[                              567 890 123 456 789 012 345 678 901 234 567 890 123 456 789 012 345 678 901 234 567 890 123 456 789 012 ]
#molestus2-1                   AGT AAT CTG ATG AGC ACG TGG ACT GAA GAG GAT AAG GAT TCG CTC CAG TGG TAC TAC GTT CAG TGC AAG CAG AGC AAG
#molestus2-2                   ... ... ... ... ... ... ... ... ... ... ... ... ... ... ... ... ... ... ... ... ... ... ... ... ... ...
```

```
#molestus2-3                 ... ... ... ... ... ... ... ... ... ... ... ... ... ... ... ... ... ... ... ... ... ... ... ... ... ...
#molestus1-1                 ... ... ... ... ... ... ... ... ... ... ... ... ... ... ... ... ... ... ... ... ... ... ... ... ... ...
#molestus1-2                 ... ... ... ... ... ... ... ... ... ... ... ... ... ... ... ... ... ... ... ... ... ... ... ... ... ...
#molestus1-3                 ... ... ... ... ... ... ... ... ... ... ... ... ... ... ... ... ... ... ... ... ... ... ... ... ... ...
#molestus3-1                 ... ... ... ... ... ... ... ... ... ... ... ... ... ... ... ... ... ... ... ... ... ... ... ... ... ...
#molestus3-2                 ... ... ... ... ... ... ... ... ... ... ... ... ... ... ... ... ... ... ... ... ... ... ... ... ... ...
#molestus3-3                 ... ... ... ... ... ... ... ... ... ... ... ... ... ... ... ... ... ... ... ... ... ... ... ... ... ...
#pipiens1-1                  ... ... ... ... ... ... ... ... ... ... ... ... ... ... ... ... ... ... ... ... ... ... ... ... ... ...
#pipiens1-2                  ... ... ... ... ... ... ... ... ... ... ... ... ... ... ... ... ... ... ... ... ... ... ... ... ... ...
#pipiens1-3                  ... ... ... ... ... ... ... ... ... ... ... ... ... ... ... ... ... ... ... ... ... ... ... ... ... ...
#pipiens2-1                  ... ... ... ... ... ... ... ... ... ... ... ... ... ... ... ... ... ... ... ... ... ... ... ... ... ...
#pipiens2-2                  ... ... ... ... ... ... ... ... ... ... ... ... ... ... ... ... ... ... ... ... ... ... ... ... ... ...
#pipiens2-3                  ... ... ... ... ... ... ... ... ... ... ... ... ... ... ... ... ... ... ... ... ... ... ... ... ... ...
#pipiens3-1                  ... ... ... ... ... ... ... ... ... ... ... ... ... ... ... ... ... ... ... ... ... ... ... ... ... ...
#pipiens3-2                  ... ... ... ... ... ... ... ... ... ... ... ... ... ... ... ... ... ... ... ... ... ... ... ... ... ...
#pipiens3-3                  ... ... ... ... ... ... ... ... ... ... ... ... ... ... ... ... ... ... ... ... ... ... ... ... ... ...
#pipiensUSA_KM355979         ... ... ... ... ... ... ... ... ... ... ... ... ... ... ... ... ... ... ... ... ... ... ... ... ... ...
#quinquefasciatus_CPIJ007082 ... ... ... ... ... ... ... ... C.G ... ... ... ... ... ... ... ... ... ... ... ... ... ... ... ... ...

[                            111 111 111 111 111 111 111 111 111 111 111 111 111 111 111 111 111 111 111 111 111 111 111 111 111 111 ]
[                            111 111 111 111 111 111 111 111 111 222 222 222 222 222 222 222 222 222 222 222 222 222 222 222 222 222 ]
[                            777 777 788 888 888 889 999 999 999 000 000 000 011 111 111 112 222 222 222 333 333 333 344 444 444 445 ]
[                            345 678 901 234 567 890 123 456 789 012 345 678 901 234 567 890 123 456 789 012 345 678 901 234 567 890 ]
#molestus2-1                 TGT ATG GTT GCC GAT ATC CTG CGA CTG TTT GAG GAG AAC GGA AAC CAG CAA AAG ACT CGG GTA TCA ATT ATC GAG CAG
```

#molestus2-2 ... ...

#molestus2-3 ... ...

#molestus1-1 ... ...

#molestus1-2 ... ...

#molestus1-3 ... ...

#molestus3-1 ... ...

#molestus3-2 ... ...

#molestus3-3 ... ...

#pipiens1-1 ... ...G ... ...

#pipiens1-2 ... ...G ... ...

#pipiens1-3 ... ...G ... ...

#pipiens2-1 ... ...

#pipiens2-2 ... ...

#pipiens2-3 ... ...

#pipiens3-1 ... ...G ... ...

#pipiens3-2 ... ...G ... ...

#pipiens3-3 ... ...

#pipiensUSA_KM355979 ... ...

#quinquefasciatus_CPIJ007082 ... ...A ... ...G ... ...

[111 111 111 111 111 111 111 111 111 111 111 111 111 111 111 111 111 111 111 111 111 111 111 111 111 111]

[222 222 222 222 222 222 222 222 222 222 222 222 222 222 222 222 233 333 333 333 333 333 333 333 333 333]

[555 555 555 666 666 666 677 777 777 778 888 888 888 999 999 999 900 000 000 001 111 111 111 222 222 222]

[123 456 789 012 345 678 901 234 567 890 123 456 789 012 345 678 901 234 567 890 123 456 789 012 345 678]

```
#molestus2-1                      CTG TGG GAG CAG GAC ATT GTC ACG TTG TCC CAG TAC GAT GAG TTG ATG AAG CTG GAG AAT CCG GAC TAC GAG CGG AAC
#molestus2-2                      ... ... ... ... ... ... ... ... ... ... ... ... ... ... ... ... ... ... ... ... ... ... ... ... ... ...
#molestus2-3                      ... ... ... ... ... ... ... ... ... ... ... ... ... ... ... ... ... ... ... ... ... ... ... ... ... ...
#molestus1-1                      ... ... ... ... ... ... ... ... ... ... ... ... ... ... ... ... ... ... ... ... ... ... ... ... ... ...
#molestus1-2                      ... ... ... ... ... ... ... ... ... ... ... ... ... ... ... ... ... ... ... ... ... ... ... ... ... ...
#molestus1-3                      ... ... ... ... ... ... ... ... ... ... ... ... ... ... ... ... ... ... ... ... ... ... ... ... ... ...
#molestus3-1                      ... ... ... ... ... ... ... ... ... ... ... ... ... ... ... ... ... ... ... ... ... ... ... ... ... ...
#molestus3-2                      ... ... ... ... ... ... ... ... ... ... ... ... ... ... ... ... ... ... ... ... ... ... ... ... ... ...
#molestus3-3                      ... ... ... ... ... ... ... ... ... ... ... ... ... ... ... ... ... ... ... ... ... ... ... ... ... ...
#pipiens1-1                       ... ... ... ... ... ... ... ... ... ... ... ... ... ... C.. ... ... ... ... ..C ... ... ... ... ... ...
#pipiens1-2                       ... ... ... ... ... ... ... ... ... ... ... ... ... ... C.. ... ... ... ... ..C ... ... ... ... ... ...
#pipiens1-3                       ... ... ... ... ... ... ... ... ... ... ... ... ... ... C.. ... ... ... ... ..C ... ... ... ... ... ...
#pipiens2-1                       ... ... ... ... ... ... ... ... ... ... ... ... ... ... C.. ... ... ... ... ..C ... ... ... ... ... ...
#pipiens2-2                       ... ... ... ... ... ... ... ... ... ... ... ... ... ... C.. ... ... ... ... ..C ... ... ... ... ... ...
#pipiens2-3                       ... ... ... ... ... ... ... ... ... ... ... ... ... ... C.. ... ... ... ... ... ... ... ... ... ... ...
#pipiens3-1                       ... ... ... ... ... ... ... ... ... ... ... ... ... ... C.. ... ... ... ... ..C ... ... ... ... ... ...
#pipiens3-2                       ... ... ... ... ... ... ... ... ... ... ... ... ... ... C.. ... ... ... ... ..C ... ... ... ... ... ...
#pipiens3-3                       ... ... ... ... ... ... ... ... ... ... ... ... ... ... C.. ... ... ... ... ... ... ... ... ... ... ...
#pipiensUSA_KM355979              ... ... ... ... ... ... ... ... ... ... ... ... ... ... ... ... ... ... ... ... ... ... ... ... ... ...
#quinquefasciatus_CPIJ007082      ... ... ... ... ... ... ... ... ... ... ... ... ... ... ... ... ... ... ... ..C ... ... ..T ... ... ...

[                                 111 111 111 111 111 111 111 111 111 111 111 111 111 111 111 111 111 111 111 111 111 111 111 111 111 111 ]
[                                 333 333 333 333 333 333 333 333 333 333 333 333 333 333 333 333 333 333 333 333 333 333 333 334 444 444 ]
[                                 233 333 333 334 444 444 444 555 555 555 566 666 666 667 777 777 777 888 888 888 899 999 999 990 000 000 ]
```

```
[                                 901 234 567 890 123 456 789 012 345 678 901 234 567 890 123 456 789 012 345 678 901 234 567 890 123 456 ]
#molestus2-1                      GTG CAA ACG CCG GGC TTT TCC GTG GCC TCG GCC AAG TCG GAA AAA ATG GAC GAC GAT AAC CGA CCG TCA TCC AAG GCG
#molestus2-2                      ... ... ... ... ... ... ... ... ... ... ... ... ... ... ... ... ... ... ... ... ... ... ... ... ... ...
#molestus2-3                      ... ... ... ... ... ... ... ... ... ... ... ... ... ... ... ... ... ... ... ... ... ... ... ... ... ...
#molestus1-1                      ... ... ... ... ... ... ... ... ... ... ... ... ... ... ... ... ... ... ... ... ... ... ... ... ... ...
#molestus1-2                      ... ... ... ... ... ... ... ... ... ... ... ... ... ... ... ... ... ... ... ... ... ... ... ... ... ...
#molestus1-3                      ... ... ... ... ... ... ... ... ... ... ... ... ... ... ... ... ... ... ... ... ... ... ... ... ... ...
#molestus3-1                      ... ... ... ... ... ... ... ... ... ... ... ... ... ... ... ... ... ... ... ... ... ... ... ... ... ...
#molestus3-2                      ... ... ... ... ... ... ... ... ... ... ... ... ... ... ... ... ... ... ... ... ... ... ... ... ... ...
#molestus3-3                      ... ... ... ... ... ... ... ... ... ... ... ... ... ... ... ... ... ... ... ... ... ... ... ... ... ...
#pipiens1-1                       ... ... ... ... ... ... ..T ... ... ... ... ... ... ... ... ... ... ... ... ... ... ... ... ... ... ...
#pipiens1-2                       ... ... ... ... ... ... ... ... ... ... ... ... ... ... ... ... ... ... ... ... ... ... ... ... ... ...
#pipiens1-3                       ... ... ... ... ... ... ... ... ... ... ... ... ... ... ... ... ... ... ... ... ... ... ... ... ... ...
#pipiens2-1                       ... ... ... ... ... ... ..T ... ... ... ... ... ... ... ... ... ... ... ... ... ... ... ... ... ... ...
#pipiens2-2                       ... ... ... ... ... ... ..T ... ... ... ... ... ... ... ... ... ... ... ... ... ... ... ... ... ... ...
#pipiens2-3                       ... ... ... ... ... ... ..T ... ... ... ... ... ... ... ... ... ... ... ... ... ... ... ... ... ... ...
#pipiens3-1                       ... ... ... ... ... ... ..T ... ... ... ... ... ... ... ... ... ... ... ... ... ... ... ... ... ... ...
#pipiens3-2                       ... ... ... ... ... ... ..T ... ... ... ... ... ... ... ... ... ... ... ... ... ... ... ... ... ... ...
#pipiens3-3                       ... ... ... ... ... ... ... ... ... ... ... ... ... ... ... ... ... ... ... ... ... ... ... ... ... ...
#pipiensUSA_KM355979              ... ... ... ... ... ... ... ... ... ... ... ... ... ... ... ... ... ... ... ... ... ... ... ... ... ...
#quinquefasciatus_CPIJ007082      ... ... ... ... ... ... ..T ... ... ... ... ... ... ... ... ... ... ... ... ... ... ... ... ... ... ...

[                                 111 111 111 111 111 111 111 111 111 111 111 111 111 111 111 111 111 111 111 111 111 111 111 111 111 111 ]
[                                 444 444 444 444 444 444 444 444 444 444 444 444 444 444 444 444 444 444 444 444 444 444 444 444 444 444 ]
```

```
[                                000 111 111 111 122 222 222 223 333 333 333 444 444 444 455 555 555 556 666 666 666 777 777 777 788 888 ]
[                                789 012 345 678 901 234 567 890 123 456 789 012 345 678 901 234 567 890 123 456 789 012 345 678 901 234 ]
#molestus2-1                     GTT GAT GAC ATA --- GTA CTG CGC GAT CGA TTG TTT AAG GAG AAC AAA GGA AAG CTG GTT GTT TGG TTG CAA AAA AGT
#molestus2-2                     ... ... ... ... --- ... ... ... ... ... ... ... ... ... ... ... ... ... ... ... ... ... ... ... ... ...
#molestus2-3                     ... ... ... ... --- ... ... ... ... ... ... ... ... ... ... ... ... ... ... ... ... ... ... ... ... ...
#molestus1-1                     ... ... ... ... --- ... ... ... ... ... ... ... ... ... ... ... ... ... ... ... ... ... ... ... ... ...
#molestus1-2                     ... ... ... ... --- ... ... ... ... ... ... ... ... ... ... ... ... ... ... ... ... ... ... ... ... ...
#molestus1-3                     ... ... ... ... --- ... ... ... ... ... ... ... ... ... ... ... ... ... ... ... ... ... ... ... ... ...
#molestus3-1                     ... ... ... ... --- ... ... ... ... ... ... ... ... ... ... ... ... ... ... ... ... ... ... ... ... ...
#molestus3-2                     ... ... ... ... --- ... ... ... ... ... ... ... ... ... ... ... ... ... ... ... ... ... ... ... ... ...
#molestus3-3                     ... ... ... ... --- ... ... ... ... ... ... ... ... ... ... ... ... ... ... ... ... ... ... ... ... ...
#pipiens1-1                      ... ... ... ... --- ... ... ... ... ..G ... ... ... ... ... ... ... ... ... ... ... ... ... ... ... ...
#pipiens1-2                      ... ... ... ... --- ... ..A ... ... ... ... ... ... ... ... ... ... ... ... ... ... ... ... ... ... ...
#pipiens1-3                      ... ... ... ... --- ... ..A ... ... ... ... ... ... ... ... ... ... ... ... ... ... ... ... ... ... ...
#pipiens2-1                      ... ... ... ... --- ... ... ... ... ..G ... ... ... ... ... ... ... ... ... ... ... ... ... ... ... ...
#pipiens2-2                      ... ... ... ... --- ... ... ... ... ..G ... ... ... ... ... ... ... ... ... ... ... ... ... ... ... ...
#pipiens2-3                      ... ... ... ... --- ... ..A ... ... ... ... ... ... ... ... ... ... ... ... ... ... ... ... ... ... ...
#pipiens3-1                      ... ... ... ... --- ... ... ... ... ..G ... ... ... ... ... ... ... ... ... ... ... ... ... ... ... ...
#pipiens3-2                      ... ... ... ... --- ... ... ... ... ..G ... ... ... ... ... ... ... ... ... ... ... ... ... ... ... ...
#pipiens3-3                      ... ... ... ... --- ... ..A ... ... ... ... ... ... ... ... ... ... ... ... ... ... ... ... ... ... ...
#pipiensUSA_KM355979             ... ... ... ... --- ... ... ... ... ... ... ... ... ... ... ... ... ... ... .N. ... ... ... ... ... ...
#quinquefasciatus_CPIJ007082     ... ... ... ... --- ... ... ... ... ... ... ... ... ... ... ... ..G ... ... ... ... ... ... ... ... ...

[                                111 111 111 111 111 111 111 111 111 111 111 111 111 111 111 111 111 111 111 111 111 111 111 111 111 111 ]
```

```
[                                   444 444 444 444 444 555 555 555 555 555 555 555 555 555 555 555 555 555 555 555 555 555 555 555 555 555 ]
[                                   888 889 999 999 999 000 000 000 011 111 111 112 222 222 222 333 333 333 344 444 444 445 555 555 555 666 ]
[                                   567 890 123 456 789 012 345 678 901 234 567 890 123 456 789 012 345 678 901 234 567 890 123 456 789 012 ]
#molestus2-1                        TTG CTG GAC TGC TGT TTC GTG AAG CTC AAC CTG CTC AGT GGA ACC GTC GTG GTC ATG GAA CCT GTC CCC TAT CAT TGC
#molestus2-2                        ... ... ... ... ... ... ... ... ... ... ... ... ... ... ... ... ... ... ... ... ... ... ... ... ... ...
#molestus2-3                        ... ... ... ... ... ... ... ... ... ... ... ... ... ... ... ... ... ... ... ... ... ... ... ... ... ...
#molestus1-1                        ... ... ... ... ... ... ... ... ... ... ... ... ... ... ... ... ... ... ... ... ... ... ... ... ... ...
#molestus1-2                        ... ... ... ... ... ... ... ... ... ... ... ... ... ... ... ... ... ... ... ... ... ... ... ... ... ...
#molestus1-3                        ... ... ... ... ... ... ... ... ... ... ... ... ... ... ... ... ... ... ... ... ... ... ... ... ... ...
#molestus3-1                        ... ... ... ... ... ... ... ... ... ... ... ... ... ... ... ... ... ... ... ... ... ... ... ... ... ...
#molestus3-2                        ... ... ... ... ... ... ... ... ... ... ... ... ... ... ... ... ... ... ... ... ... ... ... ... ... ...
#molestus3-3                        ... ... ... ... ... ... ... ... ... ... ... ... ... ... ... ... ... ... ... ... ... ... ... ... ... ...
#pipiens1-1                         ... ... ... ... ... ... ... ... ... ... ... ... ... ... ... ... ... ... ... ... ... ... ... ... ... ...
#pipiens1-2                         ... ... ... ... ... ..T ... ... ... ... ... ... ... ... ... ... ... ... ... ... ... ... ... ... ... ...
#pipiens1-3                         ... ... ... ... ... ..T ... ... ... ... ... ... ... ... ... ... ... ... ... ... ... ... ... ... ... ...
#pipiens2-1                         ... ... ... ... ... ... ... ... ... ... ... ... ... ... ... ... ... ... ... ... ... ... ... ... ... ...
#pipiens2-2                         ... ... ... ... ... ... ... ... ... ... ... ... ... ... ... ... ... ... ... ... ... ... ... ... ... ...
#pipiens2-3                         ... ... ... ... ... ..T ... ... ... ... ... ... ... ... ... ... ... ... ... ... ... ... ... ... ... ...
#pipiens3-1                         ... ... ... ... ... ... ... ... ... ... ... ... ... ... ... ... ... ... ... ... ... ... ... ... ... ...
#pipiens3-2                         ... ... ... ... ... ... ... ... ... ... ... ... ... ... ... ... ... ... ... ... ... ... ... ... ... ...
#pipiens3-3                         ... ... ... ... ... ..T ... ... ... ... ... ... ... ... ... ... ... ... ... ... ... ... ... ... ... ...
#pipiensUSA_KM355979                ... ..T ... ... ... ... ... ... ... ..T ... ... ..C ..G ... ... ... ... ... ... ... ... ..T ... ... ...
#quinquefasciatus_CPIJ007082        ... ..T ... ... ... ... ... ... ... ..T ... ... ..C ..G ... ... ... ... ... ... ... ... ..T ... ... ...
```

[	111 1]
[	555 5]
[	666 6]
[	345 6]
#molestus2-1	ATT C
#molestus2-2	... .
#molestus2-3	... .
#molestus1-1	... .
#molestus1-2	... .
#molestus1-3	... .
#molestus3-1	... .
#molestus3-2	... .
#molestus3-3	... .
#pipiens1-1	... .
#pipiens1-2	... .
#pipiens1-3	... .
#pipiens2-1	... .
#pipiens2-2	... .
#pipiens2-3	... .
#pipiens3-1	... .
#pipiens3-2	... .
#pipiens3-3	... .
#pipiensUSA_KM355979	... .
#quinquefasciatus_CPIJ007082	... .

Aligned sequence data. Nucleotide sequences of *tim* gene: exon 1 (positions 1-1037), exon 5 (positions 1041-1418), exon 6 (positions 1422-1566). Exons 1, 5 and 6 are separated by three dashes (---). Positions of the sites in combined sequences shown on the top.
