# Supplementary material for: Comparative analysis of the circadian rhythm genes period and timeless in Culex pipiens Linnaeus, 1758 (Diptera, Culicidae)
Source: Comp Cytogenet. 2016 Oct 10;10(4):483–504. doi: 10.3897/CompCytogen.v10i4.7582 (PMC5240504; doi:10.3897/CompCytogen.v10i4.7582)
Supplement: Supplementary material 3 — Analysis of the divergent between two forms of Culex pipiens based on comparison of the exon 1 of the gene tim sequences. [file CompCytogen-010-483-s003.pdf]

Supplemented file 3.

Analysis of the divergent between two forms of *C. pipiens* based on comparison of the exon 1 of the gene *tim* sequences.

Aligned nucleotide sequences of the *tim* gene exon1 in compare with sequence of *C. quinquefasciatus* (CPIJ007193).

```
[                                                                       1]
[                        11122 2223333444 4555555666 6677788888 999999990]
[                   3557944603 6774889366 7011245156 7824733458 002466690]
[                   2037649407 9491178717 7689434131 1350047114 163702814]
#molestus1-1        ACCTATTCCT CATTCGCCGC CATCTTCTAG AGGTAACTGG TAAGAGTCT   H1(KU997646)
#molestus1-2        .......... .......... .......... .......... .........
#molestus1-3        .......... .......... .......... .......... .........
#molestus2-1        .......... .......... .......... .......... .........
#molestus2-2        .......... .......... ........G. .......... .........   H2(KU997647)
#molestus2-3        .......... .......... .......... .......... .........
#molestus3-1        .......... .......... ........G. .......... .........
#molestus3-2        .......... .......... ........G. .......... .........
#molestus3-3        .......... .......... ........G. .......... .........
#mV16               .......... .......... .......... .......... .........
#mV17               .......... .......... .......... .......... .........
#mV18               .......... .......... ........G. .......... .........
#mV279              .......... .......... .......... .......... .........
#mV168              .......... .......... .......... .......... .........
#mV48               .......... .......... ........G. .......... .........
#mV49               .......... .......... .......... .......... .........
#mNev5              .......... .......... .......... .......... .........
#mM20               .......... .......... .......... .......... .........
#mNN22              .......... .......... .......... .......... .........
#mNN19              .......... .......... .......... .......... .........
#mNN27              .......... .......... .......... .......... .........
#mV42               .......... .......... .......... .......... .........
#mV51               .......... .......... .......... .......... .........
```

#mV52	....	....	....	....	....	
#mV65	....	....	....	....	....	
#mV70	....	....	........G.	....	....	
#mV80	....	....	....	....	....	
#mV81	....	....	....	....	....	
#cmNN23-1	....	....	....	....	....	
#cmNN23-2	....	....	....	....	....	
#cmNN23-3	....	....	....	....	....	
#cmNN23-5	....	....	....	....	......A.A	H3(KU997649)
#cmNN23-6	....	....	....	....	......A.A	
#cp219-2	....C...T.	....T.....	..C..C..G.	....	......G..	H4(KU997648)
#cp219-4	....C...T.	....T.....	..C..C..G.	....	......G..	
#cp219-10	....C...T.	....T.....	..C..C..G.	....	......G..	
#cp219-16	....C...T.	....T.....	..C..C..G.	....	......G..	
#cm46-1	....	....	........G.	....	....	
#cm46-4	....	....	........G.	....	....	
#cm46-6	....	....	........G.	....	....	
#cm109-1	....	....	....	....	....	
#cm109-5	....	....	....	....	....	
#cm109-6	....	....	....	....	....	
#cp247-3	....	....	....	....	....	
#cp247-5	....	....	....	....	....	
#cm283-1	....	....	........G.	....	....	
#cm283-3	....	....	........G.	....	....	
#cm283-4	....	....	........G.	....	....	
#pipiens1-1	...A.CCTT.	...C......	........G.	...A......	....T.GT.	H5(KU133720)
#pipiens1-2	...A.CCTT.	...C......	........G.	...A......	....T.GT.	
#pipiens1-3	...A.CCTT.	...C......	........G.	...A......	....T.GT.	
#pipiens2-1	...A.CCTT.	G..C....A.	........G.	...A.C....	....TAGT.	H6(KU133722)
#pipiens2-2	...A.CCTT.	G..C....A.	........G.	...A.C....	....TAGT.	
#pipiens2-3	...A..C.T.	G..C....A.	........G.	...A.C....	....TAGT.	H7(KU997650)
#pipiens3-1	...A.CCTT.	G..C....A.	........G.	...A.C....	....TAGT.	
#pipiens3-2	...A.CCTT.	G..C....A.	........G.	...A.C....	....T.GT.	

```
#pV189              ...A.CCTT. G..C....A. ........G. ...A.C.... ....TAGT.

#pipiens3-3         ...A.C.TT. G..C....A. ........G. ...A.C.... ....TAGT.   H8(KU997651)

#pV20               ...A.CCTT. G..C....A. ........G. ...A.C.... ....TTGT.   H9(KU997652)

#cp247-1            ...A...TT. .......... T...C...G. ..A.TC.... ....T.G..  H10(KU997653)

#cp247-2            ...A...TT. .......... T...C...G. ..A.TC.... ....T.G..

#cm46-2             ..T..CCT.. ...C...... ........G. ..A.T..... ....TAG..

#cp219-1            ...A.CCTT. ...C...... T.....T.G. ..A....... ....T.G..  H11(KU997654)

#cp219-3            ...A.CCTT. ...C...... T.....T.G. ..A....... ....T.G..

#cp219-7            ...A.CCTT. ...C...... T.....T.G. ..A....... ....T.G..

#pV13               ...A.CCTT. .......... T...C...G. ..A.T..... ....T.G..  H12(KU997655)

#pV14               ...A...TT. ..C....... T...C...G. ..A.T..... ....T.G..  H13(KU997656)

#pV196              ...A...TT. ...C...... T...C...G. ..A.T..... ....TAGT.  H14(KU997657)

#pIk3.4             .....CCTT. ...C....A. ........G. ...A.---.. ....T.GT.  H15(KU997658)

#cm109-2            .......TT. .......... ....C...G. .......... ....TTG..  H16(KU997659)

#cm109-3            .......TT. .......... ....C...G. .......... ....TTG..

#cm283-2            ...A...TT. ..CC...... ........G. ..A....... ....T.G..  H17(KU997660)

#cm283-5            ...A...TT. ..CC...... ........G. ..A....... ....T.G..

#pV202              ...A...TT. .......... ....C...G. G.A....... ....T.G..  H18(KU997661)

#pV16               ..T..CCT.. ...C...... ........G. ..A.T..... ....TAG..  H19(KU997662)

#pV12               ...A...TT. .......... ........G. ....T..... ....T.G..  H20(KU997663)

#pV15               ...A.CCTT. ...C...... ........G. .......... ....T.GT.  H21(KU997664)

#pIk3.17            ...A.CCTT. ...C....A. ....C...G. .....C.... ....T.G..  H12(KU997665)

#pV180              .......TT. .......... ........G. G.A..C.... ....T.G..  H23(KU997666)

#quinqCPIJ007193    CT.....TTC .T...ATT.T .GCT.C.CGT .C..T.TCAT ATGAT.G..
```

Aligned nucleotide sequences of *tim* gene exon 1 (positions 1-1037) and their GenBank accession numbers, only variable sites are shown. Positions of the variable sites are shown on the top. Haplotypes numbers are shown on the right. "molestus1-1" and "pipiens 2-1" - clons of *C. pipiens* f. *molestus* and f. *pipiens* from Volgograd, respectively, for which long *tim* sequences have been studied; pV - *pipiens*, Volgograd; mV - *molestus*, Volgograd; cm - clons of hybrid between *pipiens* and *molestus* from autogenous population; cp - clons of hybrid between *pipiens* and *molestus* from unautogenous population; mNN - *molestus*, Nizhniy Novgorod, mM - *molestus*, Moscow, mNev - *molestus*, SPeterburg, pIk - *pipiens*, Moscow region, Iksha. Dashes show deletion of 12 nucleotides (sites 831-842) in exon 1 in *C.pipiens* f. *pipiens* from Moscow region.

Aligned amino acid sequences of the *tim* gene exon1 in compare with sequence of *C. quinquefasciatus* (CPIJ007193).

```
[                     112222233 333]
[                    9252478900 223]
[                    1990680402 020]
#molestus1-1         YVPCDTLELT THT
#molestus1-2         .......... ...
#molestus1-3         .......... ...
#molestus2-1         .......... ...
#molestus2-2         .......... ...
#molestus2-3         .......... ...
#molestus3-1         .......... ...
#molestus3-2         .......... ...
#molestus3-3         .......... ...
#mV16                .......... ...
#mV17                .......... ...
#mV18                .......... ...
#mV279               .......... ...
#mV168               .......... ...
#mV48                .......... ...
#mV49                .......... ...
#mNev5               .......... ...
#mM20                .......... ...
#mNN22               .......... ...
#mNN19               .......... ...
#mNN27               .......... ...
#mV42                .......... ...
#mV51                .......... ...
#mV52                .......... ...
#mV65                .......... ...
#mV70                .......... ...
#mV80                .......... ...
#mV81                .......... ...
```

#cmNN23-1	....	...
#cmNN23-2	....	...
#cmNN23-3	....	...
#cmNN23-5	....	.Q.
#cmNN23-6	....	.Q.
#cp219-2	....	.Q.
#cp219-4	....	.Q.
#cp219-10	....	.Q.
#cp219-16	....	.Q.
#cm46-1	....	...
#cm46-4	....	...
#cm46-6	....	...
#cm109-1	....	...
#cm109-5	....	...
#cm109-6	....	...
#cp247-3	....	...
#cp247-5	....	...
#cm283-1	....	...
#cm283-3	....	...
#cm283-4	....	...
#pipiens1-1	....E.....	SQI
#pipiens1-2	....E.....	SQI
#pipiens1-3	....E.....	SQI
#pipiens2-1	....EP....	SQI
#pipiens2-2	....EP....	SQI
#pipiens2-3	....EP....	SQI
#pipiens3-1	....EP....	SQI
#pipiens3-2	....EP....	SQI
#pV189	....EP....	SQI
#pipiens3-3	....EP....	SQI
#pV20	....EP....	SQI

```
#cp247-1            ..S..P.... SQ.
#cp247-2            ..S..P.... SQ.
#cm46-2             .......... SQ.
#cp219-1            ..S....... SQ.
#cp219-3            ..S....... SQ.
#cp219-7            ..S....... SQ.
#pV13               ..S....... SQ.
#pV14               ..S....... SQ.
#pV196              ..S....... SQI
#pIk3.4             ....E--... SQI
#cm109-2            .......... SQ.
#cm109-3            .......... SQ.
#cm283-2            .......... SQ.
#cm283-5            .......... SQ.
#pV202              .......... SQ.
#pV16               .......... SQ.
#pV12               .......... SQ.
#pV15               .......... SQI
#pIk3.17            .....P.... SQ.
#pV180              .....P.... SQ.
#quinqCPIJ007193    FI.F..PDQS SQ.
```

Aligned amino acid sequences of *tim* gene exon 1 (positions 1-1037), only variable sites are shown. Positions of the variable sites are shown on the top. "molestus1-1" and "pipiens 2-1" - clons of *C. pipiens* f. *molestus* and f. *pipiens* from Volgograd, respectively, for which long *tim* sequences have been studied; pV - *pipiens*, Volgograd; mV - *molestus*, Volgograd; cm - clons of hybrid between *pipiens* and *molestus* from autogenous population; cp - clons of hybrid between *pipiens* and *molestus* from unautogenous population; mNN - *molestus*, Nizhniy Novgorod, mM - *molestus*, Moscow, mNev - *molestus*, SPeterburg, pIk - *pipiens*, Moscow region, Iksha. Dashes show deletion of 12 nucleotides (sites 831-842) in exon 1 in *C.pipiens* f. *pipiens* from Moscow region.

Estimates of Evolutionary Divergence over sequence pairs between *Culex pipiens* group members based on comparison of of gene *tim* exon 1**.**

AA\NA		*gene timeless 1exon*		
		1	2	3
1	molestus		**0.011**	**0.028**

2	pipiens	0,009		**0.030**
3	quin	0.025	0.023	

In upper right section in bold: the number of nucleotide base substitutions (NA) per site from averaging over all sequence pairs between groups is shown. All results are based on the pairwise analysis of 79 sequences. There were a total of 1037 positions of *tim* gene in the final dataset. In lower left section: the number of amino acid substitutions (AA) per site from averaging over all sequence pairs between groups are shown. A total of 345 positions of the gene *tim* were analysed as the final dataset.

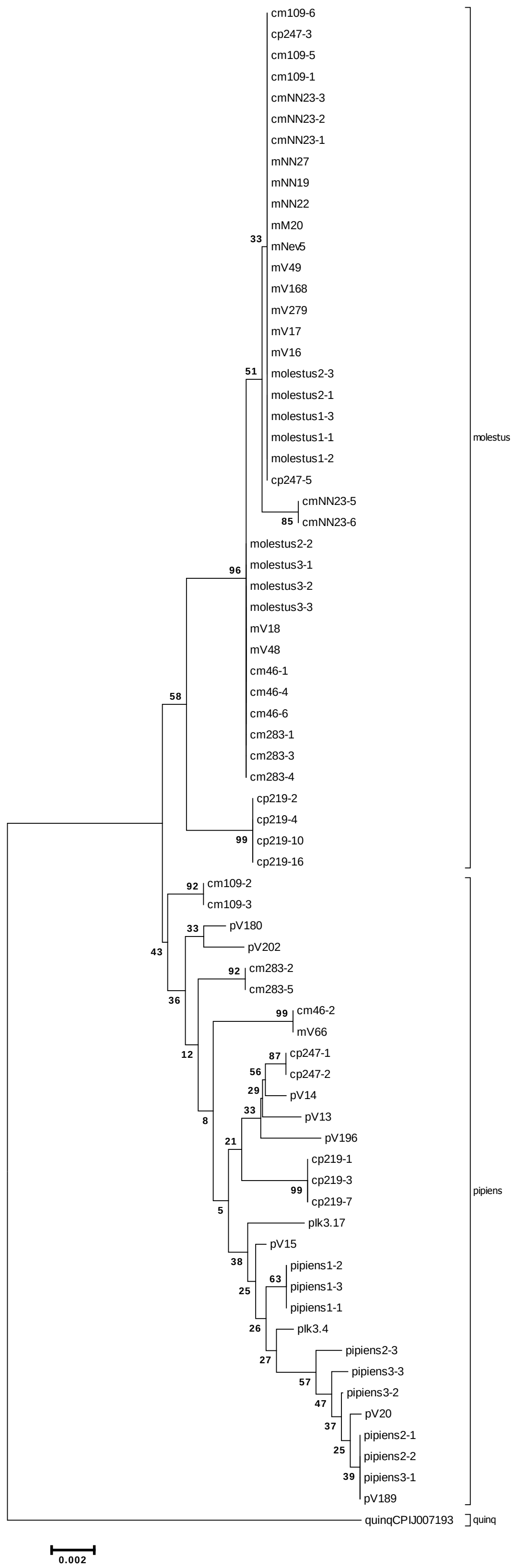

Neighbor-joining tree of *C. pipiens* f. *pipiens, C. pipiens* f. *molestus* based on nucleotide sequences of gene *tim* exon 1with the *C. quinquefasciatus* (CPIJ007193) as the outgroup. Percent bootstrap support based on 1000 replicates. Codon positions included were 1st+2nd+3rd+Noncoding. There were a total of 1037 positions in the final dataset.
