# Supplementary material for: Comparative analysis of the circadian rhythm genes period and timeless in Culex pipiens Linnaeus, 1758 (Diptera, Culicidae)
Source: Comp Cytogenet. 2016 Oct 10;10(4):483–504. doi: 10.3897/CompCytogen.v10i4.7582 (PMC5240504; doi:10.3897/CompCytogen.v10i4.7582)
Supplement: Supplementary material 4 — Aligned tim nucleotide non-coding sequences. [file CompCytogen-010-483-s004.pdf]

Supplemented file 4.

Aligned *tim* nucleotide non-coding sequences. Intron's DNA sequences of individual *C. pipiens* f. *pipiens* and f. *molestus* are presented.

Tim gene, intron 5-6

```
[                      1 1111111112 2222222223 3333333334 4444444445 555555555]
[             1234567890 1234567890 1234567890 1234567890 1234567890 123456789]
#molestus2    GTGCTTTTTG TGTAGGTGTT TTGTTGCAAT ATGGAGATGG TTACCGTTTT GTTCTTCAG
#molestus1    .......... .......... .......... .......... .......... .........
#molestus3    .......... .......... .......... .......... .......... .........
#pipiens1     .......... .......... .......... .......... .......... .........
#pipiens2     .......... .......... .......... .......... .......... .........
#pipiens3     .......... .......... .......... .......... .......... .........
```

Tim gene, intron 6-7

```
[                      1 1111111112 2222222223 3333333334 4444444445 5555555556 6]
[             1234567890 1234567890 1234567890 1234567890 1234567890 1234567890 1]
#molestus2    GTGAGTACTT CTCGGAATTC CCGAACAATT TCGGAGCCCC TTCAACGTTC TTCCCTTTCA G
#molestus1    .......... .......... ......w... .......... .......... .......... .
#molestus3    .......... .......... .......... .......... .......... .......... .
#pipiens1     .......... .......... ......T... .Y........ .......w.. .......... .
#pipiens2     .......... .......... ......T... .......... .......... .......... .
#pipiens3     .......... .......... ......T... .......... .......... .......... .
```

Tim gene, intron 7-8

```
[                                                                                                                1 ]
[                      1 1111111112 2222222223 3333333334 4444444445 5555555556 6666666667 7777777778 8888888889 9999999990 ]
[             1234567890 1234567890 1234567890 1234567890 1234567890 1234567890 1234567890 1234567890 1234567890 1234567890 ]
#molestus2    GTAGATTGAA TCTTTCTTTC GAGTCCGGAT GAGTTAAAAT TGCACACACT TTGCATGCAC TTTAAATTTG TTCTAAAATC TCAACCCCAC AACTTCACGC
#molestus1    .......... .......... .......... .......... .......... .......... .......... .......... .......... ..........
#molestus3    .......... .......... ....y..... .......... .......... .......... .......... .......... .......... ....k.....
#pipiens1     .......... .......... ....T..... ........r. .......... .......... .......... .......... .......m.. ....r.....
#pipiens2     .......... .......... ....T..... .........y .......... .......... .......... .......... .......... ....G.....
#pipiens3     .......... .......... ....T..... .......... .......... .......... .......... .......... .......... ....G.....

[             1111111111 1111111111 1111111111 1111111111 1111111111 1111111111 ]
[             0000000001 1111111112 2222222223 3333333334 4444444445 5555555556 ]
[             1234567890 1234567890 1234567890 1234567890 1234567890 1234567890 ]
#molestus2    TCCGTTACAG ACCGTTACAA CTCAGAAAAC CCTGTGTGCT TCTTCTTTAC TCCTAAACAG
#molestus1    .......... .......... .......... .......... .......... ..........
#molestus3    .......... .......... .......... .......... .......... ..........
#pipiens1     .......... .......... .......... .y........ .......... ..........
#pipiens2     .......... .......... .......... .......... .......... ..........
#pipiens3     .......... .......... .......... .......... .......... ..........
```

Tim gene, intron 9-10

```
[                                                                                                                1 ]
```

```
[                      1 1111111112 2222222223 3333333334 4444444445 5555555556 6666666667 7777777778 8888888889 9999999990 ]
[             1234567890 1234567890 1234567890 1234567890 1234567890 1234567890 1234567890 1234567890 1234567890 1234567890 ]
#molestus2    GTGCGTGTGA ATCGTGACAA AAATTCCAAA TTTGGTTGAT TTCCCCCACA TATTGTGCTA AACTTGATTC AATAATCCAA ACAATTGCAA CTGAATCGCG
#molestus1    .......... .......... .......... .......... .......... .......... .......... .......... .......... ..........
#molestus3    .......... .......... .......... .......... .......... .......... .......... .......... .......... ..........
#pipiens1     .......... .......... .......... .A........ ......TG.. .......... .......... ...TG..... .......... ........A.
#pipiens2     .......... .......... .......... .A........ .......G.. .......... .......... ...TG..... .......... ........A.
#pipiens3     .......... .......... .......... .A........ ......TG.. .......... .......... ...TG..... .......... ........A.

[             1111111111 1111111111 1111111111 1111111111 1111111111 1111111111 1111111]
[             0000000001 1111111112 2222222223 3333333334 4444444445 5555555556 6666666]
[             1234567890 1234567890 1234567890 1234567890 1234567890 1234567890 1234567]
#molestus2    TTCAGCCAAT CGAGCAAAAA GAAGCAAATA TAACGCCTGT ATTTCTGCAA TTTTCGCCCG TCCGCAG
#molestus1    .......... .......... .......... .......... .......... .......... .......
#molestus3    .......... .......... .......... .......... .......... .......... .......
#pipiens1     .......... T......... .......... .......... .......... .......... .......
#pipiens2     .......... T......... .......... .......... .......... .......... .......
#pipiens3     .......... T......... .......... .......... .......... .......... .......
```
