# Supplementary material for: Comparative analysis of the circadian rhythm genes period and timeless in Culex pipiens Linnaeus, 1758 (Diptera, Culicidae)
Source: Comp Cytogenet. 2016 Oct 10;10(4):483–504. doi: 10.3897/CompCytogen.v10i4.7582 (PMC5240504; doi:10.3897/CompCytogen.v10i4.7582)
Supplement: Supplementary material 5 — Codon-based Test of Neutrality for analysis between per gene sequences of Culex pipiens both forms. [file CompCytogen-010-483-s005.pdf]

Supplemented file 5.

Codon-based Test of Neutrality for analysis between *per* gene sequences of *C. pipiens* both forms.

		1	2	3	4	5	6	7	8	9	10	11	12	13	14	15	16	17	18
1	pipiens1-1		-2,501	0,000	-1,247	-1,247	-2,703	-2,420	-2,420	-2,607	-3,271	-3,392	-3,392	-3,841	-3,841	-3,951	-3,641	-3,282	-3,641
2	pipiens1-2	0,014		-2,501	-2,703	-2,703	-1,756	-3,477	-3,477	-3,610	-2,593	-2,735	-2,735	-3,247	-3,247	-3,374	-3,030	-2,599	-3,030
3	pipiens1-3	1,000	0,014		-1,247	-1,247	-2,703	-2,420	-2,420	-2,607	-3,271	-3,392	-3,392	-3,841	-3,841	-3,951	-3,641	-3,282	-3,641
4	pipiens2-1	0,215	0,008	0,215		0,000	-2,501	-2,505	-2,505	-2,688	-3,546	-3,661	-3,661	-4,095	-4,095	-4,201	-3,756	-3,409	-3,756
5	pipiens2-3	0,215	0,008	0,215	1,000		-2,501	-2,505	-2,505	-2,688	-3,546	-3,661	-3,661	-4,095	-4,095	-4,201	-3,756	-3,409	-3,756
6	pipiens2-2	0,008	0,082	0,008	0,014	0,014		-3,537	-3,537	-3,669	-2,639	-2,780	-2,780	-3,292	-3,292	-3,418	-2,913	-2,471	-2,913
7	pipiens3-1	0,017	0,001	0,017	0,014	0,014	0,001		0,000	-0,999	-3,661	-3,775	-3,775	-4,214	-4,214	-4,319	-3,865	-3,523	-3,865
8	pipiens3-2	0,017	0,001	0,017	0,014	0,014	0,001	1,000		-0,999	-3,661	-3,775	-3,775	-4,214	-4,214	-4,319	-3,865	-3,523	-3,865
9	pipiens3-3	0,010	0,000	0,010	0,008	0,008	0,000	0,320	0,320		-3,775	-3,888	-3,888	-4,319	-4,319	-4,421	-3,974	-3,641	-3,974
10	molestus1-1	0,001	0,011	0,001	0,001	0,001	0,009	0,000	0,000	0,000		-1,725	-1,725	-0,938	-0,938	-1,297	-1,127	0,557	-1,127
11	molestus1-2	0,001	0,007	0,001	0,000	0,000	0,006	0,000	0,000	0,000	0,087		0,000	-1,860	-1,860	-1,598	-0,819	-0,153	-0,819
12	molestus1-3	0,001	0,007	0,001	0,000	0,000	0,006	0,000	0,000	0,000	0,087	1,000		-1,860	-1,860	-1,598	-0,819	-0,153	-0,819
13	molestus2-1	0,000	0,002	0,000	0,000	0,000	0,001	0,000	0,000	0,000	0,350	0,065	0,065		0,000	-0,999	-1,558	-1,240	-1,558
14	molestus2-2	0,000	0,002	0,000	0,000	0,000	0,001	0,000	0,000	0,000	0,350	0,065	0,065	1,000		-0,999	-1,558	-1,240	-1,558
15	molestus2-3	0,000	0,001	0,000	0,000	0,000	0,001	0,000	0,000	0,000	0,197	0,113	0,113	0,320	0,320		-1,292	-1,545	-1,292
16	molestus3-1	0,000	0,003	0,000	0,000	0,000	0,004	0,000	0,000	0,000	0,262	0,414	0,414	0,122	0,122	0,199		-1,600	0,000

17	molestus3-2	0,001	0,011	0,001	0,001	0,001	0,015	0,001	0,001	0,000	0,579	0,878	0,878	0,218	0,218	0,125	0,112		-1,600
18	molestus3-3	0,000	0,003	0,000	0,000	0,000	0,004	0,000	0,000	0,000	0,262	0,414	0,414	0,122	0,122	0,199	1,000	0,112	

The test statistic (*d*N - *d*S) is shown above the diagonal. *d*S and *d*N are the numbers of synonymous and nonsynonymous substitutions per site, respectively. The variance of the difference was computed using the analytical method. Analyses were conducted using the Kumar method (Kimura 1980). The probability of rejecting the null hypothesis of strict-neutrality (*d*N = *d*S) (below diagonal) is shown. Values of *P* less than 0.05 are considered significant at the 5% level. The analysis involved 18 nucleotide sequences. All ambiguous positions were removed for each sequence pair. There were a total of 766 positions in the final dataset. Evolutionary analyses were conducted in MEGA6 (Tamura et al. 2013).
