# Supplementary material for: Comparative analysis of the circadian rhythm genes period and timeless in Culex pipiens Linnaeus, 1758 (Diptera, Culicidae)
Source: Comp Cytogenet. 2016 Oct 10;10(4):483–504. doi: 10.3897/CompCytogen.v10i4.7582 (PMC5240504; doi:10.3897/CompCytogen.v10i4.7582)
Supplement: Supplementary material 6 — Codon-based Test of Neutrality for analysis between tim gene sequences of Culex pipiens both forms. [file CompCytogen-010-483-s006.pdf]

Supplemented file 6. **Codon-based Test of Neutrality for analysis between *tim* gene sequences of *C. pipiens* both forms.**

		1	2	3	4	5	6	7	8	9	10	11	12	13	14	15	16	17	18
1	pipiens1-1		-1,989	-1,989	-1,611	-1,611	-2,802	-1,347	-1,347	-2,634	-2,831	-2,831	-2,831	-2,831	-2,680	-2,831	-2,680	-2,680	-2,680
2	pipiens1-2	0,049		0,000	-2,458	-2,458	-2,456	-2,267	-2,267	-1,846	-2,830	-2,830	-2,830	-2,830	-2,678	-2,830	-2,678	-2,678	-2,678
3	pipiens1-3	0,049	1,000		-2,458	-2,458	-2,456	-2,267	-2,267	-1,846	-2,830	-2,830	-2,830	-2,830	-2,678	-2,830	-2,678	-2,678	-2,678
4	pipiens2-1	0,110	0,015	0,015		0,000	-2,436	-0,998	-0,998	-2,621	-2,999	-2,999	-2,999	-2,999	-2,861	-2,999	-2,861	-2,861	-2,861
5	pipiens2-2	0,110	0,015	0,015	1,000		-2,436	-0,998	-0,998	-2,621	-2,999	-2,999	-2,999	-2,999	-2,861	-2,999	-2,861	-2,861	-2,861
6	pipiens2-3	0,006	0,015	0,015	0,016	0,016		-2,627	-2,627	-2,219	-2,722	-2,722	-2,722	-2,722	-2,575	-2,722	-2,575	-2,575	-2,575
7	pipiens3-1	0,180	0,025	0,025	0,320	0,320	0,010		0,000	-2,796	-3,132	-3,132	-3,132	-3,132	-2,998	-3,132	-2,998	-2,998	-2,998
8	pipiens3-2	0,180	0,025	0,025	0,320	0,320	0,010	1,000		-2,796	-3,132	-3,132	-3,132	-3,132	-2,998	-3,132	-2,998	-2,998	-2,998
9	pipiens3-3	0,010	0,067	0,067	0,010	0,010	0,028	0,006	0,006		-2,572	-2,572	-2,572	-2,572	-2,420	-2,572	-2,420	-2,420	-2,420
10	molestus1-1	0,005	0,005	0,005	0,003	0,003	0,007	0,002	0,002	0,011		0,000	0,000	0,000	-0,998	0,000	-0,998	-0,998	-0,998
11	molestus1-2	0,005	0,005	0,005	0,003	0,003	0,007	0,002	0,002	0,011	1,000		0,000	0,000	-0,998	0,000	-0,998	-0,998	-0,998
12	molestus1-3	0,005	0,005	0,005	0,003	0,003	0,007	0,002	0,002	0,011	1,000	1,000		0,000	-0,998	0,000	-0,998	-0,998	-0,998
13	molestus2-1	0,005	0,005	0,005	0,003	0,003	0,007	0,002	0,002	0,011	1,000	1,000	1,000		-0,998	0,000	-0,998	-0,998	-0,998
14	molestus2-2	0,008	0,008	0,008	0,005	0,005	0,011	0,003	0,003	0,017	0,320	0,320	0,320	0,320		-0,998	0,000	0,000	0,000
15	molestus2-3	0,005	0,005	0,005	0,003	0,003	0,007	0,002	0,002	0,011	1,000	1,000	1,000	1,000	0,320		-0,998	-0,998	-0,998
16	molestus3-1	0,008	0,008	0,008	0,005	0,005	0,011	0,003	0,003	0,017	0,320	0,320	0,320	0,320	1,000	0,320		0,000	0,000
17	molestus3-2	0,008	0,008	0,008	0,005	0,005	0,011	0,003	0,003	0,017	0,320	0,320	0,320	0,320	1,000	0,320	1,000		0,000

18	molestus3-3	0,008	0,008	0,008	0,005	0,005	0,011	0,003	0,003	0,017	0,320	0,320	0,320	0,320	1,000	0,320	1,000	1,000	

The test statistic (*d*N - *d*S) is shown above the diagonal. *d*S and *d*N are the numbers of synonymous and nonsynonymous substitutions per site, respectively. The variance of the difference was computed using the analytical method. Analyses were conducted using the Kumar method (Kimura 1980). The probability of rejecting the null hypothesis of strict-neutrality (*d*N = *d*S) (below diagonal) is shown. Values of *P* less than 0.05 are considered significant at the 5% level. The analysis involved 18 nucleotide sequences. All ambiguous positions were removed for each sequence pair. There were a total of 519 positions in the final dataset. Evolutionary analyses were conducted in MEGA6 (Tamura et al. 2013).
